# The metagenome of the marine anammox bacterium ‘*Candidatus* Scalindua profunda’ illustrates the versatility of this globally important nitrogen cycle bacterium

**DOI:** 10.1111/j.1462-2920.2012.02774.x

**Published:** 2013-05

**Authors:** Jack van de Vossenberg, Dagmar Woebken, Wouter J Maalcke, Hans J C T Wessels, Bas E Dutilh, Boran Kartal, Eva M Janssen-Megens, Guus Roeselers, Jia Yan, Daan Speth, Jolein Gloerich, Wim Geerts, Erwin van der Biezen, Wendy Pluk, Kees-Jan Francoijs, Lina Russ, Phyllis Lam, Stefanie A Malfatti, Susannah Green Tringe, Suzanne C M Haaijer, Huub J M Op den Camp, Henk G Stunnenberg, Rudi Amann, Marcel M M Kuypers, Mike S M Jetten

**Affiliations:** 1 Department of Microbiology, IWWR, Radboud University Nijmegen6525 AJ Nijmegen, the Netherlands; 2 Max Planck Institute for Marine MicrobiologyCelsiusstrasse 1, Bremen, Germany; 3 Nijmegen Centre for Mitochondrial Disorders, Nijmegen Proteomics Facility, Department of Laboratory Medicine, Laboratory of Genetic, Endocrine and Metabolic disease, Radboud University Nijmegen Medical CentreNijmegen, the Netherlands; 4 CMBI, Radboud University Nijmegen Medical CentreNijmegen, the Netherlands; 5 Nijmegen Center for Molecular Life Sciences, Department of Molecular Biology, Radboud University NijmegenNijmegen, the Netherlands; 6 Nijmegen Proteomics Facility, Department of Laboratory Medicine, Laboratory of Genetic, Endocrine and Metabolic disease, Radboud University Nijmegen Medical CentreNijmegen, the Netherlands; 7 DOE Joint Genome InstituteWalnut Creek, CA 94598, California, USA; 8 Department of Biotechnology, Delft University of TechnologyDelft, the Netherlands

## Abstract

Anaerobic ammonium-oxidizing (anammox) bacteria are responsible for a significant portion of the loss of fixed nitrogen from the oceans, making them important players in the global nitrogen cycle. To date, marine anammox bacteria found in marine water columns and sediments worldwide belong almost exclusively to the ‘*Candidatus* Scalindua’ species, but the molecular basis of their metabolism and competitive fitness is presently unknown. We applied community sequencing of a marine anammox enrichment culture dominated by ‘*Candidatus* Scalindua profunda’ to construct a genome assembly, which was subsequently used to analyse the most abundant gene transcripts and proteins. In the *S. profunda* assembly, 4756 genes were annotated, and only about half of them showed the highest identity to the only other anammox bacterium of which a metagenome assembly had been constructed so far, the freshwater ‘*Candidatus* Kuenenia stuttgartiensis’. In total, 2016 genes of *S. profunda* could not be matched to the *K. stuttgartiensis* metagenome assembly at all, and a similar number of genes in *K. stuttgartiensis* could not be found in *S. profunda*. Most of these genes did not have a known function but 98 expressed genes could be attributed to oligopeptide transport, amino acid metabolism, use of organic acids and electron transport. On the basis of the *S. profunda* metagenome, and environmental metagenome data, we observed pronounced differences in the gene organization and expression of important anammox enzymes, such as hydrazine synthase (HzsAB), nitrite reductase (NirS) and inorganic nitrogen transport proteins. Adaptations of *Scalindua* to the substrate limitation of the ocean may include highly expressed ammonium, nitrite and oligopeptide transport systems and pathways for the transport, oxidation, and assimilation of small organic compounds that may allow a more versatile lifestyle contributing to the competitive fitness of *Scalindua* in the marine realm.

## Introduction

Anaerobic ammonium oxidation (anammox) is a microbially mediated process that was predicted in 1977 as an important missing link in nature (Broda, 1977). In this exergonic process, ammonium is oxidized by equimolar amounts of nitrite to nitrogen gas (N_2_) as the final product. In 1995 the process was discovered in a nitrogen-removing bioreactor (Mulder *et al*., 1995), and the responsible group of bacteria was identified a few years later (Strous *et al*., 1999).

The first anammox bacterial cultures were enriched from wastewater treatment environments, and therefore the initial focus of anammox research was on the application of these bacteria (Kartal *et al*., 2010a). However, it soon became clear that marine anammox bacteria are responsible for a significant portion of nitrogen loss from stratified seas and from oceanic oxygen minimum zones (OMZs) where up to half of global marine nitrogen loss takes place (Kuypers *et al*., 2003; 2005; Lam *et al*., 2007; Jensen *et al*., 2011). In these environments, anammox bacteria must compete with aerobic ammonium or nitrite oxidizers for limiting concentrations of ammonium and nitrite (Lam *et al*., 2009; Jensen *et al*., 2011; Yan *et al*., 2011), and with denitrifying bacteria for nitrate and nitrite.

To date, at least five genera of anammox bacteria have been enriched and described, and these form a monophyletic order of the *Brocadiales* that branches deeply in the phylum *Planctomycetes* (Jetten *et al*., 2010). Among these, the deepest branching anammox genus, ‘*Candidatus* Scalindua’ (hereafter referred to as *Scalindua*), is the only representative found in all marine environments investigated worldwide (Schmid *et al*., 2007; Woebken *et al*., 2008). Experimental evidence for this was generated through fluorescence *in situ* hybridization (FISH), amplification of 16S rRNA and functional genes, lipid analysis and molecular surveys (Kuypers *et al*., 2005; Penton *et al*., 2006; Hamersley *et al*., 2007; Schmid *et al*., 2007; Woebken *et al*., 2007; 2008; Sakka *et al*., 2008; Pitcher *et al*., 2011; Stewart *et al*., 2012).

**Fig. 1:**
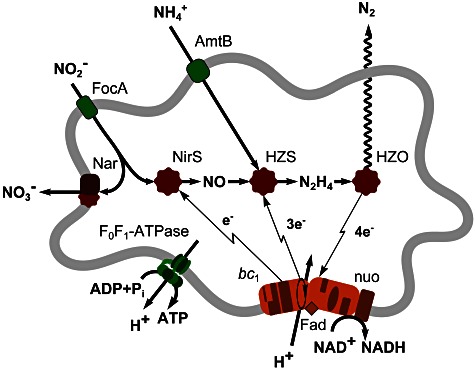
Overview of anammox metabolism in ‘*Candidatus* Scalindua profunda’. Nar/nxr, nitrite::nitrate oxidoreductase; NirS, nitrite reductase; HZS, hydrazine synthase; HZO, hydrazine oxidoreductase; FocA, nitrite transport protein; amtB, ammonium transport protein; nuo, NADH ubiquinone oxidoreductase (complex I).

The first metagenome of an anammox bacterium came from an enrichment culture of ‘*Candidatus* Kuenenia stutt-gartiensis’ in 2006 (Strous *et al*., 2006). *In silico* analysis of this genome assembly led to the postulation of a minimal set of three redox reactions (Strous *et al*., 2006; Kartal *et al*., 2011) essential for anammox catabolism. These three are respectively: (i) reduction of nitrite to nitric oxide by a cd_1_ nitrite reductase (NirS), (ii) condensation of ammonium and nitric oxide into hydrazine by a hydrazine synthase (HZS), and (iii) oxidation of hydrazine into dinitrogen gas by a hydrazine oxidoreductase (HZO; Fig. 1). These reactions were recently verified experimentally with *K. stuttgartiensis* single cells (Kartal *et al*., 2011). Energy conservation is proposed to occur via a chemiosmotic mechanism through electron transfer reactions at the membrane of the internal cellular compartment, involving the cytochrome *bc*_1_ and membrane bound ATP synthase complexes (van Niftrik *et al*., 2010). Carbon assimilation has been predicted to occur via the reductive acetyl-CoA pathway (Strous *et al*., 2006). Anaerobic oxidation of part of the nitrite to nitrate by a nitrate/nitrite oxidoreductase (nxr) complex with high similarity to the nxr system of *Nitrospira* would be needed to drive reversed electron transport (Lücker *et al*., 2010). Transport systems for the import of ammonium and nitrite would proceed to supply the anammox cells with sufficient substrate for their metabolism (Fig. 1).

Nevertheless, the postulation of the essential anammox processes described above was based on the genome and metabolism of *K. stuttgartiensis*, a freshwater species that has never been detected in marine environments. As most marine anammox bacteria belong to the genus *Scalindua* that share less than 89% 16S rRNA gene sequence identity with *K. stuttgartiensis* (Woebken *et al*., 2008), *Scalindua* bacteria are most likely a very specialized group that are well adapted to marine environmental conditions. Hence, the adaptive strategies and metabolic potentials of marine anammox bacteria would not be fully represented by the *K. stuttgartiensis* genome (Jensen *et al*., 2011; Stewart *et al*., 2012). Due to the predicted expansion and intensification of oceanic OMZs in part caused by global climate change, it is increasingly important to understand the nitrogen cycle of our (future) ocean (Stramma *et al*., 2008). Therefore, a comprehensive genomic data set for marine anammox bacteria would be an important asset to understand the competitive fitness of *Scalindua* bacteria in the marine nitrogen cycle under oxygen-limited conditions.

The biomass for the current genome study came from an enrichment of marine *Scalindua* anammox bacteria, here tentatively named ‘*Candidatus* Scalindua profunda’ (van de Vossenberg *et al*., 2008; see Table S1). After 18 months of operation, this *S. profunda* culture started to generate suspended single anammox cells in its effluent which were further purified by density gradient centrifugation. From this purified fraction (99% of *S. profunda* anammox bacteria by FISH count) genomic DNA was isolated, sequenced and assembled (Taxon Object IDs are 2017108002 and 2022004002 at JGI). The genome assembly was used to identify the most important genes and gene products that were expressed under laboratory and *in situ* conditions. We also re-analysed a recently published metatranscriptome data set from the Chilean OMZ based on our new *S. profunda* genome assembly, to further assess the *in situ* expression of *S. profunda* genes.

## Results and discussion

### Overview of sequencing results and genome assembly

The various DNA sequencing efforts on both purified cells and biomass directly from the enrichment culture yielded around 2.7 billion bases in total (Table 1; Taxon Object IDs are 2017108002 and 2022004002 at JGI). This is about 540 times the expected genome size of ‘*Candidatus* Scalindua profunda’, which was estimated to be around 5 million base pairs. From the purified cells, 308 DNA reads (i.e. 0.03% of total) matched with 16S rRNA genes and all belonged to the order of *Brocadiales*. Most (92%) of the reads could be directly assigned to *S. profunda*. This agreed well with the FISH results and suggested that the large majority of the genomic DNA was derived from *S. profunda*. As expected the metagenome data obtained from the sample taken directly from the bioreactor showed a more diverse population and yielded 0.02% (111) reads that matched to 16S rRNA genes. Although FISH of the biomass from the reactor showed about 80% *Scalindua* cells of all DAPI stainable microorganisms, only 38% of the analysed 16S rRNA gene sequences, belonged to *Planctomycetales*/*S. profunda*, while the other 16S rRNA genes were distributed over numerous bacterial phyla. An overview of the diversity of the enrichment culture is presented in Fig. S4 and can be found in the data sets of JGI under Taxon Object IDs 2017108002 and 2022004002 at JGI. This under-representation of anammox has been observed previously in other anammox genome sequencing efforts and may be caused by incomplete DNA extraction or biased cloning of anammox DNA (Strous *et al*., 2006; Gori *et al*., 2011).

**Table 1:** Overview of the sequencing methods used in the ‘*Candidatus* Scalindua profunda’ metagenome project (Taxon IDs at JGI are 2017108002 and 2022004002)

Method	Origin	Type	Number of reads	Total number of bases
454 GS20	Purified cells	Genomic DNA	342 789	36
454 GS20	Purified cells	Genomic DNA	323 065	34
454 GS-Flex	Purified cells	Genomic DNA	455 726	114
454-Titanium	Reactor biomass	Genomic DNA	448 409	132
Sanger paired end	Reactor biomass	Genomic DNA	19 049	14
Sanger paired end	Reactor biomass	Genomic DNA	18 672	15
Illumina Solexa	Reactor biomass	cDNA	31 421 217	2356
Total				2701
				ORFs detected
FT MS/MS	Reactor biomass	Protein		341
FT MS/MS	Reactor biomass	Protein		710

The sequence data from the purified *S. profunda* cells were taken as the starting point for genome assembly and analysis. Assembly of the 184 Mb 454 sequence data (Newbler 2.0) yielded 1580 contigs with a GC content of 39.1% containing 4756 predicted genes (Tables S2 and S3). Binning with MetaCluster (Yang *et al*., 2010) could remove a small number of contigs, resulting in 4741 predicted genes in 1469 contigs of an average length of 7.2 kb (N50 was 8.8 kb). Because the reduction in the number of contigs was low, and because binning was uncertain for the smaller sized contigs, we decided to base all our analyses on the original assembly. Contigs that contained fragmented genes of special interest were compared with assembled metagenome and transcriptome data and curated by hand where possible. The metagenome and transcriptome assemblies were not used to add more genes to the data set but are available under Taxon Object IDs 2017108002 and 2022004002 at JGI for comparison.

**Fig. 2:**
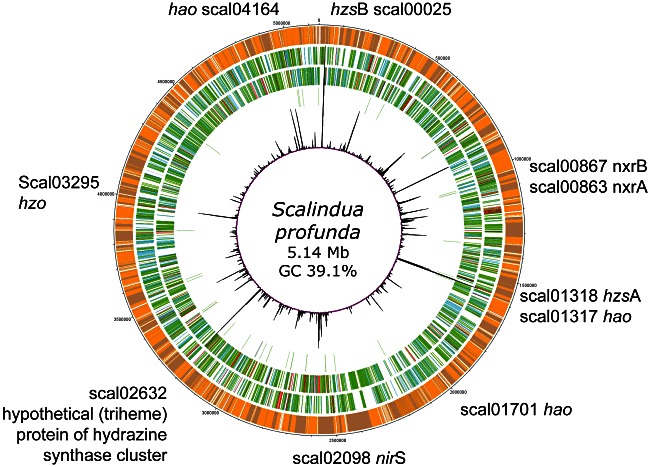
Graphic representation of the ‘*Candidatus* Scalindua profunda’ genome assembly. Depicted from outside to inside are: (i) contigs – alternating brown and ochre; (ii) protein-coding genes – forward; (iii) protein-coding genes – reverse. Legenda of the used colours: red, found in the proteome; green, found in *K. stuttgartiensis*, but not in the proteome; cyan and dark blue, homology with other proteins in the nr database; grey, hypothetical proteins, no hits in the NR database. (iv) rRNA (pink) and tRNA (light green) and (v) inner circle, transcriptome expression pattern. The rRNA, SRP_bact, tmRNA, scRNA and RNAseP were excluded from this circle. Abbreviations used: hzs, hydrazine synthase; hzo, hydrazine oxidase; hao, hydrazine/hydroxylamine oxidoreductase; nirS, nitrite reductase; and nxr, nitrite::nitrate oxidoreductase.

Mapping of transcriptome (Fig. 2) data resulted in 3347 matches with annotated genes, i.e. 70% of all predicted genes (Table 2; Table S4 for complete overview). The *S. profunda* genome assembly contained 3 rRNA, 43 tRNA, 1 tmRNA, 2 ncRNA and 1 RNase P. After a preliminary run which detected 341 ORFs, the second liquid chromatography MS/MS analysis of *S. profunda* cell extract showed that 710 annotated ORFs, i.e. 15% of the predicted proteome, have peptide hits in the proteome data (Fig. 2; Tables 1 and 2; Table S5). The function of 1271 genes could be directly assigned via the KEGG website (Kanehisa, 2002). According to the KEGG results, 154 of these genes were involved in energy metabolism, of which 39 in carbon fixation. Twenty-one genes were classified as being involved in nitrogen metabolism, but KEGG was not able to classify genes considered important for the nitrogen conversion in anammox.

**Table 2:** Overview of the most important genes and ORFs detected in the transcriptome and proteome of ‘*Candidatus* Scalindua profunda’ and in the Chili oxygen minimum zone (see *Experimental procedures* for details)

Annotation	Gene	Gene id	*Scalindua*		*Scalindua*		Chili OMZ station 3 depth 200 m			
		
			Transcriptome illumina reads	Transcriptome, relative coverage	Proteome # peptides	Proteome emPAl	cDNA reads	Relative coverage	DNA reads	Relative coverage
NO_3_^-^/NO_2_^-^ antiporter	*narK*	scal03007	216	1.2	–	–	0	0.0	0	0.0
Nitrate reductase	*narG*	scal00863	2 052	4.3	54	5.1	3	0.2	42	2.7
Nitrate reductase	*narH*	scal00867	1 827	9.5	19	3.4	0	0.0	4	0.7
NO_2_^-^ transport protein	*focA*	scal00416	216	1.4	2	0.5	15	1.9	22	2.3
cd1 NO_2_^-^ reductase	*nirS*	scal02098	5 239	20.6	27	2.8	0	0.0	0	0.0
Octahaem HAO	*hao*	scal00421	2 010	8.0	14	1.2	3	0.4	6	0.8
Octahaem HAO	*hao*	scal01317	5 662	25.0	12	6.2	1	0.1	5	0.7
Octahaem HAO	*hao*	scal02110	1 812	8.0	8	0.4	0	0.0	2	0.3
Octahaem HAO	*hao*	scal02116	657	3.3	16	1.5	1	0.2	19	3.1
Octahaem HAO	*hao*	scal04164	1 529	9.9	10	1.0	0	0.0	0	0.0
NH_4_^+^ transport protein	*amtB*	scal00587	387	1.9	–	–	7	1.2	11	1.8
NH_4_^+^ transport protein	*amtB*	scal00591	68	0.3	–	–	4	0.7	1	0.2
NH_4_^+^ transport protein	*amtB*	scal00594	162	0.8	–	–	0	0.0	5	0.8
NH_4_^+^ transport protein	*amtB*	scal00596	261	1.0	–	–	0	0.0	0	0.0
Hydrazine synthase	*hzsB*	scal00025	19 176	68.2	29	3.5	31	8.9	30	4.0
Hydrazine synthase	*hzsA*	scal01318	8 062	22.8	33	5.4	19	1.8	15	1.4
Octahaem HZO	*hzo*	scal03295	8 341	37.2	23	5.6	183	26.3	13	1.9

### Comparison of *S. profunda* genome assembly to *K. stuttgartiensis* assembly

Intriguingly, although the number of predicted genes (4756) in the assembly of *S. profunda* is in the same order as the 4664 genes present in the *K. stuttgartiensis* assembly, only 693 genes in the *S. profunda* assembly could be found in *K. stuttgartiensis* with blastn (Expect value < 10^−3^) and about half of the ORFs (2740) could be matched with blastp (Expect value < 10^−6^). The *S. profunda* assembly contained 2016 ORFs that had no blastp hit (Expect value < 10^−6^) to the *K. stuttgartiensis* genome assembly. Many (677) of those ORFs had no hit at all in the non-redundant NCBI database (January 2012). Interestingly, 38% of the ORFs that no match to *K. stuttgartiensis* had its closest orthologue in marine microorganisms, possibly reflecting the long evolutionary history of the *Scalindua* anammox bacteria in marine ecosystems (Klotz *et al*., 2008; Klotz and Stein, 2008). About the same number (2187) of ORFs in the *K. stuttgartiensis* genome did not have a match to any ORF in the *S. profunda* genome. A complete overview of the metagenome data best blast hits can be found under taxon Object IDs 2017108002 and 2022004002 at JGI. A MAUVE analysis (Fig. S1) confirmed that about 2.5 Mb of the *S. profunda* genome could not be aligned with the five *K. stuttgartiensis* supercontigs, and that the homology of the ORFs was on average only 48.6% on amino acid level.

About 98 expressed genes (Table S6) of particular interest, not found in *K. stuttgartiensis*, could be attributed to several metabolic functions such oligopeptide transport, amino acid metabolism, electron transport, use of organic acids, carbonic anhydrase, detoxification of nitric oxide and cell attachment, possibly defining some unique properties of *Scalindua* bacteria.

Interestingly, a quinol-oxiding qNor NO-reductase (scal02135), partial norB (scal00292) and a highly expressed putative norVW flavodoxin (scal000274) protein were identified. The presence and expression of these genes suggests that *S. profunda* bacteria may experience nitric oxide stress in their environments (Almeida *et al*., 2006) although freshwater species have been shown to be resistant up to 5000 ppm nitric oxide (Kartal *et al*., 2010a, b). The genome assembly of *S. profunda* contained two genes for ribulose bisphosphate carboxylase-like proteins (Rubisco-like protein, RLP; scal00245 and scal03046) in a 13 kb contig. Transcriptome data show that scal00245 is expressed at 0.23 relative coverage. The protein sequence of scal00245 clusters with Rubisco Form IV sequences, and is most closely related to *Candidatus* Magnetobacterium and *Planctomyces limnophilus* RLPs. Like other RLPs scal00245 lacks essential residues at positions that are importantfor catalysis (Tabita *et al*., 2007). The function of other Form IV sequences is not yet known and needs further investigation.

At least three copies of genes that code for carbonic anhydrase (scal00602, scal01206 and scal03005), which catalyses the interconversion between CO_2_ and HCO_3_^-^ were identified in the genome assembly. Only scal01206 was found to be expressed at appreciable levels (0.27 rel cov). Like in acetogenic bacteria, which also use the reductive acetyl-CoA pathway, the physiological role of carbonic anhydrase in anammox bacteria may be to increase the intracellular CO_2_ levels or to regulate internal pH (Braus-Stromeyer *et al*., 1997).

The role of the additional 31 cytochrome *c* and iron sulfur cluster-encoding genes in *S. profunda* is presently unknown. But together with the heterodisulfide reductase-like genes cluster and two hydrogenase gene clusters they might be involved in electron transport from hydrogen or formate. A further 26 and 13 genes were putatively involved in carbon metabolism or oligopeptide and amino acid transport and are discussed below. Twenty ORFs encode for transport proteins of which several copper ABC transport-encoding genes are highly expressed at 2.5–8.4 relative coverage, indicating a high requirement for copper under the cultivated conditions. Finally several *pil*T-like genes possibly involved in cell attachment or in cell-to-cell communication were present in *S. profunda* which is consistent with present of pili like structure in electron microscopic pictures of *Scalindua* cells (van de Vossenberg *et al*., 2008).

### Genomic basis for anammox reactions in *S. profunda*

Transcriptome and proteome data showed that the most highly expressed and translated genes code for proteins (Figs 1 and 2) involved in the conversion of nitrogen compounds and carbon metabolism (Table 2). In the next section, hydrazine metabolism, nitrite and nitrate conversion, transport of inorganic nitrogen compounds and respiration will be discussed.

### Hydrazine metabolism

**Fig. 3:**
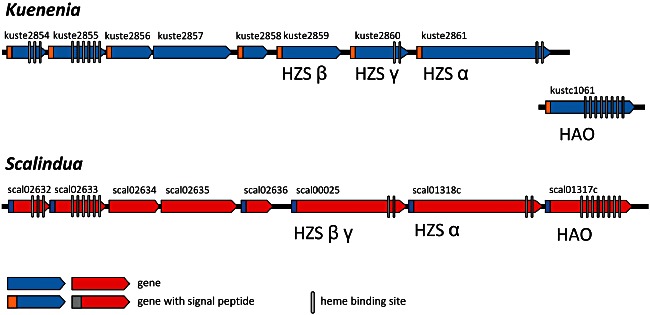
Organization of hydrazine synthase complex in *Kuenenia stuttgartiensis* and *Scalindua profunda*. In *S. profunda* the subunits β and γ of hydrazine synthase (HZS) are fused into one polypeptide HZS βγ. HAO is hydroxylamine oxidoreductase.

Hydrazine synthase is the enzyme that is responsible for one of the key features of anammox bacteria (Fig. 1): the condensation reaction of ammonium with NO to make hydrazine (Strous *et al*., 2006). The *K. stuttgartiensis* genome contains a cluster of three genes that code for HZS (kuste2859–2861) (Kartal *et al*., 2011). It appeared that the *S. profunda* orthologues of the β-propeller-encoding gene and a gene coding for a dihaem containing homologue of cytochrome *c* peroxidase (kuste2859 and kuste2860 in *K. stuttgartiensis* respectively) were fused into one single gene (scal00025; Fig. 3). The protein, HZS βγ-subunit, was detected on SDS-PAGE gel as a protein of around 75 kDa (predicted mass 74.3 kDa; Fig. S2), and its identity was confirmed by MALDI-TOF analysis. The α-subunit of HZS (scal01318) was identified as the homologue to kuste2861. The two *hzs* genes were expressed at the highest level of *S. profunda* genes. Of all cDNA reads that mapped with the genome assembly 5% could be assigned to the two *hzs*AB genes (scal00025 and scal01318).

The *S. profunda* genome contains an octahaem *hzo/hao* gene (scal01317) directly downstream of the α-subunit of HZS. Anammox bacterium KSU-1, a species detected in an anaerobic ammonium-oxidizing biofilm (Fujii *et al*., 2002), possesses an *hzo/hao* gene in the same position (BAF98478, downstream of the α-subunit of HZS BAF98477). However, in the *K. stuttgartiensis* genome the orthologue gene (kustc1061) is present in a different location of the genome. The *hao* gene product for KSU-1 was isolated and it was found to be quite similar with a previously isolated HAO protein from *Brocadia anammoxidans* (Schalk *et al*., 2000; Shimamura *et al*., 2008). The KSU-1 enzyme showed a high catalytic activity in a cytochrome *c*-dependent hydroxylamine oxidation, while its affinity for and oxidation rate of hydrazine were significantly less. That particular HAO might function in the removal of hydroxylamine formed during turnover of substrate by the HZS. Thus, this HAO would aid in keeping the hydroxylamine concentration below inhibitory thresholds. Like in *K. stuttgartiensis* (kustc1061 and kusta0043), the *S. profunda* genome contained paralogues of the HAO-coding genes scal01317 and scal02116.

The four-electron oxidation of hydrazine to nitrogen gas has been shown to be catalysed by an octahaem HAO-like protein, hydrazine oxidoreductase (HZO) (Kartal *et al*., 2011). In KSU-1, one HZO-like protein (hzoB BAF98481, which differs only in two amino acids with hzoA BAF36964) catalysed the oxidation of hydrazine, and was inhibited by low concentrations of hydroxylamine (Shimamura *et al*., 2007). The orthologues in *K. stuttgartiensis* are kustc0694 and kustd1340. In the *S. profunda* assembly only one copy, scal03295, could be identified. The high coverage of the gene in the assembly and transcriptome and the presence of 11 SNPs (Table S8) in the gene might indicate that in *S. profunda* also two copies could exist. However, the short 454 and illumina reads cannot resolve this issue in the present assembly. The *hzo* scal03295gene is highly expressed (Table 2) and its gene product is abundantly present in the proteome. Moreover it is one of the highest expressed genes in the oxygen minimum zone (Stewart *et al*., 2012, see last paragraph of ‘In situ *gene organization and expression of S*. profunda *genes*’ for more details).

Similar to *K. stuttgartiensis* (De Almeida *et al*., 2011), the genome of *S. profunda* possesses a large number of other octahaem HAO-like proteins, of which some are clustered with cytochrome *c* proteins that may be involved in electron transfer. The *S. profunda* genome contains nine orthologues for all *K. stuttgartiensis* HAO-like proteins, except for kuste2457, and all of these genes are expressed. Paralogues of *hao* genes may be involved in detoxification of potentially hazardous nitrogen compounds (Kartal *et al*., 2011). Alternatively, one or more of the HAO proteins may have an ammonia-forming capacity like in the epsilon proteobacterium *Nautilia* (Campbell *et al*., 2009). Based on sequence homology the most likely candidate ORF for an ammonia forming HAO protein would be scal02288. This enzyme could be part of the molecular inventory that renders *Scalindua* the capability to perform dissimilatory nitrate/nitrite reduction to ammonia (DNRA) (see below).

### Nitrate and nitrite conversion

Nitrate reductase nxrA (scal00863) has the highest number of peptide hits in the *S. profunda* proteome, and its mRNA is abundantly present in the transcriptome (Table 2). NxrA/narG catalyses reduction of nitrate to nitrite, but in anammox bacteria this enzyme may likely act in reverse which would result in oxidation of nitrite to nitrate anaerobically; similar to the closely related NXR enzyme in *Nitrospira defluvii* (Lücker *et al*., 2010). The resulting electrons would subsequently be used to feed a reversed transport chain from cytochrome *c* to the quinol pool (Strous *et al*., 2006; Jetten *et al*., 2009). In addition, anammox bacteria may also use this NXR complex as a true nitrate reductase when oxidizing small organic molecules with nitrate as electron acceptor (Kartal *et al*., 2007).

The genes of the *nxr* cluster scal00868–scal00861 are located on the same strand of the same large 84 kb contig and are highly expressed. These gene clusters contained the α-subunit *nxr*A (scal00863), *nxr*D (scal00865), the β-subunit *nxr*B (scal00867) and *nxr*M (scal00868) that have a high similarity to their homologues in *K. stuttgartiensis* (De Almeida *et al*., 2011). The genes for mono- and dihaem proteins that are potentially involved in electron transport to *nxr*AB or vice versa were located in a gene cluster on another 34 kb contig (scal00689–694). In addition, the genome assembly of *S. profunda* contains more genes (scal00048, scal00659, scal00812, scal01552, scal01643, scal02743, scal03485) that may encode for molybdopterin oxidoreductase proteins, potentially involved in nitrate, formate or carbon monoxide conversion, but their exact role needs further study (Boyington *et al*., 1997; Ragsdale, 2004).

Nitrite fulfils multiple functions in the anammox metabolism. It can be converted to nitric oxide by cd_1_*nir*S nitrite reductase, providing the HZS with NO. The nirS gene, scal02098, is located in a gene cluster with three *nir*J-like *S*-adenosylmethione radical proteins involved in haem d_1_ synthesis. Clear *nir*NF homologues were absent, but genes *nir*D and *nir*H which are thought to be involved in haem d_1_ maturation were expressed. The *nir*S gene and protein of *S. profunda* is highly expressed in both transcriptome and proteome, indicating that this protein may indeed be important to produce the NO necessary for the HZS reaction. The *nir*S of *S. profunda* is most closely related to two *Chloroflexi* sequences (Anaerolina NC_014960 and Roseiflexus NC_009767) annotated as hydroxylamine reductases, and might indicate that this *nir*S gene was acquired by lateral gene transfer after the NO detoxifying mechanisms were established (Klotz and Stein, 2008; Klotz *et al*., 2008). Furthermore, the *S. profunda nir*S mRNA expression has been detected in both Peruvian and Arabian Sea OMZs, where its expression levels substantially correlated with anammox rate measurements (Lam *et al*., 2009; Jensen *et al*., 2011; Lam and Kuypers, 2011).

When organic acids are present and ammonium is limited, nitrite is proposed to be converted to ammonium by a multihaem protein complex (Kartal *et al*., 2007). Like *K. stuttgartiensis*, the *Scalinuda* genome assembly contained a tandem of three genes that encode a penta-, deca- and another pentahaem containing proteins, respectively (scal00149–scal00151), but their expression is relatively low. Alternatively one of the HAO proteins, i.e. gene scal02288, may also be involved in conversion of nitrite into ammonium (see above).

### Transport of nitrogen intermediates, amino acids and oligopeptides

Ammonium and/or nitrite may be limiting in the oceanic environment (Lam and Kuypers, 2011). Under anoxic conditions, marine anammox bacteria may depend on DNRA or partial nitrate reduction for their anammox substrates. Under low-oxygen conditions, anammox bacteria will compete for ammonium with aerobic ammonium-oxidizing archaea or bacteria, and for nitrite with nitrite-oxidizing or ammonifying bacteria (Füssel *et al*., 2011). It is therefore proposed that anammox bacteria must be well equipped with genes that code for membrane proteins involved in the uptake of inorganic nitrogen compounds.

### Ammonium transport

Ammonium may be one of the likely limiting factors for anammox bacteria in the OMZs. Species with high-affinity ammonium transport would have a selective advantage in such an environment. The *S. profunda* genome contains four 12-transmembrane helices encoding AmtB ammonium transport proteins in a gene cluster with two P-II regulatory proteins GlnK (scal00587–scal00576). In addition, the *S. profunda* genome also contained two partial genes for ammonium transporters, lacking the C-terminus (*amt*-2 and *amt*B–His–kinase fusion, scal01681 and scal03708 respectively). Gene scal03708 did not have a predicted signal peptide, and both scal03708 and scal01681 proteins were predicted to have only 11 membrane spanning helices. The scal03708 gene is highly expressed under anaerobic conditions in a steady-state culture with about 5 mM surplus ammonium in the medium. According to the study of Medema and colleagues (2010), all anammox AmtB proteins would be targeted to the anammoxosome membrane, except scal00596 and scal01681 (the kustc1009 homologues), which would be most likely located at the cytoplasmic membrane. The genes scal00596 and 01681 show 237 and 304 reads in the transcriptome, respectively, which may indicate that *S. profunda* expresses at least two ammonium transport proteins to scavenge ammonium. Taken together, *Scalindua* seem well equipped to transport ammonium into the cells. High expression levels of the *amt*B genes of *S. profunda in situ* was confirmed by re-analysis of metagenome data from the Chilean oceanic oxygen minimum zone (see below) (Stewart *et al*., 2012).

### Nitrite transport

In addition to ammonium, nitrite may also be limiting for anammox bacteria (Füssel *et al*., 2011; Lam and Kuypers, 2011). The *S. profunda* genome has four genes encoding transporter proteins from the Formate/Nitrite Transporter (FNT) family (Saier Jr *et al*., 1999), FocA/NirC, with six predicted membrane spanning helices, but no apparent predicted signal peptide (SignalP). Structure analysis of the formate transporter FocA revealed that the protein assembles into a homo-pentamer which acts like a channel instead of an active transporter (Waight *et al*., 2010). NirC mediates high-flux transport of nitrite across the inner membrane in both directions in *Escherichia coli*, but the transport mechanism is yet unknown. In the *S. profunda* genome, gene products of scal00416 and scal04132 have higher similarity to *E. coli nir*C than *foc*A, and interestingly both genes are located near HAO coding genes (scal00421 and scal04133 respectively), possibly in an operon. The gene products of scal00974 and scal00975 are more similar to *foc*A and not located near an *hao* gene. In comparison, the *K. stuttgartiensis* genome contains only one gene that clusters with *E. coli nir*C, kuste3055, which is not located near a HAO gene. Furthermore, the *K. stuttgartiensis* genome has five genes that code for proteins that cluster with FocA and none of the genes is found with a nearby HAO (kusta0004, kusta0009, kustd1720, kustd1721 and kuste4324). Similar to the *amt*B genes, high expression levels of FocA (Table 2) were also observed *in situ* in environmental samples of the Chilean OMZ (see below), indicating that in this OMZ *Scalindua* may experience severe nitrite limitation.

### Nitrate transport

The *S. profunda* genome contained only one gene for a *nar*K type I transporter (scal03007), a secondary transport protein belonging to the Major Facilitator Superfamily. The *K. stuttgartiensis* genome contains three *nar*K genes (kuste2335, kuste2308 and kustd2047), of which the first two are highly similar with scal03007 (*E*-value < 10^−140^). Bacterial NarK proteins can be divided over two distinct subgroups, Type I and II (Moir and Wood, 2001). Type II would be responsible for transport of nitrite, which is supported by biochemical evidence (Rowe *et al*., 1994). Because Type II *nar*K genes are found adjacent to nitrite assimilatory genes, and Type I *nar*K genes are found near genes for assimilatory nitrate uptake, Moir and Wood (2001) postulated that Type I would then transport nitrate. In the *S. profunda* genome, scal03007 is flanked by genes that cannot be assigned to nitrate assimilation. In anammox bacteria, nitrite uptake can be accomplished with FocA, and NarK could then function as nitrate transporter.

### Dipeptide and oligopeptide transport

In contrast with the *K. stuttgartiensis* genome, the genome of *S. profunda* contained many genes involved in oligopeptide transport systems. These include a complete dipeptide (Dpp) ABC transport system (scal03998–4002), a complete oligopeptide (Opp) ABC transport system (scal00621–624), and possibly an oligopeptide transporter that belongs to the OPT family (scal00331). All these oligopeptide-encoding genes are expressed at moderate levels (0.1–0.9 relative coverage) by *S. profunda* under laboratory conditions. The presence of these transporters suggests that degraded proteins, possibly originating from sinking and mineralized organic matter from the oxic or pelagic zone, may be used directly by *Scalindua* bacteria for assimilation into cell material or as alternative ammonium source for the anammox reaction. The genes for oligopeptide transport were also detected on a fosmid retrieved from the Peruvian OMZ and appeared to be in close vicinity of the *S. profunda* ribosomal RNA operon (see below).

### Respiratory complexes and metabolic versatility

From the genome information for *K. stuttgartiensis* it appeared that anammox bacteria have a metabolic versatility that is comparable with those of *Geobacter* and *Shewanella* which are able to use a range of electron donors and acceptors (Heidelberg *et al*., 2002; De Almeida *et al*., 2011). The *S. profunda* genome contained two putative citrate synthase genes (scal03477 and scal01583) that are expressed at moderate levels in mRNA and proteome. These enzymes would enable the oxidation of acetate or propionate after activation by acetate kinase (scal00350) or acetyl coenzyme A synthase (scal02020) via the TCA cycle, a route that is also used by many iron(III) and manganese(IV) reducing microorganisms (Lovley *et al*., 2004). Citrate synthase has not been found in the *K. stuttgartiensis* metagenome that contains five gaps. Similar to *K. stuttgartiensis*, the genome of *S. profunda* contains the genes that code for the complete reductive acetyl-CoA (Wood-Ljungdahl) pathway. All genes are highly expressed and most gene products are found in the proteome. The genes of the CO dehydrogenase/Acetyl-CoA synthase complex were found in one large gene cluster (scal02484–02491). In anammox bacteria, formate can be activated via tetrahydrofolate-dependent pathway. Proteins for this pathway are encoded by scal02521 (formyltetrahydrofolate synthetase), scal0081 (fchA methenyltetrahydrofolate cyclohydrolase) and scal01287 (5,10-methylenetetrahydrofolate reductase).

The energy-rich electrons generated by the oxidation of hydrazine (Fig. 1) need to be funnelled into a respiratory network. Most of the genes encoding for proteins of the respiratory complexes were found to be abundantly present in the *S. profunda* transcriptome and proteome. These included complex I (*nuo* genes), several orthologues of the *bc*_1_ complex, at least two ATPase gene clusters and many cytochrome *c* proteins. Similar to *K. stuttgartiensis*, *S. profunda* uses the type II cytochrome *c* maturation pathway including *res*A (scal00012, scal00014; scal02124; scal02421), *res*B (scal00630) and *res*C (scal00338; scal00629) genes. In the *S. profunda* genome assembly no less than 85 genes encoding for mono-, di- or multihaem cytochrome *c* proteins were identified (Table S7) underlining the high potential for a versatile respiration.

To confirm some of the genome-based predictions on the metabolic versatility, physiological experiments with purified cell suspensions were performed. In the presence of formate, acetate or propionate, *Scalindua* cells could also reduce nitrate and nitrite to dinitrogen gas. Upon addition of ^15^N-nitrate in the presence of an external unlabelled ammonium pool, ammonium became rapidly labelled as was previously documented for freshwater anammox bacteria (Kartal *et al*., 2007). Based on these two observations it is likely that (marine) anammox bacteria can reduce nitrate via nitrite to ammonium using organic matter, mimicking dissimilatory nitrate reduction to ammonium (DNRA) (An and Gardner, 2002; Jensen *et al*., 2011). The labelled ammonium and nitrite endogenously produced in these tests was converted to nitrogen gas via hydrazine. In this way, anammox bacteria could be wrongly recognized as conventional denitrifying bacteria, i.e. ^15^N nitrate may end up as ^30^N_2_ and is mistaken as a signature for denitrification in field experiments such as found in the Arabian Sea OMZ (Jensen *et al*., 2011). The capacity for formate-dependent Mn(IV) and Fe(III) reduction has been observed previously in cell suspensions of *Scalindua* (van de Vossenberg *et al*., 2008). In *Shewanella putrefaciens*, the gene product FerE, member of the PulE family of proteins the type II secretion pathway needs to be expressed for iron and manganese reduction (DiChristina *et al*., 2002). This is necessary for transport of an outer membrane haem-containing protein that is involved in iron(III) reduction. Ten genes in the *S. profunda* genome code for proteins belonging to the PulE family of proteins, of which six are in a gene cluster/operon with *Pul*DFGJK coding genes. On the protein level, three of these genes show very high similarity to *Sh. putrefaciens fer*E (scal00844, scal01671, scal03400), of which scal00844 and scal01671 are found in the transcriptome. The genome of *S. profunda* codes for 42 identified *pul* genes. In *Sh. putrefaciens*, outer membrane cytochromes MtrC and OmcA are supposed to be terminal reductases in iron(III) reduction (Beliaev *et al*., 2001), but orthologues for these genes have not yet been identified in the *S. profunda* and *K. stuttgartiensis* genomes. However, in *S. profunda* the product of scal01344, a cytochrome *c* that has no less than 12 haem-binding motifs is a possible functional candidate for such a terminal reductase. The gene is highly expressed and its product is found in the proteome. A homologue gene is not found in the *K. stuttgartiensis* genome. Like MtrC, this protein has a signal peptide, no other predicted transmembrane helices, and a predicted prokaryotic membrane lipoprotein lipid attachment site profile. This indicates that the protein is translocated across the membrane and modified post-translationally into a lipoprotein. Another candidate for this function would be scal00686, with eight haem motifs, found in the proteome and transcriptome. NapC/NirT cytochrome *c*-encoding genes that can act as electron transfer intermediates in this system are also encoded in the genome of *S. profunda*. Taken together the physiological, and genome data suggest that *Scalindua* employs a versatile metabolism that may contribute to its fitness in natural marine habitats where electron acceptors may be very limiting (Lam and Kuypers, 2011).

### *In situ* gene organization and expression of *S. profunda* genes

In order to compare the genome organization of the present assembly with *in situ* marine *Scalindua* bacteria, biomass from the Peruvian Oxygen Minimum Zone was filtered and used for DNA extraction and building of a fosmid library. The fosmid library was screened for anammox-bacterial 16S rRNA genes (Woebken *et al*., 2007) and in this way two fosmids (mey3 and mey4) containing a *Scalindua* 16S rRNA gene were found and fully sequenced (Fig. S3A and B). The 16S and 23S ribosomal genes on the fosmids were 98.1% and 97.5% similar to each other confirming the microheterogeneity observed previously by ITS sequencing of anammox 16S–23S rRNA clones from the Peruvian OMZ (Woebken *et al*., 2008). Furthermore, about 1000 fosmids were end sequenced, and the sequences were compared with the *K. stuttgartiensis* and *S. profunda* genome assembly as soon as it became available (see below). In this way two more fosmids (PC46A10 and PC60G12; Fig. S3C and D) were retrieved and fully sequenced.

In addition to the rRNA operon, the mey3 and mey4 fosmids contained the four-gene cluster for oligopeptide transport (scal00621–00624) indicating their importance *in situ*. Mapping of the *S. profunda* genome contigs using MAUVE to the fosmids showed a high conservation in gene order and a very high sequence identity (see Fig. S3A–D). In some cases this contig alignment to the fosmids made re-arrangements of the *S. profunda* contigs into larger scaffolds possible. Analysis of fosmid PC60G12 revealed the presence of three *amt*B and two PII genes involved in ammonium transport in a large gene cluster in a similar gene organization as in the *S. profunda* genome assembly. Fosmid PC46A10 contained several genes encoding proteins involved carbon metabolism of anammox: acetate kinase, phosphotransacetylase, pyruvate kinase and pyruvate ferredoxin oxidoreductase, indicating that the potential for a versatile carbon metabolism is also present *in situ*.

Recently, a survey of metagenome and transcriptome data from samples obtained from different depths of the OMZ in the Eastern Tropical South Pacific, where *Scalindua* is the dominant anammox genus were published (Stewart *et al*., 2012). Even though the only available anammox genome information at that time came from *K. stuttgartiensis*, the authors could assign many of the reads to anammox genes, albeit at low bit scores. As is apparent from the present study, *K. stuttgartiensis* gene content and composition is quite different from *S. profunda*. Therefore we re-analysed some of the transcripts of the OMZ survey, using the data of station 3 at the core OMZ where *Scalindua* reads were most abundant in the libraries (i.e. station #3 at 200 m water depth, Table 2). After a blastx run of the total number of 441 273 cDNA reads against a database of predicted *S. profunda* gene products, 12 669 reads matched with *E*-values below 10^−9^. With the subset of matching reads, we performed another blastx search against the NCBI NR database, and *E*-values were compared between both runs. In the NR search, 3860 reads (33%) had a best match with the known anammox bacteria *K. stuttgartiensis* (3440 reads) and KSU-1 (420 reads). However, when *E*-values were compared between the NR and *S. profunda* searches, 7813 reads (62%) had a best hit against *S. profunda* leaving only 40 reads as best hit for *K. stuttgartiensis* and not more than one for KSU-1. It is clear that sequences from OMZ samples are much more similar to *S. profunda* than to *K. stuttgartiensis*. More importantly, with the genome of *S. profunda* as a template, many more reads from OMZ environmental data could be assigned to anammox bacteria.

The *Scalindua* gene in the OMZ data with highest read coverage was the *hzo* (scal03295) followed by the *hzs*βγ-subunit (scal00025), similar to the expression data observed under laboratory conditions. Many of the other most highly expressed anammox genes in the OMZ were directly involved in the central metabolism and ammonium transport of anammox. The high expression of both ammonium and nitrite transport proteins may reflect the substrate limitation of the *Scalindua* cells in the OMZ that is apparent from the nutrient profiles made at various stations (Lam *et al*., 2009; Canfield *et al*., 2010). In order to obtain more detailed information on the expression of anammox genes under substrate limitation and oxygen exposure, further studies on co-cultures of marine nitrifiers and anammox bacteria should be performed.

### Conclusion

The genome of *S. profunda* revealed that this important marine anammox bacterium is very different from its freshwater counterparts. It appears to have the greater ability to utilize small organic acids and oligopeptides and may use nitrate, nitrite and metal oxides as terminal electron acceptors. The high expression of ammonium and nitrite transport proteins may reflect their high capacity to take up essential substrates (ammonium and nitrite) despite their relatively low concentrations usually found in marine environments. The combined results from this study on *S. profunda* gave us the much needed insights to design experiments to better understand the competitive fitness of this globally important organism in marine ecosystems.

## Experimental procedures

### Biomass origin of marine *Scalindua* and growth conditions

The basis for the current study was an enrichment derived from a marine sediment taken from a Swedish Fjord (van de Vossenberg *et al*., 2008). The enrichment of marine anammox bacteria was obtained in an anoxic sequencing batch bioreactor (SBR), fed with water containing sea salt and the substrates ammonium, nitrite and carbonate (van de Vossenberg *et al*., 2008). After 18 months of operation, equivalent to 15–25 generation times for anammox bacteria, this bioreactor enrichment started to generate suspended single anammox cells in its effluent. The effluent was collected overnight and these single cells were further purified by density gradient centrifugation. FISH cell counts of the purified fraction revealed that at least 99% of the cells consisted of *S. profunda* anammox bacteria. From these cells we isolated 10 µg of genomic DNA that was subsequently used for 454 pyrosequencing (Table 1). Additional DNA for analysis of the entire metagenome was extracted from the enrichment culture directly, and shotgun and fosmid libraries were constructed and sequenced (Kartal *et al*., 2011). Furthermore community DNA was sequenced by 454 Titanium technology (Table 1). The sequencing data and assembly are available at JGI under Taxon ID 2017108002 and 2022004002 respectively.

RNA for the transcriptome, and proteins for the proteome, came directly from the enrichment culture to minimize the induction of stress response in the *Scalindua* cells. Transcriptome data were obtained by Illumina sequencing of cDNA according to Kartal and colleagues (2011). Proteome data were obtained after separation of denatured proteins on an SDS-PAGE gel or liquid chromatography followed by peptide identification using tandem online mass spectrometry (Kartal *et al*., 2011).

### FISH analysis

The purity of the sample and the identity of the cells was monitored by FISH microscopy. Epifluorescence was used for identification of the anammox cells, using *Scalindua*-specific FISH probes S-*-BS-820-a-A-22 (BS820), S-*-Scama-820-a-A-22 (ScaMa820), S-*-Apr-0820-a-A-21 (Apr820), anammox genera-specific probe S-*-Amx-0820-a-A-22 (Amx820), S-*-Amx-0368-a-A-18 (Amx368), and *Planctomycetes*-specific probe S-P-Planc-0046-a-A-18 (Pla46) and DAPI as general DNA stain (Schmid *et al*., 2005; van de Vossenberg *et al*., 2008).

### Genomic DNA

Single cells were collected overnight from the effluent of a 15°C batch bioreactor of 2 l, fed with 1 mmol day^−1^ of both nitrite and ammonium in Red Sea Salt medium (van de Vossenberg *et al*., 2008). Cycloheximide (0.3 g l^−1^) was added to the effluent bottle to prevent protozoa growth. A density gradient was prepared of 4 ml of 10 mM phosphate-buffered growth medium pH 7.4 and 5 ml of Percoll (Amersham Pharmacia Biotech, the Netherlands), mixed and centrifuged at 10 000 *g* for 30 min. The cell suspension was filtered through a Schleicher & Schuell 595½ paper filter, the effluent was centrifuged for 10 min at 10 000 *g* and resuspended in growth medium. Cells were concentrated to 1 ml in growth medium. The sample was added on top of the gradient, and centrifuged at 6000 *g* for 1 h at 4°C. The lower and upper band in the gradient were transferred to a 50 ml PE tube and centrifuged at 2500 *g* for 15 min at 4°C. The fraction with anammox bacteria that hybridized with BS820 was directly used for DNA extraction or frozen at −80°C. Genomic DNA was isolated according to Zhou and colleagues (1996). This DNA was subjected to 454GS and 454GSFlx pyrosequencing.

For metagenomic DNA, 10 ml of cells were collected directly from the 2 l bioreactor, and DNA was isolated according to the DOE-JGI standard operating procedure (Mavromatis *et al*., 2009). The DNA was subjected to 454Titanium sequencing and Sanger paired end sequencing on shot gun and fosmid libraries with about 3 and 40 kb inserts.

### Annotation

The assembly of DNA sequences from density gradient purified cells was taken as starting point. The reads were assembled with Newbler (454 Life Sciences, Roche Diagnostics) and CLCBio software using minimum length overlap of 50% and a minimum identity of 80%. Initial cut-off for contigs was set at 400 nt. The contig sequences were imported into Artemis (Rutherford *et al*., 2000) and open reading frames (ORFs) were selected that code for more than 100 amino acids. Smaller genes were additionally identified by Glimmer (Delcher *et al*., 1999). blast (http://blast.ncbi.nlm.nih.gov) searches were run against the NCBI non-redundant (NR) and coding domains (CDD) databases, and against a local *K. stuttgartiensis* database (Strous *et al*., 2006). Start codon locations were determined with the aid of Glimmer (Salzberg *et al*., 1998), in combination with manual comparison with the blast search results. Data obtained by additional metagenome sequencing and *de novo* assemblies with different programs and parameters, and automated annotated with RAST (Aziz *et al*., 2008), were used to confirm gene sequences and lengths. Metagenome data were annotated in IMG/G of DOE-JGI. Mapping and *de novo* re-assembly were done with CLC genomics workbench (CLC Bio, Aarhus, Denmark). blast was used for comparison of sequences between *S. profunda* and *K. stuttgartiensis* and for annotation. CDD (http://www.ncbi.nlm.nih.gov/cdd) and Rfam (rfam.sanger.ac.uk) were used for domain search and RNA sequences respectively. KEGG (http://www.genome.jp/kegg/genome) and Metacyc (http://metacyc.org) were used for analysis of metabolic pathways. Signal peptides were predicted with SignalP (Bendtsen *et al*., 2004), transmembrane helices with TMHMM (Sonnhammer *et al*., 1998). Alignments of proteins were done with ProbCons (Do *et al*., 2005), clustalw (Thompson *et al*., 1994) and Muscle (Edgar, 2004). Emboss (http://emboss.sourceforge.net) and CLC Genomics Workbench were used for general sequence analysis. *S. profunda* contigs were aligned to fosmids obtained from the Peruvian Sea, containing *Scalindua* rRNA (mey3 and mey4) or functional (pc46a10 and pc60g12) genes. Alignment was done with Mauve, using the ‘order contigs’ tool with standard settings plus use of seed families and iterative refinement (Darling *et al*., 2004). Additionally, *S. profunda* contigs were aligned with the genome of K. stuttgartiensis (Strous *et al*., 2006) with Mauve using the same settings. Microsoft Excel, Notepad++ (http://notepad-plus-plus.org) and Perl (http://www.perl.org) were used for overview, search, processing and cross-reference analyses.

### Protein and proteome sample preparation and analysis

As a reference for proteome analysis, the translated gene sequences of predicted genes in the genome assembly obtained with density gradient purified cells was used. Crude cell extract was prepared by French press. Metaproteomics analysis was performed twice. For the first preliminary run the proteins of the crude cell extract were separated on a conventional 10% SDS-PAGE gel. Then the gel was cut into four slices for digestion by trypsin. The resulting peptides were identified with liquid chromatography online tandem mass spectrometry (LC-MS/MS) after size fractionation. The second run was performed directly with the crude cell extract, with liquid chromatography online tandem mass spectrometry (Kartal *et al*., 2011; Wessels *et al*., 2011).

The putative HZS enzyme was purified and size fractionated according Kartal and colleagues (2011). Genome information of the *hzs* gene cluster of *S. profunda* and *K. stuttgartiensis* was compared. For each gene the molecular mass of the predicted signal peptide was subtracted from the total molecular mass of the predicted protein. Haem groups (0.6 kDa each) were included in the calculation of total masses. The resulting molecular masses were compared with SDS-PAGE gels of total protein extracts. In addition, bands were cut out from the gel and subjected to MALDI-TOF analysis.

### Transcriptome

*Scalindua profunda* biomass (10 ml) of the 2 l bioreactor operated under nitrite limitation and surplus ammonium at 15°C using the red sea salt medium was used for mRNA extraction. Total RNA isolation was done according to the supplier's instructions, using the RiboPureTM-Bacteria kit (Ambion, Austin, USA). DNAse treatment was performed twice. Reverse transcription was performed using the RevertAidTM First Strand cDNA Synthesis kit (Fermentas GMBH, St. Leon-Rot, Germany) with random hexamer primers according to the supplier's instructions (Kartal *et al*., 2011). Second-strand cDNA synthesis was performed using reagent following the supplier's instructions as described in Kartal and colleagues (2011). At least 20 ng of double-strand cDNA was used for Illumina sequencing. The reads were mapped onto the genome of *S. profunda* using the CLC Genomics Workbench software using a minimum length of 90% and a minimum identity of 90% as described before (Kartal *et al*., 2011). The rRNA genes were excluded from the mapping. The mapped reads were subsequently extracted and checked with blastx to the corresponding amino acid sequence of the proteins to remove false positives.

### Phylogenetic analysis

16S rRNA gene matching genomic reads, collected from genomic DNA of the bioreactor and from density gradient purified cells, were filtered from the pools of reads by mapping against a selection of 16S rRNA genes from the RDP database [lengths > 1200 (Maidak *et al*., 2000)]. The reference genes selection consisted of all type strains, of prokaryotes that were associated with anammox processes and of sequences that were found in oxygen minimum zones, i.e. 7980 sequences in total. Subsequent blastn runs of the filtered sequences against the reference genes were processed with Megan (Huson *et al*., 2007). The arb package was used to accurately determine the phylogenetic position of assembled rRNA sequences longer than 1200 bp (Ludwig *et al*., 2004).

### Fosmid Peruvian OMZ

The marine samples used in this study were collected from the Peruvian OMZ during an expedition of the IMARPE R/V Jose' Olaya off the coast of Peru in April 2005 (Hamersley *et al*., 2007). Biomass form Peruvian OMZ (2000 l) was filtered and used for DNA extraction. The DNA was used to construct a fosmid library (17.000 clones of average 37 kb) as described by Woebken and colleagues (2007). The fosmid library was screened for anammox 16S rRNA genes using an optimized PCR protocol with anammox-specific primer sets (Woebken *et al*., 2007). Two fosmids containing a marine anammox 16S rRNA gene were found. These two fosmids (mey3 and mey4) were selected for full sequencing (see Fig. S3A and B). A further 1000 fosmids were end sequenced, and the sequences were compared with the *S. profunda* and *K. stuttgartiensis* genome assemblies. In this way two more fosmids were retrieved and fully sequenced (Fig. S3C and D).

## Supporting information

**Fig. S1.** MAUVE alignment of‘*Candidatus* Scalindua profunda’ contigs to the‘*Candidatus* Kuenenia stuttgartiensis’ genome assembly.

**Fig. S2.** SDS-PAGE of *Kueneniastuttgartiensis* and *Scalindua profunda* cell extracts.Most proteins that yield visible bands are involved in the anammoxprocess. Three subunits of HZS in *K. stuttgartiensis* areclearly visible, as is the case for *S. profunda*&apos;stwo subunits, HZS-α and the fusion protein HZS-βγ (see also Fig. 3). The identity of bands was confirmed with MALDI-TOF analysis.

**Fig. S3.** MAUVE alignment of *Scalinduaprofunda* contigs to the fosmids retrieved from the Peru OxygenMinimum Zones. (A) Fosmid mey3; (B) fosmid mey4, both contain the*Scalindua* ribosomal RNA operons; (C) fosmid PG46A10; and (D) fosmid PC60G12.

**Fig. S4.** Pie chart of 16S rRNA diversity in theenrichment culture of ‘*Candidatus* Scalinduaprofunda’.

**Table S1.** Description of‘*Candidatus* Scalindua profunda’.

**Table S2.** Fasta file of the bases of the genesof *Scalindua profunda*.

**Table S3.** Fasta file of the amino acids encodedby the genes of *Scalindua profunda*.

**Table S4.** Full transcriptome of *Scalinduaprofunda*.

**Table S5.** Full proteome of *Scalinduaprofunda*.

**Table S6.** Highly expressed *Scalinduaprofunda* genes not found in *K. stuttgartiensis*.

**Table S7.** Overview and distribution ofcytochrome *c*-encoding genes in *Scalinduaprofunda*.

**Table S8.** SNP analysis of octahaem encodingORFs in the *S. profunda* genome assembly.
