# Supplementary figures and images for: The metagenome of the marine anammox bacterium ‘Candidatus Scalindua profunda’ illustrates the versatility of this globally important nitrogen cycle bacterium
Source: Environ Microbiol. 2013 May;15(5):1275–89. doi: 10.1111/j.1462-2920.2012.02774.x (PMC3655542; doi:10.1111/j.1462-2920.2012.02774.x)

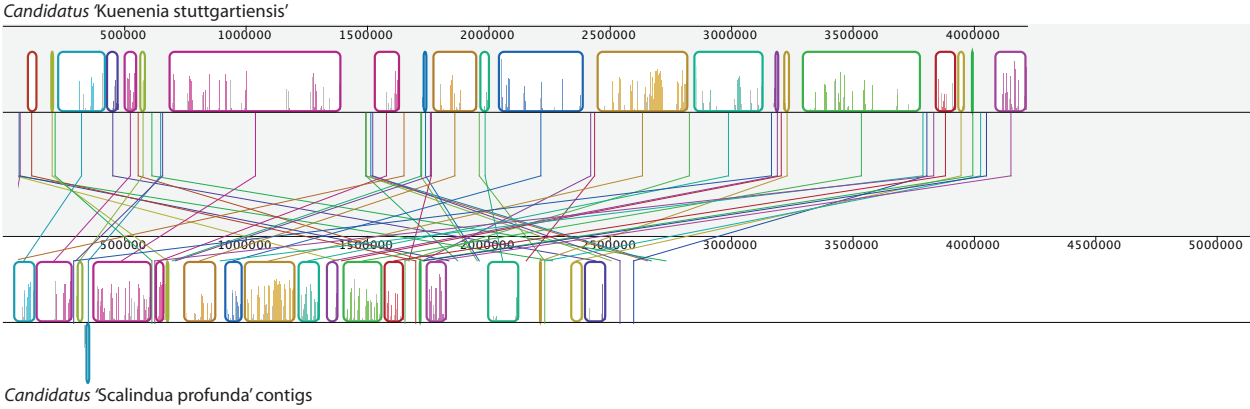

Supplement: Supplementary file 1 [file emi0015-1275-SD1.pdf]

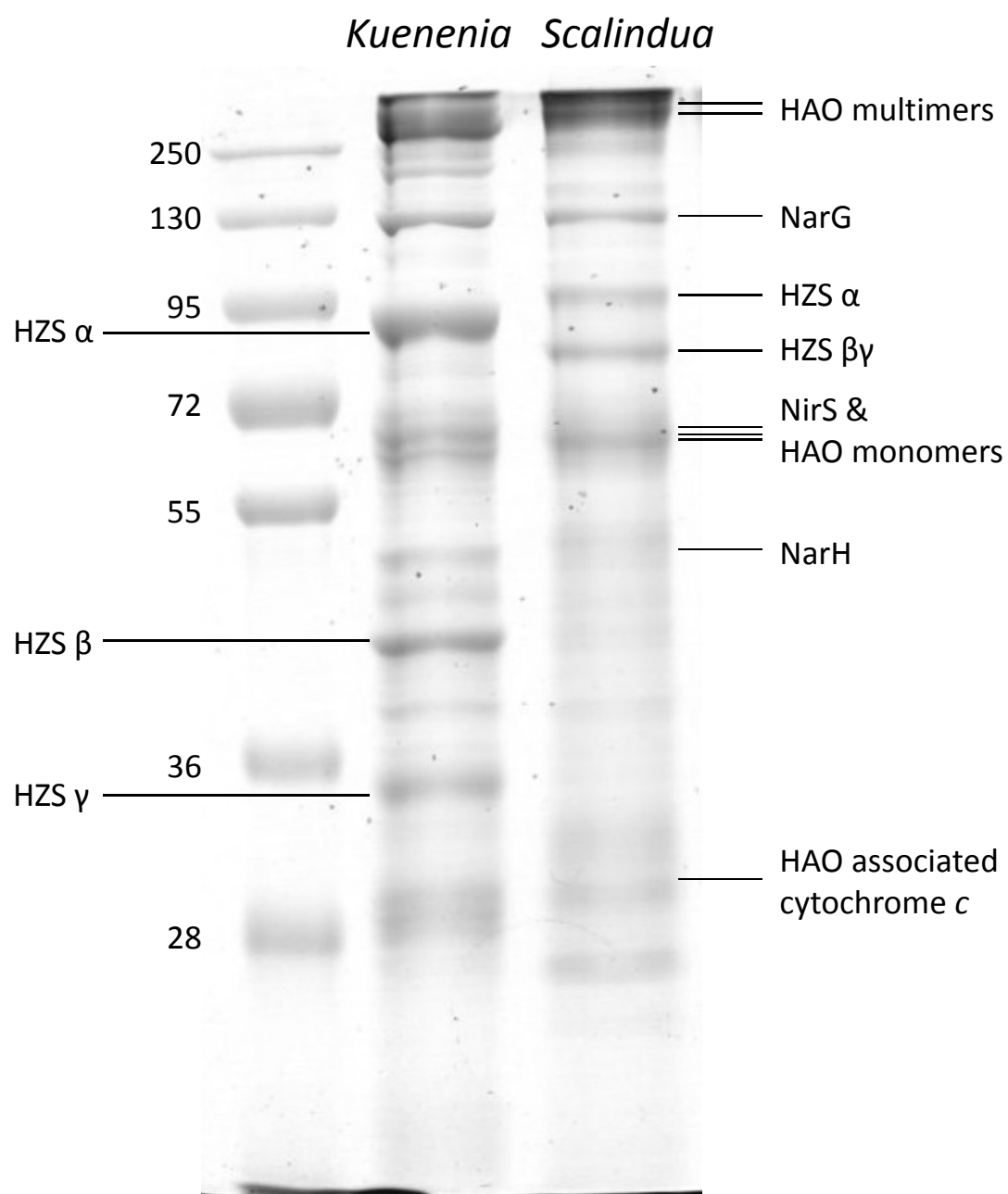

Supplement Fig S1  
Van de Vossenberg et al.

Supplement: Supplementary file 2 [file emi0015-1275-SD2.pdf]

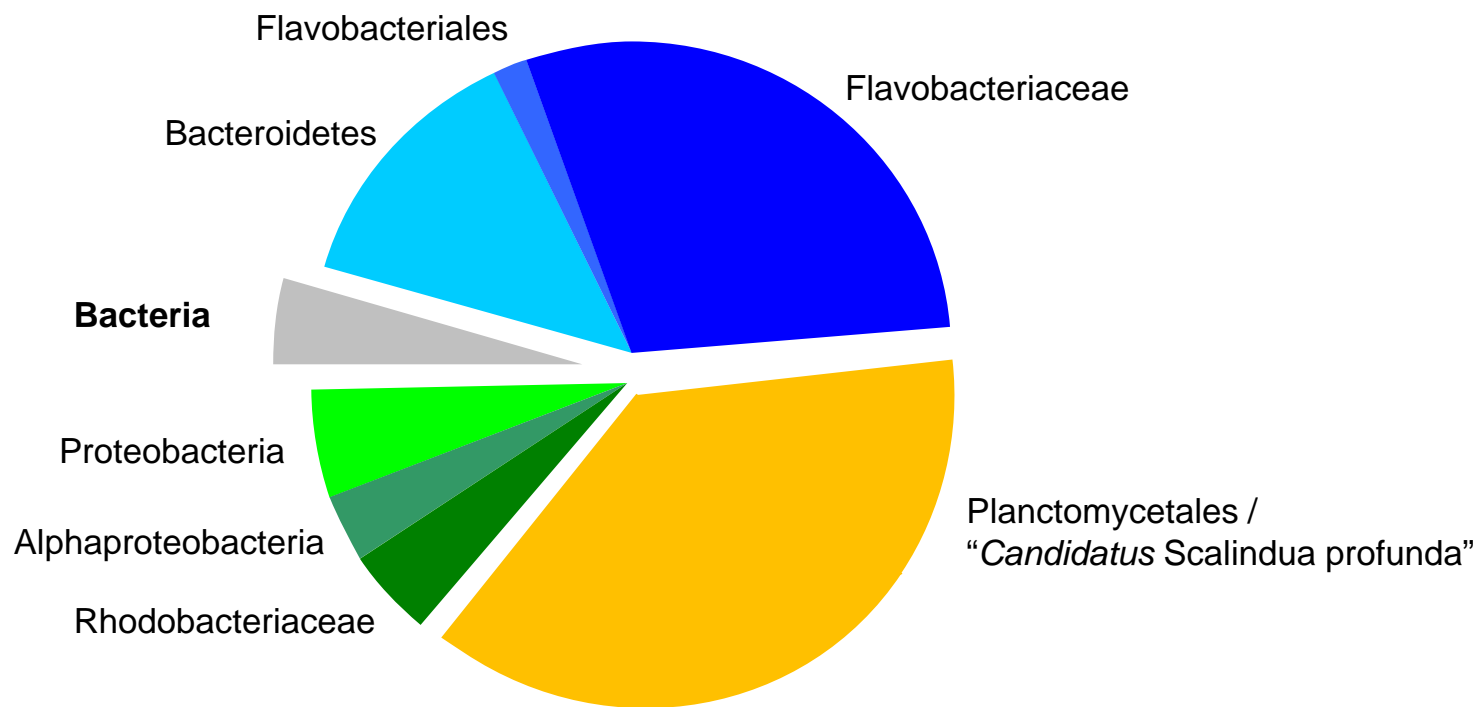

Supplement Fig 4 Van de Vossenberg et al.

Supplement: Supplementary file 7 [file emi0015-1275-SD7.pdf]
