# Supplementary material for: The metagenome of the marine anammox bacterium ‘Candidatus Scalindua profunda’ illustrates the versatility of this globally important nitrogen cycle bacterium
Source: Environ Microbiol. 2013 May;15(5):1275–89. doi: 10.1111/j.1462-2920.2012.02774.x (PMC3655542; doi:10.1111/j.1462-2920.2012.02774.x)
Supplement: Supplementary file 3 [file emi0015-1275-SD3.pdf]

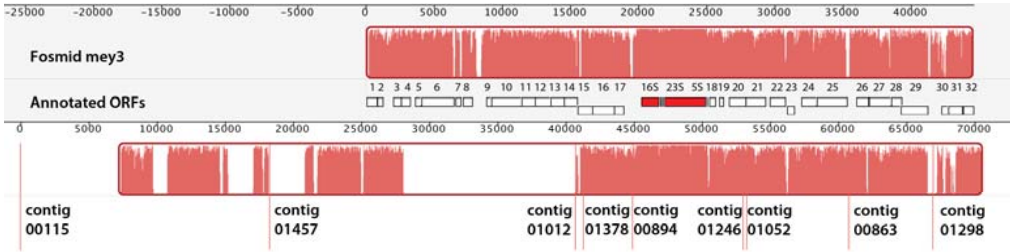


| Query | Lowest E-value | Description (E-value) | Greatest identity % |
|---|---|---|---|
| mey3_001 | 8,64E-144 | scal00791c putative DNA-directed DNA polymerase | 95 |
| mey3_002 | 2,23E-50 | scal00790c putative sugar epimerase | 83 |
| mey3_003 | 2,32E-79 | scal00789c ABC-type transport protein | 90 |
| mey3_004 | 2,57E-106 | scal00788c putative ABC transport protein | 85 |
| mey3_005 | 6,22E-68 | scal00787c hypothetical protein | 88 |
| mey3_006 | 0 | scal00786c V-type H(+)-translocating pyrophosphatase | 93 |
| mey3_007 | 1,06E-54 | scal00785c putative iojap-like protein | 88 |
| mey3_008 | 3,32E-69 | scal00780c putative Paraquat-inducible protein A pqiA | 95 |
| mey3_009 | 1,29E-61 | scal03997 PilT like protein | 93 |
| mey3_010 | 0 | scal03998 oligopeptide ABC transport protein | 92 |
| mey3_011 | 2,33E-175 | scal03999 oligopeptide ABC transport protein | 97 |
| mey3_012 | 0 | scal04000 oligopeptide ABC transport protein | 91 |
| mey3_013 | 2,55E-166 | scal04001 oligopeptide ABC transport protein | 93 |
| mey3_014 | 7,22E-171 | scal04002 Oligopeptide/dipeptide ABC transport protein | 91 |
| mey3_015 | 7,17E-150 | scal03849c DNA topoisomerase VI, subunit A | 97 |
| mey3_016 | 0 | scal03850c DNA topoisomerase VI, B subunit | 94 |
| mey3_017 | 6,07E-83 | scal03851c zinc metalloprotease | 83 |
| 16S rRNA | 0 | 16S rRNA | 98 |
| tRNA ala | 2,00E-39 | tRNA ala | 99 |
| tRNA ile | 2,00E-44 | tRNA ile | 99 |
| 23S rRNA | 0 | 23S rRNA | 98 |
| 5S rRNA | 2,49E-64 | 5S rRNA | 99 |
| mey3_018 | 5,88E-65 | scal02422 ferric uptake regulator protein | 92 |
| mey3_019 | 3,14E-44 | scal02423 glutamyl-tRNA(Gln) amidotransferase subunit C | 88 |
| mey3_020 | 1,49E-174 | scal02424 glutamyl-tRNA(Gln) amidotransferase subunit A | 93 |
| mey3_021 | 0 | scal02852 glutamyl-tRNA(Gln) amidotransferase, B subunit | 94 |
| mey3_022 | 0 | scal02853 glutamate 5-kinase proB | 93 |
| mey3_023 | 1,55E-91 | scal02854c anthranilate synthase component II pabA | 93 |
| mey3_024 | 4,55E-168 | scal02855 transcription elongation protein NusA | 95 |
| mey3_025 | 0 | scal02856 translation initiation factor IF-2 | 92 |
| mey3_026 | 5,59E-162 | orf03865 ATP phosphoribosyltransferase | 94 |
| mey3_027 | 0 | scal02151 putative TPR repeat protein | 82 |
| mey3_028 | 2,68E-131 | scal02152 hypothetical COG0217 protein | 91 |
| mey3_029 | 0 | scal02153c cell division protein FtsH | 93 |
| mey3_030 | 1,594 | scal03898c conserved hypothetical protein | 41 |
| mey3_031 | 0 | scal03645c NAD(P)H glycerol-3-phosphate dehydrogenase G[illegible] | 90 |
| mey3_032 | 5,97E-137 | scal03646c serine hydroxymethyl transferase | 91 |
