# Supplementary material for: The metagenome of the marine anammox bacterium ‘Candidatus Scalindua profunda’ illustrates the versatility of this globally important nitrogen cycle bacterium
Source: Environ Microbiol. 2013 May;15(5):1275–89. doi: 10.1111/j.1462-2920.2012.02774.x (PMC3655542; doi:10.1111/j.1462-2920.2012.02774.x)
Supplement: Supplementary file 4 [file emi0015-1275-SD4.pdf]

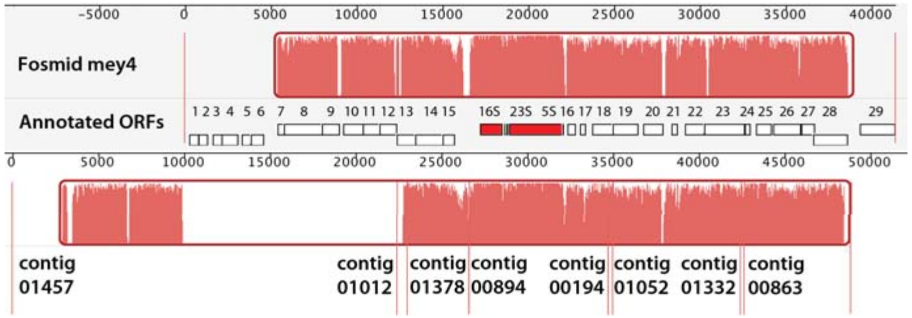

Query	Lowest E-value	Description (E-value)	Greatest identity %
mey4_001c	1,509	scal01668 hypothetical protein	36
mey4_002c	2,058	kuste3957 hypothetical protein	52
mey4_003c	9,988	kustd1309 conserved hypothetical protein	36
mey4_004c	0,12	kustd1355 conserved hypothetical protein	48
mey4_005c	2,887	scal00729c dihydropicolinate synthase	56
mey4_006c	0,016	kuste2561 hypothetical protein	44
mey4_007	3,34E-59	scal03997 PilT like protein	90
mey4_008	0	scal03998 oligopeptide ABC transport protein	92
mey4_009	2,75E-170	scal03999 oligopeptide ABC transport protein	99
mey4_010	0	scal04000 oligopeptide ABC transport protein	92
mey4_011	7,79E-167	scal04001 oligopeptide ABC transport protein	96
mey4_012	1,39E-174	scal04002 Oligopeptide/dipeptide ABC transport protein	93
mey4_013c	2,52E-152	scal03849c DNA topoisomerase VI, subunit A	98
mey4_014c	0	scal03850c DNA topoisomerase VI, B subunit	95
mey4_015c	5,58E-83	scal03851c zinc metalloprotease	84
16S rRNA	0	16S rRNA	97
tRNA ala	2,00E-39	tRNA ala	99
tRNA ile	2,00E-44	tRNA ile	99
23S rRNA	0	23S rRNA	97
5S rRNA	2,49E-64	5S rRNA	99
mey4_016	1,49E-62	scal02422 ferric uptake regulator protein	91
mey4_017	2,71E-44	scal02423 glutamyl-tRNA(Gln) amidotransferase subunit C	88
mey4_018	1,27E-165	scal02424 glutamyl-tRNA(Gln) amidotransferase subunit A	92
mey4_019	0	scal02852 glutamyl-tRNA(Gln) amidotransferase, B subunit	100
mey4_020	9,65E-156	scal02853 glutamate 5-kinase proB	88
mey4_021	0,226	kustc1161 unknown protein	73
mey4_022	7,79E-165	scal02855 transcription elongation protein NusA	100
mey4_023	0	scal02856 translation initiation factor IF-2	85
mey4_024	1,78E-25	kuste3088 conserved hypothetical protein	53
mey4_025	2,84E-164	orf03865 ATP phosphoribosyltransferase	96
mey4_026	0	scal02151 putative TPR repeat protein	80
mey4_027	6,28E-133	scal02152 hypothetical protein	92
mey4_028c	0	scal02153c cell division protein FtsH	92
mey4_029	0,388	kuste4427 hypothetical protein	59
