# Supplementary material for: The metagenome of the marine anammox bacterium ‘Candidatus Scalindua profunda’ illustrates the versatility of this globally important nitrogen cycle bacterium
Source: Environ Microbiol. 2013 May;15(5):1275–89. doi: 10.1111/j.1462-2920.2012.02774.x (PMC3655542; doi:10.1111/j.1462-2920.2012.02774.x)
Supplement: Supplementary file 5 [file emi0015-1275-SD5.pdf]

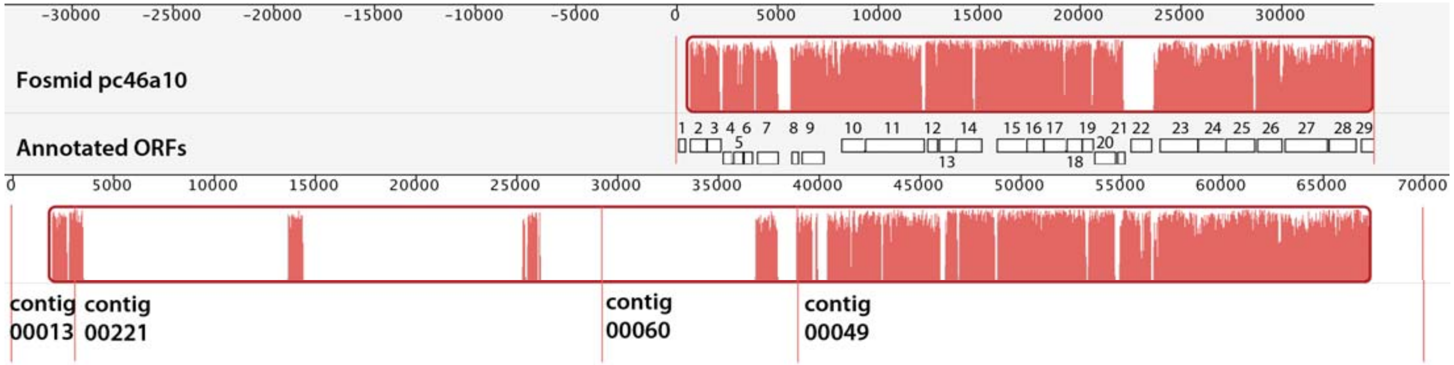

Query	Lowest E-value	Description (E-value)	Greatest identity %
PC46A10_001	0,229	scal01996 hypothetical protein	48
PC46A10_002	6,15E-135	scal00073 unknown protein	85
PC46A10_003	1,18E-78	scal01514 hypothetical protein	87
PC46A10_004c	1,50E-63	scal01522c cation efflux protein CzcA	83
PC46A10_005c	1,82E-35	scal01522c cation efflux protein CzcA	83
PC46A10_006c	1,70E-64	scal01529c conserved hypothetical protein	88
PC46A10_007c	1,01E-126	scal00470c hypothetical protein	74
PC46A10_008c	6,17E-37	scal00368 competence protein ComA	79
PC46A10_009c	4,73E-89	scal00368 competence protein ComA	75
PC46A10_010	0	scal00367c pleiotrophic regulatory protein DegT	87
PC46A10_011	0	scal00366c unknown protein	79
PC46A10_012	8,50E-87	scal00365c phosphopantetheine adenylyltransferase (PPAT)	95
PC46A10_013	6,29E-148	scal00364c conserved hypothetical protein	95
PC46A10_014	0	scal00363c exodeoxyribonuclease VII	91
PC46A10_015	0	scal00362c 1-deoxy-D-xylulose 5-phosphate synthase	90
PC46A10_016	3,86E-140	scal00361c inorganic polyphosphate	89
PC46A10_017	0	scal00360c porA pyruvate:ferredoxin oxidoreductases, alpha subunit	91
PC46A10_018	3,44E-136	scal00359c porB pyruvate:ferredoxin oxidoreductases, beta subunit	90
PC46A10_019	9,25E-89	scal00358c porC pyruvate:ferredoxin oxidoreductases, gamma subunit	96
PC46A10_020c	1,63E-159	scal00357 fibronectin type III domain containing protein	85
PC46A10_021c	1,44E-48	scal00356 hypothetical protein	93
PC46A10_022	5,56E-04	kustd1479 fba similar to fructose-biphosphate aldolase	38
PC46A10_023	0	scal00354c sucrose phosphorylase	87
PC46A10_024	0	scal00353c diphosphate--fructose-6-phosphate 1-phosphotransferase	80
PC46A10_025	0	scal00351c Pyruvate kinase	88
PC46A10_026	0	scal00350c acetate kinase	88
PC46A10_027	0	scal00349c acetyl-coa synthetase ADP forming	81
PC46A10_028	0	scal00347c conserved hypothetical protein	85
PC46A10_029	3,34E-121	scal00346c threonine synthase thrC	93
