# Supplementary material for: The metagenome of the marine anammox bacterium ‘Candidatus Scalindua profunda’ illustrates the versatility of this globally important nitrogen cycle bacterium
Source: Environ Microbiol. 2013 May;15(5):1275–89. doi: 10.1111/j.1462-2920.2012.02774.x (PMC3655542; doi:10.1111/j.1462-2920.2012.02774.x)
Supplement: Supplementary file 6 [file emi0015-1275-SD6.pdf]

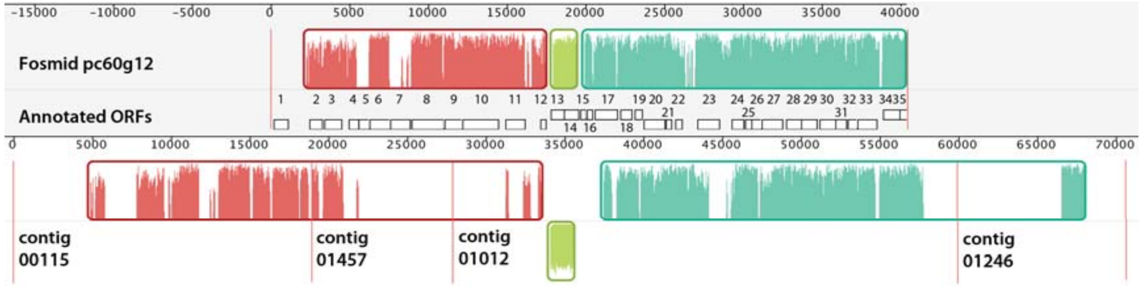


| Query | Lowest E-valu | Description (E-value) | Greatest identity % |
|---|---|---|---|
| PC60G12_001c | 3,08E-46 | scal00301 unknown protein | 58 |
| PC60G12_002c | 6,46E-72 | scal00151 multiheme protein | 78 |
| PC60G12_003c | 3,80E-118 | scal00149 putative multiheme protein | 62 |
| PC60G12_004c | 1,28E-65 | scal00147 putative cytochrome b | 60 |
| PC60G12_005c | 1,33 | scal01898 ATP-binding protein | 34 |
| PC60G12_006c | 0 | scal00146 aminotransferase class III gabT | 91 |
| PC60G12_007c | 0,076 | scal02193 TPR repeat protein | 52 |
| PC60G12_008c | 0 | scal00144 putative peptidase U32 | 91 |
| PC60G12_009c | 0 | scal00143 conserved hypothetical protein | 80 |
| PC60G12_010c | 0 | scal00142 hypothetical protein | 81 |
| PC60G12_011c | 0 | scal04058 hypothetical protein | 75 |
| PC60G12_012c | 7,46E-23 | scal00599 putative phosphoserine phosphatase | 69 |
| PC60G12_013 | 2,91E-127 | scal00597 serine/threonine protein kinase | 81 |
| PC60G12_014 | 1,22E-149 | scal00598 putative phosphoserine phosphatase | 86 |
| PC60G12_015 | 5,46E-45 | scal00596c amtB ammonium transport protein | 85 |
| PC60G12_016 | 1,76E-59 | scal00595c nitrogen regulatory protein P-II GlnK | 99 |
| PC60G12_017 | 0 | scal00594c amtB ammonium transport protein | 86 |
| PC60G12_018 | 3,33E-113 | scal00593c putative serine/threonine protein kinase | 82 |
| PC60G12_019 | 2,58E-47 | scal00592c unknown protein | 64 |
| PC60G12_020c | 0 | scal00591 amtB ammonium transport protein | 91 |
| PC60G12_021c | 1,78E-48 | scal00590 GlnK nitrogen regulatory protein P-II | 94 |
| PC60G12_022c | 5,21E-28 | scal00589 unknown protein | 53 |
| PC60G12_023c | 0 | scal00587 amtB ammonium transport protein | 94 |
| PC60G12_024c | 6,85E-106 | scal00586 putative MazG like pyrophosphatase | 90 |
| PC60G12_025c | 3,81E-61 | scal00585 biopolymer transport ExbD protein | 95 |
| PC60G12_026c | 1,85E-95 | scal00584 putative ExbB biopolymer transport protein | 88 |
| PC60G12_027c | 1,33E-135 | scal00583 unknown protein | 70 |
| PC60G12_028c | 3,10E-138 | scal00582 putative membrane protease | 83 |
| PC60G12_029c | 1,79E-160 | scal00581 putative membrane protease | 90 |
| PC60G12_030c | 4,29E-161 | scal00580 hydrogenase maturation protein | 86 |
| PC60G12_031c | 3,55E-125 | scal00579 conserved hypothetical protein | 88 |
| PC60G12_032c | 5,27E-94 | scal00578 hypothetical protein | 84 |
| PC60G12_033c | 0 | scal00577 hypothetical protein | 90 |
| PC60G12_034 | 0 | scal03910 4-hydroxy-3-methylbut-2-en-1-yl diphosphate synthase | 93 |
| PC60G12_035 | 3,86E-77 | scal03911 putative 1-deoxy-D-xylulose-5-phosphate reductoisomerase | 89 |
