# Supplementary material for: The metagenome of the marine anammox bacterium ‘Candidatus Scalindua profunda’ illustrates the versatility of this globally important nitrogen cycle bacterium
Source: Environ Microbiol. 2013 May;15(5):1275–89. doi: 10.1111/j.1462-2920.2012.02774.x (PMC3655542; doi:10.1111/j.1462-2920.2012.02774.x)
Supplement: Supplementary file 8 [file emi0015-1275-SD8.pdf]

Description of ‘Candidatus Scalindua profunda’ sp. nov.

Etymology. Sca.lin.du.a made of ladder molecules (Kuypers et al 2003; Schmid et al 2003; Jetten et al 2010) pro.fun.da L.n. sediment . The name indicates that these Scalindua cells were originally enriched from a sediment of the Alsbeck Fjord Sweden (van de Vossenberg et al 2008)

Properties. Ammonium-oxidizing and nitrite-reducing anammox bacterium of the order *Brocadiales*. Grows anaerobically, and reduces nitrite to dinitrogen gas with hydrazine as intermediate. Gram-negative, non-motile coccoid cells, with a diameter of 0.5 - 0.8 µm. Low temperature (enriched at 15-18 °C) and neutrophilic (pH 7-8). Slow growth (doubling time 2 weeks). Cells contain prokaryotic compartment , the anammoxosome, that is assumed to be the locus of anammox catabolism. Cells contain special ladderane lipids (Rattray et al 2008).

Jetten MSM, Op den Camp HJM, Kuenen JG and Strous M (2010) Description of the order Brocadiales. Bergey’s Manual of Systematic Bacteriology Vol 4 pp 596-603.

Kuypers MMM, Sliekers AO, Lavik G, Schmid M, Jorgensen BB, Kuenen JG, Damste JSS, Strous M and Jetten MSM (2003) Anaerobic ammonium oxidation by anammox bacteria in the Black Sea. Nature 422(6932): 608-611.

Rattray JE, van de Vossenberg J, Hopmans EC, Kartal B, van Niftrik L, Rijpstra WIC, Strous M, Jetten MSM, Schouten S and Damste JSS (2008) Ladderane lipid distribution in four genera of anammox bacteria. Archives of Microbiology 190(1): 51-66.

Schmid M, Walsh K, Webb R, Rijpstra WIC, van de Pas-Schoonen K, Verbruggen MJ, Hill T, Moffett B, Fuerst J, Schouten S, Damste JSS, Harris J, Shaw P, Jetten MSM and Strous M (2003) Candidatus "Scalindua brodae", sp. nov., Candidatus "Scalindua wagneri", sp. nov., two new species of anaerobic ammonium oxidizing bacteria. Systematic and Applied Microbiology 26(4): 529-538.

van de Vossenberg J, Rattray JE, Geerts W, Kartal B, van Niftrik L, van Donselaar EG, Damste JSS, Strous M and Jetten MSM (2008) Enrichment and characterization of marine anammox bacteria associated with global nitrogen gas production. Environmental Microbiology 10(11): 3120-3129. 141.
