# Supplementary material for: The metagenome of the marine anammox bacterium ‘Candidatus Scalindua profunda’ illustrates the versatility of this globally important nitrogen cycle bacterium
Source: Environ Microbiol. 2013 May;15(5):1275–89. doi: 10.1111/j.1462-2920.2012.02774.x (PMC3655542; doi:10.1111/j.1462-2920.2012.02774.x)
Supplement: Supplementary file 11 [file emi0015-1275-SD11.pdf]

Name	Rank coverage	Number of reads	Relative coverage versus average	Annotation
scal00998	1	7648	143,83	ISxac3 transposase
scal00997	2	14466	84,66	strongly similar to translational elongation factor Tu
scal00025	3	19176	68,16	Hydrazine synthase beta-gamma
scal02632	4	4688	47,44	hypothetical (triheme) protein of hydrazine synthase cluster with 3 cxxch and 1 cxch mottive
scal00216	5	3315	40,02	sigma-70 factor
scal03295	6	8341	37,17	CxxxxCH containing hydrazine oxidase (hzo)
scal04280	7	1728	27,18	unknown protein
scal01317	8	5662	25,03	hydroxylamine oxidoreductase hao
scal01701	9	1286	23,62	hydroxylamine oxidoreductase hao
orf01332	10	739	23,23	No hits found
scal01318	11	8062	22,82	hydrazine synthase alpha, diheme protein
scal04254	12	1471	21,95	similar to low molecular weight heat shock protein (Hsp17)
orf07369	13	891	21,88	No hits found
scal02635	14	3863	21,47	sigma 54 response regulator of hydrazine synthase cluster
scal02098	15	5239	20,56	nirS cd1 nitrite reductase
orf05273	16	595	19,90	hypothetical protein
scal02863	17	1034	19,14	heavy metal translocating P-type ATPase
scal02633	18	2453	18,07	putative multiheme protein with 6 cxxch motives and 1 cxxxch motive of hydrazine synthase cluster
scal02277	19	840	17,62	30S ribosomal protein S12
scal02167	20	1393	16,98	50S ribosomal protein L25
scal02111	21	3637	14,54	putative cell wall associated protein
scal00343	22	675	13,94	expressed hypothetical cysteine rich protein
scal01488	23	1291	13,81	TENA/PQQ biosynthesis protein C involved in enhancing extracellular expression
scal02059	24	1776	13,60	asrC anaerobic sulfite reductase subunit C
scal04116	25	767	13,58	expressed hypotheticial monoheme protein
scal04328	26	375	13,30	partial cold shock protein CspB
scal02280	27	729	12,46	50S ribosomal protein L7/L12
scal02186	28	1104	12,12	unknown protein
scal00864	29	1715	12,07	unknown protein
scal02099	30	1984	11,96	SAM radical nirJ like heme d synthesis protein
orf02709	31	377	11,91	No hits found
scal01015	32	687	11,75	50S ribosomal protein L18
scal01030	33	983	11,66	50S ribosomal protein L3
scal04115	34	609	11,41	putative monoheme protein cytochrome c553
scal00717	35	550	11,25	unknown protein
scal04138	36	697	11,00	No hits found
scal02989	37	985	10,75	conserved hypothetical protein
orf05272	38	320	10,63	predicted protein
scal01029	39	1011	10,56	hypothetical protein MYPU_5880
scal02166	40	1328	10,29	phosphoribosylpyrophosphate synthetase
scal02130	41	529	10,22	putative iron sulfur cofactor assembly protein
scal04164	42	1529	9,95	hydroxylamine oxidoreductase hao
scal00867	43	1827	9,49	nitrate reductase subunit NarH
orf05149	44	486	9,26	No hits found

scal02114	45	1814	9,26	CO dehydrogenase/acetyl-coA synthase beta subunit (acsA)partial
scal00420	46	1507	8,94	expressed conserved hypothetical diheme cytochrome protein associated with hao
scal01004	47	670	8,76	50S ribosomal protein L17
scal04322	48	319	8,58	DNA-binding protein HU-beta
scal02052	49	1242	8,47	putative multiheme cytochrome c protein with 5 cxxch motives and 1 cxxxxxch motive
scal00581	50	1156	8,44	expressed putative membrane protease
scal02588	51	376	8,42	putative copper-transporting P-type ATPase
scal02990	52	442	8,21	unknown protein
scal00421	53	2010	8,03	expressed hydroxylamine oxidoreductase hao cluster 4
scal02110	54	1812	8,01	putative hydroxylamine oxidoreductase hao with 8 heme / cxxch motives
scal00170	55	991	7,87	expressed PKD domain protein
scal01130	56	1522	7,76	conserved hypothetical protein
scal01013	57	430	7,53	50S ribosomal protein L15
scal03929	58	1297	7,46	putative ATP/GTP binding protein
scal02279	59	3632	7,37	DNA-directed RNA polymerase, beta subunit
scal00217	60	847	7,03	hypothetical protein
scal01624	61	272	6,91	conserved hypothetical protein
scal02058	62	440	6,91	conserved hypothetical COG2210 protein
scal02285	63	425	6,75	similar to preprotein translocase SecE subunit
scal03225	64	547	6,75	No hits found
scal00952	65	359	6,50	putative superoxide reductase
scal03974	66	346	6,46	hydroxylamine oxidoreductase hao
scal00415	67	380	6,39	expressed putative signal-transduction protein with CBS domains
scal00868	68	860	6,39	putative narM like gamma subunit nitrate reductase
orf06112	69	242	6,37	conserved hypothetical protein
scal00567	70	270	6,34	RNA-binding region RNP-1 (RNA recognition motif)
scal02109	71	901	6,30	putative multiheme cytochrome c protein with 6 cxxch motives
scal01162	72	613	6,30	ribonucleoside triphosphate reductase activating protein NrdG
scal01010	73	632	6,21	methionine aminopeptidase
scal02752	74	1349	6,18	heat shock protease DegP/HtrA
scal01129	75	409	6,12	conserved hypothetical protein
scal00582	76	741	5,98	expressed putative membrane protease
scal02822	77	694	5,96	unknown protein
scal02243	78	363	5,82	30S ribosomal protein S9
scal02284	79	454	5,80	transcription antitermination protein NusG
scal00631	80	573	5,66	expressed ompA/motB flagellar motor protein
scal01009	81	281	5,52	30S ribosomal protein S13
scal02578	82	277	5,50	similar to DnaK suppressor protein
scal02146	83	221	5,45	No hits found
scal04315	84	167	5,41	acyl carrier protein AcpP
scal03302	85	225	5,25	conserved hypothetical protein
scal02755	86	243	5,18	conserved hypothetical protein
scal02521	87	1224	5,16	formyltetrahydrofolate synthetase
scal04312	88	279	5,13	partial hydroxylamine oxidoreductase (hao)
scal02374	89	597	5,09	similar to ispE 4-diphosphate cytidyl 2-C methyl D erythritol kinase
scal03169	90	305	5,07	heat shock protein (Hsp17)
scal03825	91	204	5,04	polysialic acid capsule expression protein
scal00673	92	298	5,04	strong similarity to SsrA-binding protein
scal02797	93	191	5,02	chaperonin GroES

scal02237	94	663	5,02	conserved hypothetical protein of nirHD gene cluster involved in heme d1 biosynthesis
scal01623	95	325	4,91	unknown protein
scal00595	96	233	4,91	expressed nitrogen regulatory protein P-II
scal01084	97	245	4,91	unknown protein
scal03754	98	257	4,86	unknown protein
orf06783	99	163	4,81	conserved hypothetical protein
orf01444	100	523	4,59	'=scal00865c, add numbers accordingly
scal02404	101	1614	4,56	strongly similar to alanyl-tRNA synthetase
scal03486	102	771	4,54	strongly similar to sigma 54 response regulatory protein
scal00866	103	380	4,50	unknown protein
scal02275	104	1249	4,50	translation elongation factor EF-G
scal02524	105	444	4,40	unknown protein of narGH cluster
scal03438	106	471	4,36	Myo-inositol-1-phosphate synthase
orf00338	107	158	4,36	F0F1 ATP synthase subunit epsilon
scal01646	108	302	4,34	similar to NADH dehydrogenase I subunit J
scal00863	109	2052	4,34	nitrate reductase subunit NarG
scal03024	110	227	4,29	rubredoxin-like superoxide reductase
scal03008	111	235	4,29	unknown protein
scal04175	112	387	4,29	thioredoxin peroxidase
scal00452	113	661	4,27	cytochrome c peroxidase
scal01007	114	245	4,24	30S ribosomal protein S11
scal04258	115	282	4,24	hypothetical protein
scal02281	116	301	4,22	50S ribosomal protein L10
scal03648	117	1170	4,20	threonyl-tRNA synthetase
scal00196	118	954	4,13	expressed F1F0 ATP synthase beta subunit
scal04155	119	180	4,06	conserved hypothetical protein
scal04194	120	369	4,06	cytidylate kinase
scal03083	121	671	4,04	PKD domain protein
scal00419	122	406	4,04	expressed putative PAS/PAC sensor protein
scal02935	123	439	4,04	unknown protein COG3945
scal00865	124	508	3,97	putative narD like protein; = orf01444 add numbers accordingly
scal04329	125	145	3,90	putative tatA/E protein
orf05353	126	78	3,90	No hits found
scal02855	127	644	3,90	transcription elongation protein NusA
scal02055	128	976	3,88	GltD like FAD containing NAD(P) oxidoreductase
scal04230	129	266	3,86	unknown protein
scal02106	130	286	3,81	putative rieske 2Fe-2S protein of bc1 complex
scal02282	131	370	3,79	50S ribosomal protein L1
scal00894	132	111	3,79	No hits found
scal02426	133	638	3,74	putative tpr repeat protein
scal01019	134	166	3,74	50S ribosomal protein L24
scal02108	135	428	3,70	putative cytochrome b protein of bc1 complex
scal01040	136	324	3,70	viral A-type inclusion protein, putative
scal00636	137	141	3,65	chaperonin GroES
scal02405	138	596	3,65	fatty acid/phospholipid synthesis protein PlsX
scal01026	139	402	3,58	50S ribosomal protein L2
scal02010	140	315	3,56	orotate phosphoribosyltransferase
scal00418	141	755	3,51	expressed putative PAS/PAC sensor protein
scal00686	142	1366	3,49	putative multiheme protein containing 8 heme/ cxxch motives

scal03060	143	329	3,49	partial CO dehydrogenase/acetyl-CoA synthase alpha subunit acsB
scal01006	144	325	3,45	30S ribosomal protein S4
scal00670	145	777	3,45	60 kDa chaperonin (groEL protein)
scal03755	146	192	3,45	conserved hypothetical protein
scal02563	147	305	3,42	unknown protein
scal03716	148	167	3,40	putative D-xylulose 5-phosphate/D-fructose 6-phosphate phosphoketolase
scal01012	149	629	3,35	preprotein translocase secY protein N terminus
scal02278	150	1863	3,35	DNA-directed RNA polymerase, beta prime subunit
scal04031	151	128	3,35	No hits found
scal04150	152	138	3,31	50S ribosomal protein L21
scal04034	153	756	3,31	putative ABC type transport protein, auxiliary component
scal01127	154	1093	3,31	strongly similar to negative regulator of genetic competence ClpC/MecB
scal02116	155	657	3,29	hydroxylamine oxidoreductase hao
scal02326	156	417	3,26	unknown protein
scal01020	157	167	3,24	50S ribosomal protein L14
scal04318	158	86	3,24	cold shock protein A
scal02054	159	335	3,22	putative qcrB cytochrome b6 of bc1 complex
scal00315	160	466	3,17	expressed 3-isopropylmalate dehydrogenase leuB
scal00538	161	380	3,17	S-adenosyl-dependent methyltransferase
scal00674	162	168	3,15	similar to two-component response regulator CheY
scal02407	163	504	3,08	3-oxoacyl-[acyl-carrier-protein] synthase II (KASII)
scal02757	164	195	3,08	conserved hypothetical protein
scal02413	165	121	3,06	uncharacterized conserved coiled coil protein
scal04038	166	207	3,03	unknown protein
scal02175	167	341	3,03	UDP-3-O-acyl-N-acetylglucosamine deacetylase
scal02798	168	751	3,03	chaperonin GroEL
scal02793	169	154	3,03	hypothetical protein CAB357
orf03709	170	100	3,03	similar to response regulator sirA
scal04111	171	228	3,01	2-C-methyl-D-erythritol-2,4-cyclodiphosphate synthase
scal02936	172	230	2,99	putative 4Fe-4S ferredoxin, iron-sulfur binding
scal03897	173	636	2,99	strongly similar to inosine-5'-monophosphate dehydrogenase
scal02751	174	504	2,99	similar to iron sulfur
scal02856	175	927	2,99	translation initiation factor IF-2
scal01135	176	423	2,97	Chaperone DnaJ
scal02406	177	418	2,97	3-oxoacyl[acyl-carrier protein] synthase III
scal02371	178	529	2,94	similar to trigger factor (TF)
scal02181	179	214	2,94	ribosome recycling factor
scal02242	180	178	2,94	50S ribosomal protein L13
scal01638	181	210	2,92	strongly similar to proton-translocating NADH dehydrogenase I chain B
scal02634	182	360	2,92	putative cytochrome b protein of hydrazine synthase cluster
scal02814	183	246	2,92	unknown conserved protein
scal00079	184	346	2,92	MORN repeat protein
scal03649	185	174	2,90	translation initiation factor IF-3
scal01014	186	209	2,90	30S ribosomal protein S5
scal01279	187	241	2,88	hypothetical protein TDE1324
scal00651	188	428	2,88	putative transposase
scal01011	189	251	2,85	adenylate kinase
scal01058	190	447	2,85	Alcohol dehydrogenase
scal03194	191	316	2,85	strongly similar to acetyl-CoA carboxylase, beta subunit

scal02267	192	294	2,83	similar to CDP-diacylglycerol-serine O-phosphatidyltransferase
scal00660	193	775	2,83	expressed NADH:ubiquinone oxidoreductase subunit nuoF
scal03676	194	181	2,78	RNA-binding region RNP-1 (RNA recognition motif)
scal03185	195	307	2,78	unknown protein
scal02170	196	158	2,72	single-stranded DNA-binding protein
scal01018	197	216	2,72	50S ribosomal protein L5
scal02107	198	301	2,72	qcrB cytochrome b6
scal03963	199	366	2,72	transposase, IS4
scal00635	200	605	2,72	expressed chaperonin GroEL
orf05944	201	102	2,72	ATP synthase protein 1
scal03545	202	683	2,69	heat shock protein Hsp90
scal01410	203	502	2,67	strongly similar to signal recognition particle protein
scal03057	204	122	2,65	putative plasmid maintenance protein HigB with helix turn helix motiv
orf05351	205	113	2,65	conserved hypothetical protein
scal03787	206	261	2,65	similar to 4-phosphopantotheoylcysteine decarboxylase
scal01566	207	188	2,65	strongly similar to peptide deformylase
scal00881	208	305	2,65	putative S1/S6 peptidase
scal00609	209	160	2,65	extensin, putative
scal03203	210	97	2,62	No hits found
scal01169	211	173	2,62	unknown anammox protein
orf05312	212	93	2,62	similar to response regulator
scal02956	213	164	2,60	similar to (3R)-hydroxymyristoyl acyl carrier protein dehydrase
scal04308	214	110	2,60	50S ribosomal protein L29
scal02754	215	506	2,60	strongly similar to RNA polymerase subunit sigma-54
scal01025	216	121	2,58	50S ribosomal protein L22
scal02333	217	710	2,58	4Fe-4S ferredoxin, iron-sulfur binding:FAD dependent oxidoreductase
scal02526	218	292	2,56	putative cytochrome b6
scal04256	219	305	2,56	expressed hypothetical protein
scal03706	220	117	2,53	No hits found
scal04307	221	68	2,53	30S ribosomal protein S14
scal04176	222	170	2,53	peroxiredoxin family protein
scal01282	223	217	2,53	No hits found
scal03835	224	202	2,51	putative PAS/PAC sensor protein
scal02105	225	155	2,51	putative copper binding protein of the plastocyanin/azurin family
scal02849	226	112	2,49	conserved hypothetical protein
scal04306	227	78	2,49	undefined product
scal04217	228	182	2,49	No hits found
scal02533	229	242	2,46	conserved hypothetical protein-putative methyltransferase
scal01024	230	223	2,46	30S ribosomal protein S3
scal03973	231	96	2,44	No hits found
scal03348	232	203	2,42	hypothetical planctomyces protein with 1 cxxch motive
scal03786	233	162	2,42	strongly similar to dUTP pyrophosphatase
scal00994	234	405	2,42	putative nirJ heme d1 biosynthesis protein
scal03319	235	318	2,40	putative NAD-dependent epimerase/dehydratase
scal01466	236	288	2,37	Fe-S iron sulfur cluster assembly NifU-like protein
scal03783	237	213	2,37	expressed conserved hypothetical protein
scal01232	238	274	2,37	similar to kinesin light chain KLC
scal02756	239	522	2,37	similar to DNA polymerase III subunit gamma/tau
scal02415	240	601	2,35	strongly similar to GTP-binding protein TypA

scal00454	241	488	2,35	expressed putative multiheme cytochrome c protein with 5 cxxch motives
scal02428	242	400	2,35	putative multiheme cytochrome c protein with 11 cxxch motives
scal00472	243	435	2,35	conserved hypothetical protein
scal02403	244	541	2,35	kusta0023 IlvD;strongly similar to dihydroxyacid dehydratase;amino acid metabolism;4.2.1.9
scal02330	245	278	2,35	conserved hypothetical protein
scal02080	246	189	2,33	expressed hypothetical protein
scal03313	247	305	2,28	putative nucleoside-diphosphate sugar epimerase
scal00342	248	135	2,28	conserved hypothetical protein
scal02057	249	200	2,28	putative COG1310 protease
scal01730	250	1436	2,26	glutamate synthase
scal01023	251	139	2,26	50S ribosomal protein L16/L10E
scal01217	252	130	2,26	conserved hypothetical protein
scal01536	253	99	2,24	thioredoxin
scal01578	254	296	2,21	FAD-dependent pyridine nucleotide-disulphide oxidoreductase
scal03118	255	304	2,21	protein of unknown function DUF980
scal02375	256	141	2,19	similar to unknown protein involved in septum location
scal01210	257	653	2,19	polynucleotide phosphorylase (PNPase)
scal02548	258	364	2,19	formate dehydrogenase alpha subunit
scal01573	259	172	2,19	rna binding protein
scal01750	260	262	2,17	hypothetical protein GbemDRAFT_2771
scal02591	261	247	2,17	conserved hypothetical protein
scal02358	262	95	2,17	pterin-4-alpha-carbinolamine dehydratase
orf06475	263	61	2,17	No hits found
scal02086	264	283	2,17	strongly similar to acetyl-CoA carboxylase carboxyltransferase alpha chain
scal03846	265	98	2,17	preprotein translocase, YajC subunit
scal00346	266	388	2,14	expressed threonine synthase thrC
scal03359	267	120	2,14	similar to ATPC gene encoding subunit epsilon of ATP synthase
scal01005	268	302	2,12	DNA-directed RNA polymerase, alpha chain
orf07451	269	82	2,12	hypothetical protein CLOHYLEM_03944
scal03122	270	173	2,12	KpsF/GutQ
scal03756	271	119	2,12	small heat shock protein
scal00953	272	146	2,12	cytochrome c-552 ks_3358
scal04320	273	54	2,12	Expressed conserved protein
scal03187	274	428	2,12	putative membrane protease
scal02104	275	267	2,10	hypothetical cgg1413 HEAT repeat protein
scal01486	276	400	2,10	conserved hypothetical protein
scal03502	277	1243	2,10	N terminal part of putative coenzyme a (CoA) enzyme activase
scal02490	278	220	2,10	5-methyltetrahydrofolate/corrinoid Fe-S protein methyltransferase (acsE) of the CODH/ACS complex
scal02831	279	153	2,10	glycine cleavage system H protein
scal02795	280	504	2,10	chaperonin GroEL
orf00368	281	52	2,08	No hits found
scal02009	282	105	2,08	two-component hybrid sensor and regulator
scal01022	283	107	2,08	ribosomal protein
scal04319	284	136	2,08	biotin carboxyl carrier protein (BCCP) of acetyl-CoA carboxylase
scal01402	285	263	2,05	strongly similar to hydroxymethylbilane synthase (porphobilinogen deaminase)
scal02631	286	364	2,05	biotin carboxylase (A subunit of acetyl-CoA carboxylase)
scal02283	287	122	2,05	50S ribosomal protein L11
scal01433	288	436	2,05	Phosphoglycerate kinase
scal01128	289	288	2,03	putative ATP:guanido phosphotransferase / arginine kinase

scal00995	290	326	2,03	aspartate transaminase
scal00193	291	413	2,03	similar to diaminopimelate decarboxylase lysa
scal02168	292	163	2,03	peptidyl-tRNA hydrolase
scal02053	293	194	2,01	putative ubiquinol:cytochrome c oxidoreductase of bc1 complex qcrC
scal02810	294	381	2,01	6-phosphogluconate dehydrogenase
scal02077	295	127	2,01	hypothetical protein PH0719
scal02245	296	137	2,01	unknown protein with 1 cxxch
scal00203	297	361	2,01	expressed glutamate-1-semialdehyde aminomutase
scal01028	298	187	2,01	50S ribosomal protein L4
scal01640	299	349	2,01	putative NADH:ubiquinone oxidoreductase, hydrogenase, component E-formate hydrogenlysase
scal03356	300	78	2,01	ATP synthase F0, C subunit
scal02932	301	409	2,01	similar to HtrA-like protein
scal00657	302	283	2,01	expressed putative nickel insertase (cooC) of CODH/ACS complex
scal00548	303	280	2,01	similar to octaprenyl diphosphate synthase
scal03009	304	403	2,01	putative histidine kinase
scal02427	305	339	1,99	expressed conserved hypothetical protein
scal03192	306	323	1,99	phosphoglycerate kinase
scal02402	307	417	1,99	conserved hypothetical protein
scal01344	308	709	1,96	putative multiheme protein with 14 cxxch motives
scal01161	309	668	1,96	oxygen sensitive ribonucleoside triphosphate reductase
scal01316	310	344	1,96	tryptophan syntase beta chain
scal01411	311	340	1,96	strongly similar to UDP-N-acetylglucosamine 1-carboxyvinyltransferase (enolpyruvyltransferase)
scal00309	312	113	1,94	158aa long hypothetical protein
scal02153	313	500	1,94	cell division protein FtsH
scal01872	314	295	1,94	aspartate-semialdehyde dehydrogenase Asd
scal02525	315	144	1,94	putative Rieske 2Fe-2S iron sulfur protein of bc1 complex
scal02661	316	701	1,94	SecA subunit of the preprotein translocase
scal00757	317	118	1,92	transcription elongation factor greA
scal00787	318	213	1,92	hypothetical protein
scal01487	319	422	1,92	putative glutamate synthase
scal00946	320	228	1,92	similar to rhomboid protein (intramembrane serine protease)
scal03518	321	649	1,92	DNA gyrase subunit A, type II topoisomerase
orf05337	322	50	1,89	conserved hypothetical protein
scal00687	323	243	1,89	conserved hypothetical protein of nar cluster
scal02071	324	354	1,87	strongly similar to thiamin biosynthesis protein ThiC
scal00587	325	387	1,87	amtB ammonium transport protein
orf02623	326	79	1,87	similar to 30S ribosomal protein RpsT
scal01027	327	69	1,85	strongly similar to 50S ribosomal protein L23
scal00852	328	258	1,85	similar to general secretory pathway protein K
orf01453	329	63	1,85	unknown protein
scal03812	330	202	1,85	protein of unknown function DUF191
scal03016	331	210	1,83	nitrite/sulfite reductase protein, NirB
scal03049	332	302	1,83	cysteine desulfurase
scal02069	333	86	1,83	methionyl-tRNA synthetase (mets)
scal03503	334	127	1,83	3-isopropylmalate dehydratase small subunit
scal01482	335	123	1,83	transcriptional regulator, TraR/DksA family
scal00926	336	142	1,83	unknown protein
scal00903	337	110	1,80	conserved hypothetical protein
scal03704	338	350	1,80	Hypothetical protein

scal00705	339	307	1,80	putative iron sulfur
orf02276	340	54	1,80	No hits found
scal00944	341	487	1,80	chaperone Hsp70
scal02566	342	170	1,80	No hits found
scal01544	343	254	1,80	similar to pyridoxal phosphate biosynthetic protein pdxA
scal00011	344	151	1,80	expressed putative dinB protein
scal03068	345	243	1,78	putative stress protein
scal00786	346	608	1,78	V-type H(+)-translocating pyrophosphatase
scal02398	347	275	1,78	ketol-acid reductoisomerase
scal02089	348	210	1,78	similar to phosphoribosylformylglycinamidine synthase I
scal02101	349	282	1,78	SAM radical nirJ like heme d synthesis protein
scal02749	350	234	1,78	strongly similar to pyrroline-5-carboxylate reductase
scal03863	351	359	1,78	thiamine biosynthesis protein ThiC
orf05842	352	61	1,78	Uncharacterized conserved protein UCP037465, zinc finger protein, AF1427
scal01731	353	360	1,78	glutamate synthase
scal00655	354	108	1,78	Putative Holliday junction resolvase
scal00246	355	500	1,76	expressed type III glutamate-ammonia ligase
scal00238	356	189	1,76	hypothetical protein
scal00367	357	265	1,76	strongly similar to pleiotrophic regulatory protein DegT
scal01579	358	418	1,76	Pyruvate ferredoxin/flavodoxin oxidoreductase:Pyruvate flavodoxin/ferredoxin oxidoreductase, N-terminal
scal00215	359	886	1,76	expressed pyruvate:ferredoxin oxidoreductase
scal03031	360	112	1,76	putative Rubrerythrin
scal02033	361	177	1,73	putative cobalt transport protein CbiQ
scal00860	362	211	1,73	similar to ATPase involved in chromosome partitioning
orf04379	363	54	1,73	hypothetical protein
scal02335	364	197	1,73	putative iron sulfur heterodisulfide reductase subunit
scal01543	365	107	1,73	putative flavoprotein norVW
scal04060	366	96	1,73	hypothetical protein Sfum_1584
scal02799	367	275	1,73	strongly similar to heat shock protein DnaJ
scal01960	368	67	1,71	similar to flagellar hook-basal body complex protein FliE
orf05825	369	55	1,71	conserved hypothetical protein
scal01681	370	336	1,71	ammonium transport protein
scal02494	371	452	1,71	Phosphoenolpyruvate carboxykinase (GTP)
scal02764	372	390	1,71	delta-1-pyrroline-5-carboxylate dehydrogenase
scal01587	373	107	1,71	conserved hypothetical protein
scal01016	374	128	1,71	50S ribosomal protein L6
scal01564	375	195	1,71	conserved hypothetical protein
scal02486	376	297	1,71	large subunit corrinoid FeS protein of CODH/ACS complex(ascC)
scal02527	377	439	1,71	putative NAD(P) oxidoreductase, FAD-containing subunit
scal03919	378	151	1,69	hypothetical duf1318 membrane protein
scal00963	379	243	1,69	hypothetical protein L8106_28486
scal00999	380	236	1,69	strongly similar to the signal recognition particle receptor of E. coli.
scal03551	381	367	1,69	hypothetical Na+/H+ antiporter ArsB
scal01645	382	103	1,69	conserved hypothetical protein of nirHD gene cluster involved in heme d1 biosynthesis
scal00893	383	55	1,69	unknown protein
scal00127	384	106	1,69	conserved hypothetical protein
scal03582	385	111	1,69	unknown protein
scal04170	386	235	1,67	hypothetical protein VvadDRAFT_2521
scal04193	387	400	1,67	30S ribosomal protein S1

scal00608	388	297	1,67	MORN repeat protein
scal00586	389	171	1,67	putative MazG like pyrophosphatase with methyltransferase domain
scal00605	390	101	1,67	hypothetical protein STIAU_5686
scal01748	391	735	1,67	PKD domain containing protein
scal03542	392	198	1,64	strongly similar to glycine tRNA synthetase, alpha subunit
scal02347	393	183	1,64	mannosyl-3-phosphoglycerate phosphatase
scal01194	394	314	1,64	ATP-dependent Hsl protease
scal01021	395	80	1,64	50S ribosomal protein L14
orf02596	396	61	1,62	conserved hypothetical protein
scal03023	397	313	1,62	aspartate ammonia-lyase (aspartase)
scal00287	398	259	1,62	putative hlyD secretion protein
scal02565	399	169	1,62	No hits found
scal00693	400	156	1,62	putative monoheme protein
scal02735	401	194	1,62	expressed conserved hypothetical protein
scal01017	402	93	1,62	30S ribosomal protein S8
scal04133	403	297	1,60	putative hydroxylamine oxidoreductase hao 8 cxxch motives
scal03186	404	272	1,60	expressed conserved hypothetical protein
scal00382	405	296	1,60	similar to ATP-binding protein Wzt of ABC-transporter involved in LPS biosynthesis
scal02180	406	163	1,60	uridylate kinase
scal01001	407	109	1,57	strongly similar to riboflavin synthase of Aquifex aeolicus
scal02152	408	167	1,57	hypothetical COG0217 protein
scal00661	409	130	1,57	expressed putative nuoE of NADH:ubiquinone oxidoreductase
scal01102	410	314	1,57	cysteinyl-tRNA synthetase
scal00615	411	123	1,57	phosphoribosylcarboxyaminoimidazole carboxylase
scal02376	412	374	1,57	acetohydroxy acid synthase IilvB
scal02179	413	130	1,57	elongation factor Ts (EF-Ts)
scal03485	414	71	1,57	putative molybdopterin-containing NuoG subunit of NADH:ubiquinone oxidoreductase
scal04281	415	410	1,57	hypothetical protein
scal04036	416	208	1,57	expressed conserved hypothetical protein with MoxR-like ATPase domain
scal00659	417	655	1,55	expressed FAD-dependent pyridine nucleotide-disulphide oxidoreductase
orf03345	418	34	1,55	No hits found
scal02255	419	242	1,55	diaminohydroxyphosphoribosylaminopyrimidine deaminase / 5-amino-6-(5-phosphoribosylamino)uracil reductase
scal00557	420	487	1,55	putative thioredoxin
scal02391	421	203	1,55	pantoate--beta-alanine ligase
scal02040	422	369	1,53	similar to cell division protein FtsI
scal04032	423	207	1,53	putative ABC type transport protein, ATPase component
scal02794	424	74	1,53	unknown anammox protein
scal00409	425	144	1,53	expressed fructose-6-phosphate aldolase
scal02096	426	161	1,53	strongly similar to glutamine amidotransferase class I
scal00925	427	97	1,51	No hits found
scal04323	428	47	1,51	conserved hypothetical protein
scal02485	429	122	1,51	ATP-dependent endopeptidase Clp of CO dehydrogenase cluster
scal04125	430	206	1,51	hypothetical protein CLOL250_02573
scal02236	431	102	1,51	putative nirD heme d1 biosynthesis protein
scal02100	432	274	1,51	putative nirJ heme d1 biosynthesis protein
scal04039	433	318	1,51	SAM radical protein; putaive cobalamin b12 binding
scal01077	434	81	1,51	transcriptional regulator, TraR/DksA family
scal03180	435	182	1,51	unknown protein
scal02037	436	301	1,51	putative carbohydrate-selective porin protein, OprB family

scal02899	437	204	1,51	putative acetylglutamate kinase
scal02926	438	166	1,48	conserved hypothetical protein
scal03306	439	493	1,48	strongly similar to DNA gyrase subunit B
scal00401	440	420	1,48	hypothetical protein
scal00239	441	177	1,48	expressed putative PpiC-type peptidyl-prolyl cis-trans isomerase
scal02911	442	275	1,48	hypothetical protein
scal01180	443	210	1,46	hypothetical morn protein
scal01054	444	162	1,46	putative zinc finger protein
scal02430	445	380	1,46	putative succinate dehydrogenase subunit A
scal01644	446	199	1,46	stongly similar to NADH dehydrogenase subunit
orf00346	447	46	1,46	conserved hypothetical protein
scal01059	448	152	1,46	putative sugar phosphate isomerase
scal00190	449	139	1,46	Phosphoglycerate/bisphosphoglycerate mutase
scal03358	450	60	1,46	conserved hypothetical membrane protein
scal00428	451	236	1,44	putative cell shape-determining protein MreB
scal01565	452	188	1,44	methionyl-tRNA formyltransferase
scal03899	453	74	1,44	No hits found
scal03360	454	286	1,44	ATP synthase subunit B
orf03683	455	44	1,44	No hits found
scal00218	456	135	1,44	expressed unknown protein
scal00610	457	104	1,44	PREDICTED: similar to CG13731-PA
scal00362	458	397	1,44	strongly similar to 1-deoxy-D-xylulose 5-phosphate synthase (DXP synthase)
scal02763	459	186	1,44	Nitrilase/cyanide hydratase and apolipoprotein N-acyltransferase
scal00375	460	199	1,44	pdb|1NA0|A Chain A, Design Of Stable Alpha-Helical Arrays From An Idealized Tpr Motif
scal02611	461	215	1,44	surA peptidyl-prolyl cis-trans isomerase
scal00737	462	196	1,44	phosphate-starvation inducible protein PhoH
scal01031	463	77	1,44	30S ribosomal protein S10
scal04294	464	78	1,44	unknown protein containing one cxxch motive
scal03898	465	460	1,41	conserved hypothetical protein
scal02861	466	535	1,41	formate dehydrogenase, alpha subunit
scal03931	467	69	1,41	unknown protein
scal04253	468	183	1,41	hypothetical protein MTH1105
scal01408	469	95	1,41	50S ribosomal protein L19
scal02629	470	145	1,39	putative tpr repeat protein
scal03501	471	241	1,39	3-isopropylmalate dehydratase, large subunit
scal02928	472	255	1,39	similar to carboxy-terminal processing protease
scal01195	473	138	1,39	conserved hypothetical protein
scal00408	474	114	1,39	bifunctional cobinamide kinase/cobinamide phosphate guanylyltransferase
scal01181	475	170	1,39	surface antigen BspA-like
scal02169	476	81	1,39	30S ribosomal subunit protein S6
scal02095	477	125	1,39	flagellar motor protein motB
scal03002	478	172	1,39	RPGR
scal02240	479	282	1,37	putative Zn-dependent protease
scal02178	480	138	1,37	30S ribosomal protein S2
scal02160	481	158	1,37	putative sensor protein
scal00416	482	216	1,37	expressed formate/nitrite transporter family protein focA
scal00931	483	69	1,37	similar to nitrogen regulatory protein P-II family proteins
scal01134	484	73	1,37	hypothetical protein ROS217_08820
scal00200	485	96	1,37	expressed B subunit of F0F1 ATP synthase

scal00339	486	274	1,37	hemA glutamyl-tRNA reductase
scal00590	487	72	1,37	GlnK nitrogen regulatory protein P-II
scal01641	488	101	1,37	similar to to proton-translocating NADH dehydrogenase I, 24 kDa subunit (NuoE)
scal01387	489	82	1,37	unknown protein
scal00223	490	81	1,37	S-adenosylmethionine decarboxylase
scal02133	491	291	1,35	bifunctional purine biosynthesis protein phosphoribosylaminoimidazolecarboxamide formyltransferase (AICAR transformylase); IMP cyclohydrolase
scal04114	492	203	1,35	conserved hypothetical protein
scal02078	493	184	1,35	strongly similar to aspartate transcarbamoylase catalytic chain
scal01315	494	143	1,35	tryptophan synthase alpha chain
scal02665	495	222	1,35	putative bifunctional GTP cyclohydrolase II / 3,4-dihydroxy-2-butanone 4-phosphate synthase
scal00859	496	168	1,35	similar to molybdenum cofactor biosynthesis protein A
scal01034	497	294	1,35	hypothetical protein
scal03553	498	260	1,35	hypothetical protein
scal00197	499	178	1,35	expressed gamma subunit of F1F0 ATP synthase
scal03424	500	127	1,35	hypothetical protein Sfum_0344
scal03691	501	130	1,35	putative norVW-like flavodoxin protein
orf05822	502	49	1,35	conserved hypothetical protein
scal03794	503	102	1,32	conserved hypothetical protein
scal02039	504	62	1,32	No hits found
scal02337	505	167	1,32	putative iron sulfur heterodisulfide reductase gamma subunit
scal04309	506	46	1,32	30S ribosomal protein S19
scal04255	507	79	1,32	glycoside hydrolase, family 13-like
scal03788	508	102	1,32	strongly similar to guanylate kinase
scal01131	509	67	1,32	conserved hypothetical protein
scal02949	510	181	1,32	conserved hypothetical protein
orf04899	511	41	1,32	No hits found
scal01098	512	99	1,32	membrane protein of unknown function (DUF1430)
scal00474	513	142	1,32	conserved hypothetical cog1611 protein
scal03147	514	80	1,30	unknown protein
scal00662	515	137	1,30	histidine biosynthesis protein hisF imidazole glycerol phosphate synthase
scal00153	516	135	1,30	unknown protein
scal02909	517	280	1,30	similar to isopropylmalate synthase
scal03946	518	191	1,30	similar to site-specific tyrosine recombinase
scal03183	519	139	1,28	hypothetical protein XC_3267
scal00031	520	383	1,28	strongly similar to ATP-dependent DNA helicase
scal01580	521	164	1,28	2-oxoglutarate ferredoxin oxidoreductase
scal01003	522	84	1,28	conserved hypothetical protein
scal03815	523	399	1,28	putative TPR+C255 repeat protein
scal02036	524	144	1,28	putative cobalamin biosynthesis and cobalt transport protein CbiM
scal02091	525	301	1,28	putative iron sulfur NuoF subunit of NADH:ubiquinone oxidoreductase
orf02370	526	45	1,28	strongly similar to 30S ribosomal protein S16
scal02910	527	458	1,28	strongly similar to valyl-tRNA synthetase
scal01639	528	91	1,28	strongly similar to to proton-translocating NADH dehydrogenase I chain C (NuoC)
scal01577	529	337	1,28	putative NAD(P) oxidoreductase, FAD-containing subunit
scal01380	530	70	1,28	No hits found
scal02397	531	288	1,28	strongly similar to 2-isopropylmalate synthase
scal04293	532	88	1,26	putative cytochrome c protein C2
scal02247	533	99	1,26	phosphoheptose isomerase
scal00771	534	120	1,26	BBMII isomerase

scal03792	535	152	1,26	triose-phosphate isomerase
scal00993	536	182	1,26	RecA protein (recombinase A)
scal00579	537	118	1,26	conserved hypothetical protein
scal03647	538	135	1,26	protein chain release factor 2 (RF-2)
scal03191	539	176	1,26	glyceraldehyde-3-phosphate dehydrogenase
scal02833	540	222	1,26	glutamyl-tRNA synthetase
scal02041	541	246	1,26	UDP-N-acetylmuramyl tripeptide synthase
scal02955	542	229	1,26	phosphoglucosamine mutase
scal02715	543	164	1,26	thioredoxin
scal04154	544	79	1,26	PTS system, fructose-specific enzyme II, BC component
scal02628	545	250	1,26	aldehyde dehydrogenase
scal03305	546	56	1,23	hypothetical protein
scal01160	547	85	1,23	putative NrdR-like ribonucleotide reductase regulator
scal04052	548	85	1,23	putative transcriptional regulator
scal04311	549	35	1,23	rubredoxin-type Fe(Cys)4 protein
scal02395	550	95	1,23	sur A peptidyl-prolyl cis-trans isomerase
scal04142	551	173	1,23	similar to oxoacid:ferredoxin oxidoreductase gamma chain
scal04251	552	67	1,23	response regulator protein
scal03308	553	153	1,23	hypothetical protein
scal02843	554	108	1,23	hypothetical protein Mpe_B0252
scal01570	555	185	1,23	No hits found
scal03317	556	380	1,21	Pentapeptide repeat
scal00857	557	304	1,21	glutaminyl-tRNA synthetase
scal00614	558	148	1,21	similar to MTA/SAH nucleosidase
scal01985	559	195	1,21	putative flagellin
scal03007	560	216	1,21	nitrite extrusion protein narK; nitrate nitrite antiport protein
scal02929	561	150	1,21	similar to riboflavin biosynthesis protein RibF
scal02682	562	35	1,21	putative cold shock protein CspB
scal02753	563	258	1,21	silmilar to D-aminoacylase
scal00861	564	100	1,21	putative mauM napG like ferredoxin protein
scal00479	565	62	1,19	No hits found
orf02355	566	33	1,19	cold-shock DNA-binding domain protein
scal03602	567	108	1,19	conserved hypothetical sugar phosphate isomerase protein
scal04123	568	153	1,19	hypothetical protein
scal03930	569	195	1,19	cytochrome c peroxidase
scal04218	570	178	1,19	conserved hypothetical protein of unknown function (DUF1432)
scal01148	571	91	1,19	similar to pyruvoyl-dependent arginine decarboxylase
scal00212	572	302	1,19	strongly similar to ATP-dependent zinc-metalloprotease ftsH involved in cell division
scal01585	573	191	1,19	cobalt-zinc-cadmium resistance protein czcD
scal02379	574	278	1,19	unknown protein
scal03594	575	204	1,19	putative PAS/PAC sensor protein
scal01209	576	40	1,19	ribosomal protein S15
scal03646	577	211	1,19	serine hydroxymethyl transferase SHMT
scal01349	578	59	1,19	sensory box histidine kinase/response regulator
scal00045	579	61	1,19	Glyoxalase/bleomycin resistance protein/dioxygenase
scal01343	580	93	1,19	putative redoxin domain protein
scal02898	581	187	1,19	acetylornithine aminotransferase
scal04149	582	183	1,16	strongly similar to Obg GTP-binding protein
scal00607	583	227	1,16	strongly similar to sigma 54 response regulator

scal04163	584	54	1,16	putative periplasmic lipoprotein
scal02986	585	189	1,16	cell surface protein
scal01467	586	216	1,16	putative cysteine desulfurase nifS
scal01401	587	233	1,16	strongly similar to 3-phosphoshikimate 1-carboxyvinyltransferase
scal00280	588	39	1,16	unknown protein
scal02468	589	384	1,16	expressed hypothetical protein
scal02840	590	96	1,16	expressed conserved hypothetical protein, MarR family
scal00273	591	86	1,16	putative deoxycytidylate deaminase comEB
scal02380	592	183	1,16	TPR Domain containing protein
scal00583	593	217	1,16	unknown protein
scal00574	594	108	1,16	phage shock protein A, PspA
scal00026	595	68	1,14	conserved hypothetical protein
scal01280	596	409	1,14	ref|ZP_00109444.2| COG5651: PPE-repeat proteins
scal03708	597	311	1,14	putative ammonium transport protein
scal01096	598	203	1,14	strongly similar to ATPase subunit
scal01351	599	133	1,14	putative signal transduction protein
scal02626	600	100	1,14	hypothetical protein
scal00271	601	83	1,14	putative peptide methionine sulfoxide reductase
scal03355	602	123	1,14	B subunit of F0 ATP synthase
scal01550	603	145	1,14	conserved hypothetical protein
scal01409	604	110	1,14	strongly similar to tRNA (guanine-N(1)-)-methyltransferase
scal00555	605	126	1,12	adenylylsulfate reductase, thioredoxin dependent
scal02299	606	67	1,12	universal stress protein uspA
scal00541	607	140	1,12	UDP-N-acetylenolpyruvoylglucosamine reductase
scal01243	608	199	1,12	2-phosphoglycerate dehydratase, enolase
scal02641	609	66	1,12	V-type ATPase subunit K
scal04317	610	21	1,12	conserved protein of unknown function
scal02887	610	203	1,12	transcription termination factor Rho
orf07166	612	41	1,12	similar to acyl carrier protein
scal04151	613	241	1,12	strongly similar to ribonuclease E (RNase E)
scal01133	614	249	1,12	D-3-phosphoglycerate dehydrogenase
scal00435	615	233	1,12	expressed putative PpiC-type peptidyl-prolyl cis-trans isomerase protein
scal01352	616	162	1,12	strongly imilar to aspartate aminotransferase
scal00175	617	78	1,12	peptidyl-prolyl cis-trans isomerase A
scal01586	618	71	1,12	hypothetical protein lpp2861
scal02173	619	348	1,12	putative outer membrane protein Omp85
scal01152	620	231	1,12	uroporphyrinogen III synthase/methyltransferase
scal03426	621	281	1,12	hypothetical protein Bpse110_02003470
scal00032	622	203	1,12	expressed iron-sulfur protein NifS
scal01076	623	258	1,12	phosphoglucomutase, alpha-D-glucose phosphate-specific
scal02084	624	77	1,12	No hits found
scal00308	625	187	1,10	conserved hypothetical protein
scal00208	626	222	1,10	conserved hypothetical protein
scal02336	627	150	1,10	putative 4Fe-4S ferredoxin hydrogenase beta subunit
scal01157	628	111	1,10	conserved hypothetical protein
scal03830	629	179	1,10	ATP-independent RNA helicase DbpA
scal00919	630	209	1,10	glycolate oxidase subunit GlcD
scal02464	631	307	1,10	sulfate adenylyltransferase, large subunit subfamily, putative
scal03030	632	68	1,10	conserved hypothetical protein

scal01290	633	205	1,10	similar to capsular polysaccharide synthesis protein
scal00845	634	162	1,10	TPR repeat:Peptidase M, neutral zinc metallopeptidases, zinc-binding site
scal03216	635	283	1,10	hypothetical protein
scal04112	636	254	1,10	conserved hypopethical protein
scal02075	637	223	1,07	conserved hypothetical protein
orf04907	638	91	1,07	No hits found
scal04095	639	203	1,07	cobyrinic acid A,C-diamide synthase
scal01379	640	116	1,07	hypothetical protein
orf02364	641	31	1,07	No hits found
scal02134	642	113	1,07	putative membrane-bound lytic murein transglycosylase A
scal02796	643	50	1,07	No hits found
scal02364	644	67	1,07	DNA polymerase, beta domain protein region
scal00876	645	116	1,07	hypothetical protein
scal00046	646	143	1,07	putative peptidase
scal01297	647	161	1,07	3-deoxy-D-arabino-heptulosonate 7-phosphate synthase
scal02308	648	368	1,07	aconitate hydratase
scal00747	649	236	1,07	glutamate dehydrogenase, short peptide
scal03051	650	126	1,07	CO dehydrogenase/acetyl-CoA synthase alpha subunit acsB
scal03357	651	104	1,07	A subunit of FO ATP synthase
scal00616	652	161	1,05	conserved hypothetical protein
scal02092	653	107	1,05	hypothetical protein Acid345_2344
scal02889	654	218	1,05	conserved hypothetical HD KH hydrolase protein
scal01542	655	79	1,05	putative rubrerythrin/rubredoxin protein
scal00537	656	69	1,05	expressed conserved hypothetical COG2001 protein
scal03700	657	399	1,05	conserved hypothetical signal transduction histidine kinase BaeS/NtrB
scal01456	658	163	1,05	similar to glucokinase
scal04182	659	54	1,05	unknown protein
scal02349	660	277	1,05	excinuclease ABC subunit B
scal00016	661	202	1,05	pyridine nucleotide-disulphide oxidoreductase dimerisation region
scal02346	662	195	1,05	putative glycosyltransferase involved in cell wall biogenesis
scal03015	663	57	1,05	lactoylglutathione lyase
scal03912	664	261	1,03	hypothetical protein
scal00937	665	62	1,03	hypothetical protein Gmet_1606
scal00596	666	261	1,03	amtB ammonium transport protein
scal00943	667	369	1,03	ATP-dependent protease
scal03379	668	341	1,03	putative COG2303 Choline dehydrogenase and related flavoproteins
scal02598	669	131	1,03	Integron integrase
scal00185	670	360	1,03	putative catalase/peroxidase
scal00897	671	154	1,03	strongly similar to nicotinate-nucleotide-dimethylbenzimidazole phosphoribosyltransferase
scal04143	672	104	1,03	similar to pyruvate ferredoxin oxidoreductase beta subunit
scal01642	673	283	1,03	similar to proton-translocating NADH dehydrogenase I, 51 kDa subunit (NuoF)
scal02896	674	120	1,03	ribonuclease PH
scal02269	675	207	1,03	strongly similar to glutamine phosphoribosylpyrophosphate amidotransferase
scal01537	676	100	1,03	thiol:disulfide interchange protein
scal02370	677	102	1,03	ATP-dependent endopeptidase Clp
scal04035	678	321	1,03	unknown protein
scal01873	679	134	1,00	unknown protein
scal03315	680	175	1,00	hypothetical protein
scal04066	681	153	1,00	DegT/DnrJ/EryC1/StrS aminotransferase

scal00759	682	53	1,00	No hits found
scal01558	683	72	1,00	putative carbon monoxide dehydrogenase, iron sulfur subunit CooF
scal01079	684	45	1,00	unknown protein
scal04096	685	54	1,00	conserved hypothetical protein
scal01314	686	84	1,00	rieske iron sulfur (2Fe-2S) protein of the bc1 complex
scal02252	687	95	1,00	putative protein kinase (DUF1566)
scal02487	688	285	1,00	putative iron sulfur / metal binding protein of the CODH/ACS complex
scal03208	689	164	1,00	hypothetical protein
scal02176	690	113	1,00	UDP-N-acetylglucosamine acetyltransferase
scal03591	691	84	1,00	cytoplasmic membrane protein
scal00510	692	127	1,00	similar to Na(+)-translocating NADH-quinone reductase subunit A
scal02930	693	230	1,00	expressed hypothetical protein
scal03986	694	314	1,00	RNA binding S1
scal02260	695	158	0,98	strongly similar to chorismate synthase
scal03603	696	160	0,98	phosphopentomutase
scal03600	697	127	0,98	similar to dTDP-glucose 4,6-dehydratase
scal04147	698	62	0,98	hypothetical protein
scal00357	699	142	0,98	fibronectin type III domain containing protein
scal01455	700	135	0,98	Ppx/GppA phosphatase
scal04002	701	138	0,98	Oligopeptide/dipeptide ABC transporter, ATP-binding protein
scal02931	702	312	0,98	DNA topoisomerase I
scal00808	703	149	0,98	similar to site-specific tyrosine recombinase
scal03182	704	189	0,98	similar to 7,8-diaminopelargonic acid synthase
scal03188	705	220	0,98	hypothetical protein
scal01234	706	74	0,96	CO dehydrogenase/acetyl-CoA synthase alpha subunit (acsB)partial C-terminus
scal02504	707	198	0,96	DEAD/DEAH box helicase-like protein
scal00282	708	83	0,96	unknown protein
scal01479	709	138	0,96	thioredoxin reductase
scal04195	710	75	0,96	putative phospholipase D / transphosphatidylase
scal03928	711	63	0,96	No hits found
scal00324	712	109	0,96	expressed malonyl-CoA-[acyl-carrier-protein] transacylase
scal03911	713	172	0,96	putative 1-deoxy-D-xylulose-5-phosphate reductoisomerase
scal02941	714	128	0,96	conserved hypothetical protein, radical SAM family protein
orf06298	715	61	0,96	heat shock protein DnaJ domain-containing protein
scal02268	716	145	0,96	strongly similar to phosphoribosylformylglycinamidine cyclo-ligase
scal03759	717	388	0,94	fructose-bisphosphatase
scal02467	718	240	0,94	similar to multidrug resistance ABC-transporter ATP binding protein
scal04192	719	68	0,94	adenine phosphoribosyltransferase
scal00096	720	105	0,94	unknown protein
scal01075	721	59	0,94	universal stress protein uspA
scal03814	722	116	0,94	cysteine synthase, O-acetylserine (thiol) lyase B
scal01182	723	139	0,94	GTP binding protein
scal02603	724	278	0,94	hypothetical protein
scal03811	725	99	0,94	hypothetical protein TTC0188
scal01697	726	47	0,94	conserved hypothetical protein
scal04141	727	217	0,94	phosphoglyceromutase
scal01959	728	65	0,94	flagellar hook protein FlgF
orf05355	729	57	0,94	conserved hypothetical protein
scal03689	730	143	0,94	Peptide methionine sulfoxide reductase

scal02987	731	151	0,94	similar to selenocysteine lyase
scal02808	732	279	0,94	transketolase
scal03546	733	44	0,94	No hits found
scal02234	734	61	0,94	hypothetical protein YajQ
scal03923	735	126	0,94	geranylgeranyl pyrophosphate (GGPP) synthetase
scal01227	736	94	0,94	putative TPR repeat containing methyltransferase
scal01386	737	160	0,94	argininosuccinate synthetase
scal02325	738	233	0,94	expressed conserved hypothetical protein BatD
scal02574	739	189	0,94	strongly similar to lysyl-tRNA synthetase
scal02623	740	116	0,91	hypothetical protein
scal04274	741	217	0,91	D-xylulose 5-phosphate/D-fructose 6-phosphate phosphoketolase family protein
scal00125	742	106	0,91	molybdenum ABC transporter, periplasmic molybdate-binding protein
scal02484	743	90	0,91	CO dehydrogenase/acetyl-CoA synthase alpha subunit acsB C-terminal
scal00992	744	64	0,91	probable osmotically inducible protein Y
scal02674	745	225	0,91	putative ABC transporter ATP-binding protein
scal00794	746	275	0,91	putative murI glutamate racemase fusion protein
scal01286	747	275	0,91	NAD(P) oxidoreductase, FAD-containing subunit, 4Fe-4S ferredoxin, iron-sulfur binding
scal04229	748	135	0,91	hypothetical protein
orf05399	749	17	0,91	superoxide reductase-like protein
scal00434	750	196	0,91	expressed aminopeptidase A
scal01106	751	130	0,91	two component, sigma54 specific, transcriptional regulator, Fis family
scal00274	752	72	0,91	expressed flavoredoxin
scal03529	753	121	0,91	similar to quinolinate synthetase A
scal03840	754	176	0,91	putative DNA directed RNA polymerase
scal02807	755	136	0,91	putative glucokinase
scal00515	756	108	0,91	3-oxoacyl acyl-carrier protein reductase
scal01696	757	69	0,91	MOSC domain containing protein
scal02492	758	25	0,91	No hits found
scal02334	759	61	0,91	putative Methyl-viologen-reducing hydrogenase, delta subunit, heterodisulfide reductase subunit D
scal03437	760	55	0,89	sensory box protein/sigma-54 dependent DNA-binding response regulator
scal03517	761	77	0,89	putative beta lactamase / hydroxyacylglutathione hydrolase
scal04153	762	257	0,89	strongly similar to phosphoenolpyruvate-protein phosphotransferase (phosphotransferase system, enzyme I)
scal00195	763	92	0,89	putative M22 glycoprotease protein
scal00430	764	162	0,89	similar to molybdenum cofactor biosynthesis protein
scal00694	765	168	0,89	universal stress protein UspA associated with nar cluster
scal01353	766	98	0,89	unknown protein
scal01100	767	180	0,89	similar to 2-acylglycerophosphoethanolamine acyltransferase/acyl carrier protein synthetase
scal04025	768	49	0,89	putative exonuclease III
scal00758	769	94	0,89	hypothetical protein
scal02528	770	112	0,89	putative bc1 protein
scal02488	771	100	0,89	putative nickel insertase (acsF) of CODH/ACS complex
scal00577	772	156	0,89	hypothetical protein
scal01678	773	174	0,89	group II intron, maturase
scal00558	774	42	0,89	expressed GlnK nitrogen regulatory protein P-II
scal01406	775	123	0,89	conserved hypothetical protein
scal01385	776	81	0,89	protein of unknown function DUF500
scal02311	777	274	0,89	chaperone endopeptidase Clp ATP-binding chain B,ClpB
scal01304	778	195	0,89	hypothetical protein, conserved
scal01122	779	83	0,89	similar to 2-C-methyl-D-erythritol 4-phosphate cytidylyltransferase

scal02736	780	30	0,89	unknown protein
scal04181	781	64	0,89	hypothetical protein cbdb_A797
scal03555	782	49	0,89	unknown protein
scal01643	783	327	0,89	similar to molybdopterin oxidoreductase, molybdopterin-containing subunit/ NuoG subunit of NADH dehydrogenase I
scal01156	784	72	0,89	conserved hypothetical protein
scal00198	785	276	0,89	expressed alpha subunit of F0F1 ATP synthase
scal01647	786	38	0,89	similar to NADH dehydrogenase I subunit K
scal00009	787	143	0,89	conserved hypothetical protein
scal02761	788	82	0,89	No hits found
scal02616	789	164	0,89	putative threonine synthase thrC
scal02950	790	110	0,87	unknown protein
scal02758	791	355	0,87	putative protease
scal03193	792	89	0,87	strongly similar to D-ribulose-5-phosphate 3-epimerase
scal02070	793	93	0,87	conserved hypothetical protein
scal04189	794	85	0,87	putative appr-1-p processing protein
scal02065	795	107	0,87	fructose-1,6-biphosphate aldolase Class I
scal03354	796	192	0,87	H(+)-transporting ATP synthase, subunit alpha
scal00584	797	71	0,87	expressed putative MotA/TolQ/ExbB biopolymer transport protein
scal01545	798	77	0,87	strongly similar to riboflavin synthase alpha chain
scal02172	799	162	0,87	replicative DNA helicase dnaB
scal00441	800	98	0,87	conserved hypothetical protein
scal01155	801	55	0,87	conserved hypothetical protein
scal01283	802	137	0,87	adhesin
scal04159	803	45	0,87	conserved hypothetical protein
scal03590	804	46	0,87	putative tonB receptor protein
scal00663	805	96	0,84	thiamin biosynthesis protein ThiG
orf03200	806	40	0,84	No hits found
scal00237	807	203	0,84	strongly similar to transcription initiation factor sigma RpoD
orf02595	808	20	0,84	RNA-binding protein, predicted
scal03361	809	54	0,84	hypothetical protein Ping_2356
scal02431	810	101	0,84	putative succinate dehydrogenase iron sulfur subunit
scal00539	811	28	0,84	No hits found
scal02103	812	47	0,84	putative cytochrome c CII protein
scal01281	813	185	0,84	No hits found
scal04191	814	89	0,84	hypothetical protein
scal04200	815	99	0,84	conserved hypothetical protein
scal03983	816	425	0,84	putative molybdopterin oxidoreductase / amino transferase protein
scal04062	817	37	0,84	unknown anammox protein
scal00081	818	86	0,84	formiminotetrahydrofolate cyclodeaminase
scal01830	819	71	0,84	protein of unknown function UPF0027
scal04001	820	119	0,84	oligopeptide ABC transporter ATP-binding protein
scal04183	821	114	0,84	conserved hypothetical protein
scal02501	822	54	0,84	No hits found
scal03842	823	113	0,84	strongly similar to RuvB ATPase
scal04043	824	51	0,84	strongly similar to hydroxymyristoyl acyl carrier protein dehydratase
scal02378	825	125	0,84	Nucleotidyl transferase
scal00521	826	325	0,84	expressed putative Zn dependent protease
scal02621	827	103	0,84	5'-methylthioadenosine phosphorylase
scal04197	828	115	0,84	hypothetical protein

scal00329	829	160	0,82	similar to histidyl-tRNA synthetase
scal01561	830	121	0,82	fructose-bisphosphate aldolase
scal02750	831	94	0,82	Predicted hydrolase
scal00414	832	23	0,82	hypothetical protein Gmet_1619
scal01608	833	87	0,82	putative cobalt transport ATP-binding protein CbiO
scal00763	834	120	0,82	hypothetical cytosolic protein
scal01350	835	180	0,82	response regulator receiver modulated diguanylate cyclase
scal00585	836	42	0,82	biopolymer transport ExbD protein
scal02882	837	101	0,82	ribokinase
scal03006	838	295	0,82	putative inorganic ion or dicarboxylate transport protein of TDT family
orf05365	839	18	0,82	similar to cbb3-type cytochrome c oxidase maturation protein
scal03029	840	161	0,82	FAD-dependent pyridine nucleotide-disulphide oxidoreductase
scal03480	841	124	0,82	Glutathione synthase
scal00326	842	204	0,82	expressed aspartyl-tRNA ligase aspS
scal02457	843	53	0,82	putative glycosyltransferase
scal02276	844	51	0,82	30S ribosomal protein S7
scal01008	845	115	0,82	conserved hypothetical protein
scal03294	846	106	0,80	Methenyltetrahydrofolate cyclohydrolase
scal00286	847	112	0,80	putative ABC transporter ATP binding protein
scal02056	848	61	0,80	Rieske iron sulfur protein
scal04326	849	184	0,80	alpha-glucan phosphorylase
scal02489	850	116	0,80	small subunit of corrinoid FeS protein of the CODH/ACS complex (acsD)
scal00869	851	161	0,80	sigma-54 dependent DNA-binding response regulator
scal02031	852	91	0,80	unknown protein
scal00363	853	140	0,80	strongly similar to exodeoxyribonuclease VII large chain Escherichia coli (K12)
scal03202	854	48	0,80	hypothetical protein Rxyl_1146
scal03847	855	381	0,80	putative protein-export membrane protein SecD
scal02673	856	125	0,80	hypothetical duf323 protein
scal03505	857	224	0,80	squalene hopene cyclase
scal03935	858	159	0,80	hypothetical protein C terminal
scal02386	859	29	0,80	hypothetical protein DoleDRAFT_1523
scal02035	860	42	0,80	conserved hypothetical protein
scal02575	861	75	0,80	putative ABC transporter protein
scal01700	862	144	0,80	hypothetical FAD oxidoreductase protein
orf07408	863	25	0,80	hypothetical protein RPA2820
scal00594	864	162	0,80	amtB ammonium transport protein
scal02399	865	143	0,80	similar to dihydroorotase
scal00460	866	61	0,80	No hits found
scal03570	867	51	0,80	protein of unknown function UPF0027
scal02265	868	99	0,80	phosphoribosylaminoimidazole-succinocarboxamide (SAICAR) synthase
scal02117	869	173	0,80	putative FAD containing glycolate oxidase subunit GlcD
scal00563	870	157	0,80	glycine dehydrogenase subunit 2 GcvP
scal00843	871	53	0,80	General secretion pathway protein G
scal03554	872	137	0,80	gamma-glutamyl phosphate reductase
scal01190	873	68	0,80	putative beta lactamase / hydroxyacylglutathione hydrolase
scal00945	874	94	0,78	dihydrodipicolinate reductase
scal02239	875	179	0,78	putative Zn-dependent protease
scal01252	876	125	0,78	galactose-1-phosphate uridyltransferase
scal01699	877	296	0,78	hypothetical FAD oxidoreductase protein

scal00210	878	80	0,78	DnaJ like Heat shock protein
scal03795	879	79	0,78	similar to ribonuclease III
scal03910	880	113	0,78	4-hydroxy-3-methylbut-2-en-1-yl diphosphate synthase
scal02942	881	79	0,78	peptidase M23B
scal03839	882	100	0,78	3-deoxy-D-arabino-heptulosonate 7-phosphate synthase
scal02072	883	80	0,78	similar to GMP synthase
scal03497	884	194	0,78	Pyruvate ferredoxin oxidoreductase porA
scal02132	885	302	0,78	phosphoenolpyruvate synthase/ pyruvate phosphate dikinase
scal00887	886	53	0,78	hypothetical protein
scal03168	887	208	0,78	Methyltransferase type 11
scal01729	888	57	0,78	Putative translation initiation inhibitor
scal03690	889	55	0,78	Glyoxalase/bleomycin resistance protein/dioxygenase
scal00553	890	41	0,78	mannose-6-phosphate isomerase, type II
scal04107	891	73	0,78	ExsB transcriptional regulator
scal01211	892	56	0,78	YDR077W
orf00699	893	24	0,78	Uncharacterized protein family UPF0033
orf06726	894	27	0,78	No hits found
scal02662	895	358	0,78	carbamoyl-phosphate synthase large subunit
scal04242	896	171	0,78	putative glucose-6-phosphate 1-dehydrogenase
scal00316	897	71	0,78	conserved hypothetical protein
scal02392	898	43	0,75	strongly similar to aspartate 1-decarboxylase precursor (aspartate alpha-decarboxylase).
scal02534	899	173	0,75	putative amino acid transporter
scal02038	900	33	0,75	No hits found
scal03418	901	111	0,75	hypothetical protein
scal02331	902	66	0,75	putative iron sulfur heterodisulfide reductase subunit
scal03481	903	111	0,75	Glutathione synthase
scal01950	904	147	0,75	putative response regulator
scal00518	905	75	0,75	unknown protein
scal02938	906	40	0,75	Uncharacterised P-loop hydrolase UPF0079
scal03980	907	79	0,75	similar to ATPase component of glycine betaine ABC type transporter
scal00181	908	110	0,75	putative ankyrin repeat protein
scal01439	909	175	0,75	Phosphomannomutase
scal02327	910	111	0,75	conserved hypothetical protein
scal00499	911	24	0,75	conserved hypothetical duf433 protein
scal00921	912	127	0,75	strongly similar to competence protein DprA
scal01668	913	368	0,75	hypothetical protein CY0110_30026
scal00192	914	62	0,75	expressed putative thiJ thiazole monophosphate biosynthesis protein
scal02023	915	138	0,75	DnaA replication initiation protein
scal00880	916	477	0,75	large tpr repeat protein
scal02315	917	93	0,75	GTP-binding protein Era
scal03631	918	111	0,75	aminotransferase, class V
scal03987	919	129	0,75	iron-containing alcohol dehydrogenase
orf03865	920	126	0,75	similar to ATP phosphoribosyltransferase
scal02859	921	226	0,73	recQ ATP-dependent DNA helicase
scal00829	922	55	0,73	No hits found
scal02014	923	73	0,73	expressed conserved hypothetical protein
scal01033	924	66	0,73	hypothetical protein
scal01596	925	36	0,73	putative tonB receptor protein
scal03148	926	30	0,73	unknown protein

scal00891	927	111	0,73	sorbitol dehydrogenase
scal01051	928	54	0,73	putative molybopterin cofactor biosynthesis protein mobA
scal00689	929	151	0,73	putative diheme protein
scal00847	930	131	0,73	unknown protein
orf01303	931	23	0,73	conserved hypothetical protein
scal03353	932	101	0,73	similar to ATPG gene encoding subunit gamma of ATP synthase
scal00954	933	33	0,73	cytochrome c-552 ks_3357
scal02150	934	89	0,73	ATP phosphoribosyltransferase
scal00272	935	165	0,73	expressed D-3-phosphoglycerate dehydrogenase
scal03699	936	39	0,73	conserved hypothetical protein
scal03905	937	105	0,73	hypothetical protein
scal02854	938	59	0,73	anthranilate synthase component II pabA
scal01228	939	63	0,73	hypothetical protein PM8797T_02964
scal03212	940	96	0,73	methyltransferase
scal03516	941	121	0,73	nucleoside diphosphate kinase 4
scal00611	942	80	0,73	PREDICTED: hypothetical protein
scal00551	943	79	0,73	conserved hypothetical protein
scal02339	944	30	0,73	No hits found
scal00072	945	171	0,73	expressed S-adenosyl-L-homocysteine hydrolase
scal02417	946	181	0,73	pyruvate carboxylase, beta chain
scal02828	947	128	0,73	strongly similar to tyrosyl-tRNA ligase
scal04018	948	65	0,73	conserved hypothetical protein
scal02912	949	34	0,73	No hits found
scal01082	950	140	0,73	hypothetical protein
scal00364	951	79	0,71	conserved hypothetical protein
scal03297	952	47	0,71	rrf2 family protein
scal03595	953	114	0,71	PAS/PAC sensor hybrid histidine kinase
scal02815	954	91	0,71	conserved hypothetical protein
scal04146	955	116	0,71	succinyl-CoA synthetase, beta subunit
scal02151	956	154	0,71	putative TPR repeat protein; N-acetylglucosaminyl transferase
scal03498	957	78	0,71	pyruvate ferredoxin oxidoreductase porB
scal00855	958	207	0,71	similar to general secretion pathway protein D
scal03452	959	31	0,71	putative plasmid maintenance protein HigB with helix turn helix motiv
scal00840	960	149	0,71	putative histidine kinase
scal00740	961	128	0,71	adenylosuccinate synthetase
scal02951	962	48	0,71	conserved hypothetical protein
scal04080	963	62	0,71	thiopurine S-methyltransferase family protein
scal00934	964	147	0,71	two component, sigma54 specific, transcriptional regulator, Fis family
scal04227	965	54	0,71	strongly sinmilar to dTDP-4-deoxyrhamnose 3,5 epimerase
scal03195	966	69	0,71	unknown protein
scal00225	967	126	0,71	glycinamide ribonucleotide synthetase
scal01464	968	100	0,71	putative Zinc-containing alcohol dehydrogenase
scal00695	969	101	0,71	hypothetical TIM barrel protein
scal00440	970	108	0,71	expressed UDP-N-acetylglucosamine acyltransferase LpxA
scal02762	971	106	0,71	Porphyromonas-type peptidyl-arginine deiminase
scal02388	972	352	0,71	putative M28 peptidase
scal02235	973	49	0,71	similar to heme d1 synthesis protein nirH/nirL
scal03300	974	120	0,71	aspartate aminotransferase AspC
scal02844	975	85	0,71	hypothetical protein Pcar_2615

scal00359	976	78	0,71	porB pyruvate:ferredoxin and related 2-oxoacid:ferredoxin oxidoreductases, beta subunit
scal00721	977	109	0,71	conserved hypothetical protein
scal01955	978	70	0,71	hypothetical protein
scal01179	979	131	0,71	hypothetical methyltransferase
scal00748	980	120	0,71	putative flavopotein norVW
scal03487	981	92	0,71	similar to two component histidine kinase
scal02289	982	308	0,68	sodium/potassium proton antiport protein GerN
scal01944	983	189	0,68	similar to chemotaxis histidine kinase
scal03696	984	162	0,68	hypothetical protein with PilF (COG3063) domain
scal01637	985	33	0,68	similar to to proton-translocating NADH dehydrogenase I chain A (NuoA)
scal04041	986	134	0,68	similar to beta-ketoacyl acyl carrier protein synthase II
scal02660	987	120	0,68	3-deoxy-D-manno-octulosonic acid transferase
scal02135	988	259	0,68	putative qnor nitric oxide reductase
scal03552	989	51	0,68	hypothetical protein Gura_1746
orf00430	990	20	0,68	hypothetical protein Cyan7425_3371
orf00759	991	16	0,68	putative rubredoxin
scal00459	992	44	0,68	putative FMN containing pyridoxamine 5'-phosphate oxidase
orf00773	993	30	0,68	pentapeptide repeat-containing protein
orf04910	994	15	0,68	No hits found
scal01313	995	172	0,68	partial hypothetical bc fusion protein
scal00313	996	66	0,68	putative lipoprotein
scal01163	997	325	0,68	smc chromosome partition ATPase
orf00928	998	23	0,68	putative Sec-independent protein translocase component
scal02463	999	81	0,68	sulfate adenylyltransferase, small subunit
scal00199	1000	56	0,68	expressed delta subunit of F0F1 ATP synthase
scal03790	1001	56	0,68	putative integral membrane component of sec protein-translocation machinery, secG
scal03623	1002	49	0,68	DEAD/DEAH box helicase-like protein
scal01246	1003	170	0,68	unknown protein
scal02401	1004	114	0,68	conserved hypothetical protein
orf06773	1005	24	0,68	GIY-YIG catalytic domain protein
scal02883	1006	101	0,68	biotin synthase
scal00654	1007	115	0,68	putative proteinase
scal00236	1008	169	0,68	similar to DNA primase
scal00400	1009	127	0,68	similar to potassium uptake protein TrkA
orf06832	1010	13	0,68	No hits found
scal02734	1011	104	0,68	strongly similar to 2-amino-3-ketobutyrate CoA ligase
scal01820	1012	241	0,66	SNF2-related:Helicase-like:SWIM Zn-finger:DEAD/DEAH box helicase-like
scal00347	1013	104	0,66	conserved hypothetical protein
scal03674	1014	33	0,66	transposase, IS4 family protein
scal01215	1015	80	0,66	similar to putative signal peptidase protein
scal04037	1016	56	0,66	unknown protein
scal03968	1017	29	0,66	No hits found
scal00231	1018	102	0,66	conserved hypothetical protein
scal04109	1019	112	0,66	putative atz/trz chlorohydrolase/deaminase protein
scal02613	1020	50	0,66	conserved hypothetical protein
scal03979	1021	169	0,66	hypothetical methyl-accepting protein
scal02118	1022	111	0,66	putative iron sulfur glycolate oxidase protein
scal00707	1023	288	0,66	AcrB, putative cation/multidrug efflux pump
scal03511	1024	151	0,66	Carbamoyl transferase

scal02270	1025	58	0,66	strongly similar to thiamin phosphate pyrophosphorylase (thiamin phosphate synthase)
scal02743	1026	200	0,66	putative napA like FeS molybdopterin oxidoreductase C terminal
scal02893	1027	162	0,66	conserved hypothetical GTP-binding protein
scal00716	1028	48	0,66	conserved hypothetical protein
scal03472	1029	129	0,66	pyruvate kinase
scal01900	1030	73	0,66	putative flagellar basal-body rod protein
scal02948	1031	72	0,66	conserved hypothetical protein
scal02328	1032	89	0,66	conserved hypothetical protein
scal03072	1033	136	0,66	putative ABC transporter ATP binding protein
orf03597	1034	19	0,66	unknown protein
scal01274	1035	71	0,66	general secretion pathway protein-related protein
scal01032	1036	125	0,66	AAA ATPase
scal02978	1037	116	0,66	Protease
scal03773	1038	32	0,66	conserved hypothetical protein
scal03237	1039	43	0,66	putative phosphoesterase
scal02646	1040	171	0,66	V-type ATPase subunit A
scal04190	1041	96	0,66	pseudouridine synthase
scal01287	1042	85	0,64	5,10-methylenetetrahydrofolate reductase
scal03311	1043	123	0,64	hypothetical protein ST0815
scal01219	1044	104	0,64	strongly similar to serine protease
scal02185	1045	95	0,64	putative methyl-accepting chemotaxis protein
scal00312	1046	74	0,64	hypothetical protein P700755_01292
scal02739	1047	47	0,64	unknown protein
scal01000	1048	118	0,64	unknown protein
scal03571	1049	78	0,64	nucleic acid binding protein
scal02551	1050	38	0,64	unknown protein
scal01267	1051	30	0,64	No hits found
scal00711	1052	33	0,64	conserved hypothetical protein containing Rhodanese Homology Domain (RHOD)
scal04162	1053	36	0,64	hypothetical protein MELB17_13282
scal04180	1054	40	0,64	No hits found
scal00285	1055	90	0,64	putative ABC transporter ATP binding protein
scal01557	1056	83	0,64	nuoG or FDH like molybdopterin containing oxidoreductase
scal00844	1057	151	0,64	strongly similar to general secretion pathway protein E
scal02288	1058	145	0,64	putative multiheme cytochrome c protein with 8 heme / cxxch motives
scal00191	1059	65	0,64	hypothetical duf1264 lipoprotein
orf03987	1060	44	0,64	similar to heme d1 synthesis protein nirH/nirL
scal02667	1061	32	0,64	No hits found
scal00774	1062	35	0,64	No hits found
scal00904	1063	86	0,64	No hits found
scal01610	1064	52	0,64	putative solvent tolerance protein
orf05350	1065	126	0,64	fad-dependent pyridine nucleotide-disulfide oxidoreductase
scal01141	1066	138	0,64	diguanylate cyclase
scal00640	1067	108	0,64	putative acetyl-CoA acetyltransferase; thiolase
scal01652	1068	144	0,64	ABC transporter-related protein
scal00427	1069	77	0,64	putative cell shape-determining protein MreC
scal01511	1070	110	0,64	Xaa-Pro aminopeptidase
scal00480	1071	26	0,64	No hits found
scal02246	1072	138	0,64	ADP-heptose synthase
scal00365	1073	44	0,64	strongly similar to phosphopantetheine adenylyltransferase (PPAT)

scal04252	1074	42	0,64	sensory box protein/sigma-54 dependent DNA-binding response regulator
scal03012	1075	108	0,64	putative membrane efflux protein
scal03069	1076	40	0,64	universal stress protein uspA
scal02886	1077	55	0,64	strongly similar to dephosphocoenzyme A kinase
scal03219	1078	115	0,64	hypothetical protein
scal03479	1079	175	0,64	conserved hypothetical protein
scal01378	1080	113	0,64	hypothetical protein
scal00144	1081	167	0,64	putative peptidase U32
scal02174	1082	56	0,64	putative outer membrane chaperone OmpH
scal02888	1083	157	0,62	similar to putative glucose inhibited division protein A
scal00488	1084	96	0,62	strongly similar to class I peptide chain release factor
orf04001	1085	15	0,62	similar to UDP-glucose 4-epimerase
scal00123	1086	73	0,62	conserved hypothetical protein
scal01607	1087	68	0,62	strongly similar to ABC-transporter, permease
scal00638	1088	122	0,62	putative 3-hydroxy-3-methylglutaryl-coenzyme A reductase
scal00856	1089	31	0,62	hypothetical protein PM8797T_31598
scal03675	1090	30	0,62	No hits found
scal03926	1091	59	0,62	putative protein-L-isoaspartate O-methyltransferase
scal00005	1092	224	0,62	Methyltransferase type 11
scal03789	1093	76	0,62	putative stress protein
scal03333	1094	107	0,62	pyridoxal phosphate-dependent enzyme apparently involved in regulation of cell wall biogenesis
scal01546	1095	59	0,62	similar to phosphatidylserine decarboxylase proenzyme
scal02505	1096	45	0,62	conserved hypothetical protein
scal03827	1097	68	0,62	hypothetical DUF558 protein
scal02802	1098	101	0,62	unknown protein
scal02088	1099	263	0,62	similar to Phosphoribosylformylglycinamidine synthase II
scal00578	1100	49	0,62	hypothetical protein VNG0742H
scal01648	1101	165	0,62	strongly similar to NAD(P)H:quinone oxidoreductase chain 5
scal03217	1102	103	0,62	unknown protein
scal02636	1103	53	0,62	unknown protein part of the hydrazine synthase cluster
scal02082	1104	174	0,62	TPR Domain containing protein
scal00494	1105	22	0,62	unknown protein
scal02006	1106	104	0,62	glycosyl transferase, group 1
scal02610	1107	80	0,62	similar to tetracenomycin polyketide synthesis 8-O-methyltransferase
scal02332	1108	87	0,62	putative heterodisulfide reductase subunit B
scal01070	1109	139	0,62	PEGA
scal02606	1110	91	0,62	histidinol-phosphate aminotransferase
scal03213	1111	159	0,62	unknown protein
scal03844	1112	104	0,62	hypothetical protein
scal03307	1113	73	0,62	similar to acid phosphatase SurE
scal02809	1114	71	0,62	6-phosphogluconolactonase
scal02094	1115	74	0,62	type IV prepilin-like protein leader peptide processing enzyme
orf04690	1116	19	0,62	No hits found
scal03301	1117	25	0,62	hypothetical protein
scal00449	1118	63	0,62	putative ABC transporter protein
scal04152	1119	62	0,62	conserved hypothetical protein
scal02340	1120	146	0,62	predicted orf
scal00457	1121	29	0,62	putative iron sulfur protein
scal01356	1122	121	0,62	conserved hypothetical protein

scal03629	1123	42	0,62	Sulfopyruvate decarboxylase
scal01150	1124	130	0,62	possible carbon-nitrogen hydrolase
scal00850	1125	36	0,62	No hits found
scal01188	1126	77	0,62	probable type II secretion system protein
scal02244	1127	51	0,62	UDP-glucose 4-epimerase
scal00511	1128	102	0,59	similar to Na(+)-translocating NADH-quinone reductase subunit B
scal02614	1129	151	0,59	strongly similar to glucosamine-fructose-6-phosphate aminotransferase
scal03921	1130	75	0,59	putative TPR repeat protein
scal01728	1131	144	0,59	indole-3-pyruvate decarboxylase
scal02801	1132	25	0,59	expresse conserved protein COG2331
scal03746	1133	91	0,59	Glycine cleavage system T protein (aminomethyltransferase)
scal02323	1134	131	0,59	Predicted Fe-S oxidoreductase
scal04160	1135	199	0,59	isocitrate dehydrogenase, NADP-dependent
scal01836	1136	86	0,59	conserved hypothetical signal transduction HD GYP protein
scal02007	1137	52	0,59	hypothetical protein Mbar_A1486
scal03816	1138	85	0,59	Predicted dehydrogenase related to phosphoglycerate dehydrogenase
scal01547	1139	81	0,59	No hits found
scal00224	1140	66	0,59	unknown protein
scal04310	1141	15	0,59	conserved protein of unknown function
scal03878	1142	119	0,59	hypothetical protein
scal02495	1143	49	0,59	conserved hypothetical cog1611 protein
scal01400	1144	47	0,59	peptidyl-prolyl cis-trans isomerase A
scal00688	1145	174	0,59	putative cytochrome bd quinol oxidase (cydA) of nar cluster
scal01229	1146	140	0,59	ferredoxin-dependent glutamate synthase
scal01614	1147	109	0,59	Mg/Co/Ni transporter MgtE (contains CBS domain)
scal00730	1148	62	0,59	strongly similar to pyridoxal phosphate biosynthetic protein PdxJ (PNP synthase)
scal01525	1149	97	0,59	putative efflux protein
scal00222	1150	80	0,59	expressed spermidine synthase (putrescine aminopropyltransferase)
orf05747	1151	11	0,59	conserved hypothetical protein
scal03427	1152	244	0,59	DnaK-related protein
scal02698	1153	48	0,59	hypothetical protein
scal00974	1154	81	0,59	focA formate/nitrite transport protein
scal03541	1155	41	0,59	similar to molybdenum cofactor biosynthesis protein B
scal01606	1156	64	0,59	putative phosphoketolase
scal00738	1157	65	0,59	CDP-diglyceride synthetase
scal02846	1158	91	0,59	putative NAD 2-hydroxyacid dehydrogenase
scal03184	1159	119	0,59	anthranilate synthase
scal03234	1160	68	0,59	similar to enoyl-CoA hydratase
scal00535	1161	51	0,59	similar to YrdC protein
scal01947	1162	32	0,59	putative response regulator
orf00637	1163	22	0,59	hypothetical protein
scal02304	1164	46	0,59	hypothetical protein LNTAR_12331
orf05634	1165	14	0,59	No hits found
scal01099	1166	49	0,59	putative outer membrane chaperone OmpH
scal03866	1167	43	0,59	hypothetical protein Bcenmc03DRAFT_3311
scal00174	1168	273	0,59	AcrB/AcrD/AcrF family protein
scal03664	1169	89	0,59	two component, sigma54 specific, transcriptional regulator, Fis family
scal00140	1170	80	0,57	1-aminocyclopropane-1-carboxylate deaminase
scal00448	1171	107	0,57	AcrA efflux protein

scal03239	1172	23	0,57	No hits found
scal01560	1173	40	0,57	putative cytochrome c CII protein
scal02309	1174	70	0,57	metallo-beta-lactamase superfamily protein
scal02852	1175	107	0,57	glutamyl-tRNA(Gln) amidotransferase, B subunit
scal03035	1176	49	0,57	cbb3-type cytochrome c oxidase subunit CcoP
scal03605	1177	78	0,57	GDP-mannose 4,6-dehydratase
scal02664	1178	44	0,57	putative CDP-diacylglycerol--glycerol-3-phosphate 3-phosphatidyltransferase
scal03645	1179	96	0,57	NAD(P)H glycerol-3-phosphate dehydrogenase GpdA
scal00606	1180	49	0,57	ErfK/YbiS/YcfS/YnhG family protein
scal03074	1181	78	0,57	serine acetyltransferase, plasmid
scal02314	1182	29	0,57	No hits found
scal02026	1183	168	0,57	similar to glycine-tRNA ligase (beta-chain)
scal02748	1184	45	0,57	secreted protein
scal00227	1185	66	0,57	similar to VacJ lipoprotein
scal01450	1186	78	0,57	ABC transporter related
scal01335	1187	191	0,57	copper-translocating P-type ATPase
scal00788	1188	111	0,57	putative ABC transport protein
scal00846	1189	96	0,57	conserved hypothetical protein
scal01659	1190	97	0,57	type II secretion system protein
scal03079	1191	57	0,57	similar to potassium uptake system protein TrkA
scal01948	1192	29	0,57	anti-sigma-28 factor, FlgM
scal02460	1193	26	0,57	putative transposase
scal02129	1194	59	0,57	hypothetical protein with 1 cxxch motive
scal03719	1195	128	0,57	lipopolysaccharide biosynthesis
scal02988	1196	78	0,57	putative molybdopterin synthesis protein MoeB and thiamin biosynthesis protein ThiF
scal00094	1197	182	0,57	expressed protein with unknown function
scal01271	1198	210	0,57	uncharacterized exopolysaccharide biosynthesis protein
scal00358	1199	47	0,57	porC pyruvate:ferredoxin and related 2-oxoacid:ferredoxin oxidoreductases, gamma subunit
scal03850	1200	127	0,57	DNA topoisomerase VI, B subunit
scal01324	1201	37	0,57	transcription antitermination protein NusG
scal00573	1202	39	0,57	hypothetical protein PI23P_08010
scal03211	1203	59	0,57	Type II site-specific deoxyribonuclease
scal02806	1204	79	0,57	conserved hypothetical protein
scal03837	1205	30	0,57	glycoside hydrolase, family 13-like
scal03425	1206	28	0,57	No hits found
scal03879	1207	62	0,57	hypothetical protein STH1698
scal03745	1208	200	0,57	Leucyl-tRNA synthetase bacterial/mitochondrial, class Ia
scal03224	1209	225	0,57	clumping factor A
scal01613	1210	183	0,57	glycogen phosphorylase
scal02287	1211	94	0,57	conserved hypothetical protein
scal04145	1212	73	0,57	succinyl-CoA synthetase, alpha subunit
scal01724	1213	60	0,57	No hits found
scal03849	1214	58	0,57	DNA topoisomerase VI, subunit A
scal00530	1215	81	0,57	magnesium and cobalt transport protein CorA
scal01833	1216	228	0,57	unknown protein
scal02917	1217	81	0,57	hypothetical protein
scal00233	1218	44	0,57	unknown protein
orf01625	1219	21	0,57	unknown protein
scal03845	1220	86	0,57	strongly similar to tRNA-guanine transglycosylase

scal04067	1221	80	0,57	NAD-dependent epimerase/dehydratase
scal01572	1222	27	0,57	hypothetical protein Dace_0111
scal01903	1223	95	0,57	flagellar P-ring protein
scal01653	1224	53	0,55	pseudouridine synthase
scal02140	1225	42	0,55	hypothetical protein SPV1_08381
scal02025	1226	69	0,55	recombinase
scal02389	1227	92	0,55	WD40-like Beta Propeller
orf01492	1228	13	0,55	No hits found
scal03998	1229	166	0,55	oligopeptide ABC transport protein
scal03532	1230	110	0,55	DNA-dependent ATPase DNA helicase (RecQ)
scal01887	1231	23	0,55	No hits found
scal01173	1232	62	0,55	cobalamin biosynthesis protein CbiG
scal02418	1233	125	0,55	Na+-transporting methylmalonyl-CoA/oxaloacetate decarboxylase, beta subunit
scal00241	1234	125	0,55	strongly similar to CTP synthase (UTP-ammonia ligase)
scal04023	1235	137	0,55	similar to ribonuclease R
scal03601	1236	100	0,55	strongly similar to aspartokinase
scal03013	1237	374	0,55	putative efflux transporter
scal01633	1238	90	0,55	DegT/DnrJ/EryC1/StrS aminotransferase
scal01489	1239	105	0,55	hypothetical protein MJ1479
scal03737	1240	183	0,55	glycoside hydrolase, family 57
orf06496	1241	26	0,55	nicotinamide nucleotide transhydrogenase, subunit alpha
scal03630	1242	45	0,55	thiamine pyrophosphate enzyme domain protein TPP-binding
scal01072	1243	29	0,55	putative response regulator protein
scal03738	1244	120	0,55	strongly similar to 4-alpha-glucanotransferase
scal00087	1245	31	0,55	similar to methylmalonyl-CoA epimerase
scal02904	1246	85	0,55	dihydroorotate oxidase catalytic subunit
scal03650	1247	27	0,55	50S ribosomal protein L20
scal02254	1248	38	0,55	No hits found
scal02800	1249	51	0,55	putative molecular chaperone protein grpE
scal04295	1250	42	0,55	putative cytochrome c CII protein
scal02324	1251	64	0,55	unknown protein
scal01883	1252	42	0,55	No hits found
scal03726	1253	50	0,55	putative sensor protein
scal00083	1254	169	0,55	urocanate hydratase
scal03984	1255	44	0,55	unknown protein
scal03150	1256	95	0,55	putative deoxyribodipyrimidine photolyase
scal03461	1257	55	0,55	Abortive infection protein
scal00773	1258	45	0,55	peptidyl-prolyl cis-trans isomerase, FKBP-type
scal01457	1259	34	0,55	probable cyclic nucleotide binding protein
scal00996	1260	56	0,55	strongly similar to indole-3-glycerol phosphate synthase (IGPS)
scal00344	1261	50	0,55	conserved hypothetical protein
scal02891	1262	56	0,55	similar to chain A of ATP binding cassette transporter
scal03540	1263	33	0,55	conserved hypothetical protein
scal02907	1264	90	0,55	anthranilate phosphoribosyltransferase
scal01541	1265	70	0,52	similar to dimethyladenosine transferase KsgA
scal02163	1266	42	0,52	unknown anammox protein
scal02093	1267	138	0,52	exonuclease RecJ
scal00739	1268	52	0,52	undecaprenyl diphosphate synthase
scal02024	1269	110	0,52	phosphomannomutase

scal04140	1270	98	0,52	phosphoesterase
scal01057	1271	73	0,52	RecJ like Phosphoesterase
scal00600	1272	83	0,52	putative Phosphatidylserine/phosphatidylglycerophosphate synthase
scal02148	1273	33	0,52	putative cytochrome c CII protein
scal00345	1274	24	0,52	similar to molybdopterin synthase subunit 1
scal02577	1275	36	0,52	similar to type II lipoprotein signal peptidase
scal02625	1276	79	0,52	hypothetical protein
scal04040	1277	113	0,52	similar to phytoene dehydrogenase (phytoene desaturase)
scal03736	1278	149	0,52	strongly similar to 1,4-alpha-glucan branching enzyme
scal01449	1279	25	0,52	conserved hypothetical protein
scal01177	1280	45	0,52	hypothetical protein
scal02032	1281	63	0,52	putative cobalt transport ATP-binding protein CbiO
scal03793	1282	79	0,52	strongly similar to chorismate mutase / prephenate dehydratase
scal00932	1283	28	0,52	hypothetical protein MED92_02279
scal00013	1284	59	0,52	putative expressed iron sulfur flavoprotein
scal00124	1285	51	0,52	molybdate ABC transporter, inner membrane subunit
scal03474	1286	220	0,52	No hits found
scal03976	1287	37	0,52	hypothetical protein Plav_0759
scal02675	1288	90	0,52	hypothetical upf0075 protein
scal02952	1289	123	0,52	hypothetical protein PTH_2728
scal00281	1290	52	0,52	putative plastocyanine containing petE protein
scal02607	1291	23	0,52	No hits found
scal00940	1292	48	0,52	strongly similar to N-acetylmuramoyl-L-alanine amidase (T7 lysozyme)
scal03206	1293	78	0,52	similar to histidinol phosphate aminotransferase
scal01340	1294	76	0,52	ribokinase protein rbsK
scal01172	1295	59	0,52	precorrin-3B C17-methyltransferase
scal01288	1296	55	0,52	cAMP-dependent transcriptional regulator
scal00078	1297	72	0,52	expressed protein with unknown function
scal04102	1298	119	0,52	ATP-dependent RNA helicase
scal01235	1299	51	0,52	similar to bifunctional protein Bir A
scal01071	1300	206	0,52	GAF sensor signal transduction histidine kinase
scal04083	1301	63	0,52	conserved hypothetical protein
scal00503	1302	22	0,52	putativen plasmid maintenance protein HigA
scal03922	1303	59	0,52	putative tpr repeat pilF like protein
scal02587	1304	69	0,52	aldo/keto reductase
scal02438	1305	157	0,52	similar to flagellar hook protein
scal01383	1306	45	0,52	GCN5-related N-acetyltransferase
scal00756	1307	119	0,52	putative integral membrane transport protein
scal00668	1308	173	0,52	strongly similar to ATP-dependent protease La
scal00360	1309	76	0,52	expressed porA pyruvate:ferredoxin and related 2-oxoacid:ferredoxin oxidoreductases
scal02223	1310	60	0,52	conserved hypothetical protein
scal00534	1311	33	0,52	expressed putative ribose-5-phosphate isomerase rpiB
scal02348	1312	69	0,52	nicotinate-nucleotide pyrophosphorylase
scal00862	1313	123	0,52	hypothetical protein
scal03022	1314	43	0,52	NADPH-dependent FMN reductase
scal03175	1315	29	0,52	putative copper-transporting P-type ATPase
scal03615	1316	29	0,52	putative biotin synthase / SAM radical protein
scal04042	1317	88	0,52	beta-ketoacyl acyl carrier protein synthase II
scal00080	1318	39	0,52	unkown protein

scal00398	1319	93	0,50	similar to ATP-dependent protease Lon
scal02897	1320	66	0,50	ornithine carbamoyltransferase
scal03075	1321	72	0,50	cysteine synthase
scal01620	1322	106	0,50	similar to UDP-N-acetylmuramoylalanine D-glutamate ligase
scal00322	1323	17	0,50	unknown protein
scal01870	1324	34	0,50	phosphatidylglycerophosphatase
scal03781	1325	57	0,50	cobalamin biosynthesis precorrin-3 methylase (cbiF)
scal01665	1326	135	0,50	type IV pilus biogenesis protein PilM
scal04044	1327	51	0,50	chromosome partitioning protein ParA
scal00305	1328	70	0,50	hypothetical protein MA1656
scal02196	1329	135	0,50	hypothetical FAD oxidoreductase protein
scal01540	1330	42	0,50	serine/threonine protein kinase
scal04282	1331	85	0,50	hypothetical protein
scal03478	1332	55	0,50	conserved hypothetical protein
scal02834	1333	73	0,50	strongly similar to N-acetyl-gamma-glutamyl-phosphate reductase (NAGSA dehydrogenase)
scal03332	1334	56	0,50	Methyltransferase type 11
scal00552	1335	80	0,50	Pyruvate formate-lyase activating enzyme-like protein
scal02290	1336	145	0,50	hypothetical protein
orf05525	1337	12	0,50	No hits found
scal03451	1338	18	0,50	putativen plasmid maintenance protein HigA
scal04055	1339	59	0,50	hypothetical protein
scal02125	1340	111	0,50	phosphoenolpyruvate phosphomutase
scal03233	1341	159	0,50	2,4-dieonyl-CoA reductase, FMN-linked partail see 744509
scal03207	1342	44	0,50	strongly similar to imidazoleglycerol-phosphate dehydratase
scal01745	1343	55	0,50	hypothetical protein Tery_3474
scal00143	1344	89	0,50	conserved hypothetical protein
scal04064	1345	88	0,50	hypothetical protein
scal00886	1346	101	0,50	hypothetical protein
orf06792	1347	23	0,50	Xylose isomerase domain protein TIM barrel
scal02627	1348	73	0,50	hypothetical protein
scal03114	1349	45	0,50	conserved hypothetical protein
scal02083	1350	46	0,50	similar to phosphoribosylglycinamide formyltransferase
orf05026	1351	16	0,50	No hits found
scal02612	1352	72	0,50	strongly similar to UDP-3-O-[3-hydroxymyristoyl] glucosamine N-acetyltransfrase
scal00007	1353	74	0,50	hypothetical protein NmarDRAFT_0695
scal00017	1354	44	0,50	hypothetical protein L8106_02057
scal01147	1355	90	0,50	strongly similar to arginine biosynthesis bifunctional protein
scal03770	1356	47	0,50	unknown protein
scal02571	1357	54	0,50	hypothetical protein
scal01291	1358	78	0,50	glycosyl transferase, group 1
scal02837	1359	27	0,50	No hits found
scal04283	1360	113	0,50	strongly similar to transcription initiation factor sigma RpoD
scal02458	1361	50	0,50	similar to 1-acyl-sn-glycerol-3-phosphate acyltransferase (1-AGPacyltransferase) (1-AGPAT) (lysophosphatidic acid acyltransferase) (LPAAT).
scal00762	1362	35	0,50	RNase HI
scal02360	1363	177	0,50	DNA mismatch repair protein MutS see also
scal01622	1364	123	0,50	similar to membrane associated lipoprotein involved in thiamine biosynthesis apbE
scal02792	1365	35	0,50	conserved hypothetical protein
scal02939	1366	22	0,50	hypothetical protein protein
scal01278	1367	163	0,50	adenylate cyclase

scal00022	1368	41	0,50	D-glycero-D-manno-heptose-1-phosphate adenylyltransferase
scal00930	1369	62	0,50	hypothetical protein MED92_02269
scal00051	1370	47	0,50	ribosomal small subunit pseudouridine synthase A
scal00629	1371	60	0,50	resC/ccsA type II cytochrome c biogenesis protein
scal00727	1372	112	0,50	putative outer membrane efflux protein containing TolC domain
scal02021	1373	94	0,50	UDP-glucose 6-dehydrogenase
scal02018	1374	79	0,50	conserved hypothetical protein
scal02811	1375	109	0,48	glucose-6-phosphate 1-dehydrogenase
scal01847	1376	77	0,48	kusta0010 MltA;similar to membrane-bound lytic murein transglycosylase A
scal01469	1377	26	0,48	hypothetical protein PI23P_08020
scal03027	1378	119	0,48	thiamine pyrophosphate enzyme
scal01650	1379	105	0,48	similar to NAD(P)H-quinone oxidoreductase chain 2
scal04139	1380	188	0,48	hypothetical protein lwe2238
scal02310	1381	55	0,48	3-oxoacyl acyl-carrier protein reductase
scal03720	1382	61	0,48	general secretion pathway protein A
scal01300	1383	64	0,48	strongly similar to tryptophanyl-tRNA synthetase
scal00194	1384	103	0,48	argininosuccinate lyase argh
scal02215	1385	68	0,48	conserved hypothetical protein
scal04157	1386	58	0,48	TPR Domain containing protein
scal03982	1387	69	0,48	conserved hypothetical cog4301 protein
scal03506	1388	106	0,48	type III restriction enzyme, res subunit
orf06396	1389	17	0,48	predicted orf
orf04118	1390	18	0,48	ATPase AAA-2
scal01444	1391	26	0,48	conserved hypothetical protein
scal02741	1392	53	0,48	putative two component cog 2204 response regulator
scal00121	1393	62	0,48	putative biotin synthase / SAM radical protein
scal02177	1394	72	0,48	putative NAD dependent oxidoreductase
scal00147	1395	40	0,48	putative cytochrome b6
orf06236	1396	11	0,48	No hits found
orf04620	1397	64	0,48	aldo/keto reductase
scal02161	1398	111	0,48	putative PmbA/TldD protein modulator of dna gyrase
scal00082	1399	92	0,48	imidazolonepropionase
scal00381	1400	56	0,48	similar to ABC-transporter involved in LPS biosynthesis Wzm
scal00902	1401	60	0,48	conserved hypothetical protein
scal02561	1402	24	0,48	No hits found
scal00639	1403	60	0,48	expressed 3-hydroxybutyryl-CoA dehydrogenase
scal00512	1404	47	0,48	similar to Na(+)-translocating NADH-quinone reductase subunit C
scal00630	1405	121	0,48	expressed resB-like type II cytochrome maturation protein
scal00690	1406	63	0,48	putative monoheme protein
scal01660	1407	35	0,48	pilin, putative
orf05292	1408	14	0,48	protein of unknown function UPF0150
orf03447	1409	21	0,48	plasmid maintenance system killer
scal02020	1410	113	0,48	partial acetyl-coa synthetase, acetate coa ligase AMP forming
scal01884	1411	90	0,48	putative transposase for insertion sequence isrm17
scal00900	1412	40	0,48	conserved hypothetical protein
scal01405	1413	66	0,48	hypothetical protein PM8797T_11079
scal03423	1414	39	0,48	uncharacterized Zn-ribbon-containing protein involved in phosphonate metabolism
scal02576	1415	56	0,48	branched-chain amino acid transferase
scal00209	1416	53	0,48	hypothetical protein

scal02394	1417	39	0,48	unknown protein with 1 cxxch motive
scal00947	1418	23	0,48	sensory box protein/sigma-54 dependent DNA-binding response regulator
scal02659	1419	91	0,48	S-adenosylmethionine synthetase
scal02408	1420	101	0,48	strongly similar to L-aspartate oxidase (quinolinate synthetase B)
scal00214	1421	36	0,48	conserved hypothetical protein
scal00634	1422	96	0,48	adenylosuccinate lyase purB
scal02865	1423	63	0,48	conserved hypothetical protein
scal04214	1424	83	0,48	Peptidase M20D, amidohydrolase
scal03780	1425	93	0,48	hypothetical protein pc0099
scal00475	1426	174	0,48	strongly similar to glycogen phosphorylase
scal00825	1427	82	0,48	conserved hypothetical protein
scal02329	1428	62	0,48	hypothetical protein
orf07405	1429	28	0,48	No hits found
scal01103	1430	104	0,48	sensory box protein/response regulator
scal03171	1431	95	0,48	probable magnesium transporter
orf04801	1432	18	0,48	No hits found
scal03865	1433	70	0,48	delta-aminolevulinate dehydratase (porphobilinogen synthase)
scal03892	1434	94	0,46	putative beta lactamase
scal01245	1435	33	0,46	putative nitroreductase
scal00772	1436	28	0,46	No hits found
scal00853	1437	54	0,46	unknown protein
scal00306	1438	118	0,46	histone deacetylase superfamily
scal03653	1439	56	0,46	putative methyl-accepting protein
scal02201	1440	33	0,46	GCN5-related N-acetyltransferase
scal00841	1441	47	0,46	two-component response regulator
scal03707	1442	38	0,46	putative PAS/PAC sensor protein
scal03440	1443	38	0,46	hypothetical protein DSM3645_00010
scal03235	1444	50	0,46	3-oxoacyl acyl-carrier protein reductase
scal01153	1445	119	0,46	similar to glycerophosphodiester phosphodiesterase
scal04024	1446	64	0,46	check annotation in kust; putative metal dependent phosphohydrolase
scal01485	1447	42	0,46	AMP-dependent synthetase and ligase
scal00685	1448	110	0,46	putative heme d1 biosynthesis protein NirJ
scal04297	1449	52	0,46	conserved hypothetical protein of aldo-/ketoreductase family
scal02622	1450	65	0,46	conserved hypothetical protein similar to MoxR-like ATPase
scal02043	1451	87	0,46	UDP-N-acetylmuramoylalanyl-D-glutamyl-2,6-diaminopimelate-D-alanyl-D-alanine ligase
scal00103	1452	54	0,46	nickel-dependent hydrogenase, small subunit
scal04198	1453	49	0,46	similar to 2-heptaprenyl-1,4-naphthoquinone methyltransferase
scal03965	1454	67	0,46	UvrD/REP helicase
scal04244	1455	116	0,46	hypothetical protein TTE0034
scal04259	1456	72	0,46	hypothetical protein
scal01518	1457	23	0,46	conserved hypothetical protein
scal02369	1458	33	0,46	shikimate kinase
scal02422	1459	27	0,46	ferric uptake regulator protein
scal02045	1460	76	0,46	cell division protein FtsW
scal03703	1461	115	0,46	putative hybrid cluster protein
scal03230	1462	77	0,46	similar to 3-oxoacyl-[acyl-carrier-protein] synthase II
scal00314	1463	53	0,46	strongly similar to phosphomethylpyrimidine kinase
scal02306	1464	137	0,46	putative molybdopterin oxidoreductase
scal03210	1465	59	0,46	unknown protein

scal03362	1466	19	0,46	uncharacterized conserved coiled coil protein
scal04118	1467	108	0,46	prolyl-tRNA synthetase
scal01193	1468	34	0,46	strongly similar to ATP-dependent Hsl protease, peptidase subunit
scal00571	1469	47	0,46	iron sulfur protein
orf03435	1470	13	0,46	conserved hypothetical protein
scal02992	1471	19	0,46	strongly similar to divalent cation tolerance protein
scal03296	1472	90	0,46	anion transporter
scal02940	1473	116	0,46	DNA mismatch repair protein
scal03314	1474	77	0,46	flavohemoglobin
scal01260	1475	59	0,46	unknown
scal00848	1476	45	0,46	hypothetical protein
scal02839	1477	126	0,46	conserved hypothetical protein
scal02147	1478	28	0,46	putative cytochrome c CII protein
scal03817	1479	60	0,46	GGDEF domain
scal03559	1480	39	0,46	unknown protein
scal01090	1481	108	0,46	sulfate permease family protein
orf01051	1482	19	0,46	acylphosphatase
scal00878	1483	46	0,46	hypothetical protein Fjoh_4830
scal00406	1484	45	0,46	cobalamin 5'-phosphate synthase
scal01073	1485	22	0,46	No hits found
scal02827	1486	60	0,46	similar to peptidyl-prolyl cis-trans isomerase (survival protein surA precursor)
scal03172	1487	231	0,46	MscS Mechanosensitive ion channel
scal01251	1488	117	0,46	putative GAF sensor signal transduction histidine kinase
scal00259	1489	55	0,46	expressed TatD twin arginine family protein
scal02493	1490	36	0,46	hamp domain protein
orf05289	1491	16	0,46	No hits found
scal01121	1492	70	0,46	deoxyguanosinetriphosphate triphosphohydrolase-like protein
scal00927	1493	67	0,46	Radical SAM domain protein
scal00319	1494	17	0,46	hypothetical protein BGP_5512
scal01434	1495	51	0,46	1-acyl-sn-glycerol-3-phosphate acyltransferases
scal04228	1496	65	0,46	dTDP-glucose 4,6-dehydratase
scal03731	1497	79	0,46	Alanine dehydrogenase/PNT, C-terminal:Alanine dehydrogenase/PNT, N-terminal
scal00936	1498	20	0,46	No hits found
scal03848	1499	51	0,43	putative Peptidoglycan-binding protein LysM
scal02301	1500	76	0,43	putative serine threonine protein kinase
scal01832	1501	24	0,43	hypothetical protein
scal02076	1502	70	0,43	strongly similar to phosphonopyruvate decarboxylase
scal02016	1503	45	0,43	putative membrane protein
scal00718	1504	89	0,43	putative asparaginyl-tRNA synthetase
scal04201	1505	65	0,43	sensory box protein
scal01440	1506	97	0,43	Glu/Leu/Phe/Val dehydrogenase, C terminal
scal01581	1507	20	0,43	Carboxymuconolactone decarboxylase
scal02594	1508	49	0,43	UDP-N-acetylglucosamine acetyltransferase
scal00444	1509	44	0,43	unknown protein
scal01159	1510	85	0,43	putative trna modification GTPase
scal00706	1511	63	0,43	acrA cation efflux transport protein
orf06412	1512	17	0,43	DNA polymerase beta subunit
scal01113	1513	37	0,43	General secretion pathway protein G
orf06678	1514	18	0,43	strongly similar to small heat shock protein

scal03181	1515	90	0,43	hypothetical protein
scal01145	1516	105	0,43	sensory box protein/sigma-54 dependent DNA-binding response regulator
scal03821	1517	19	0,43	polysialic acid capsule expression protein
scal00361	1518	51	0,43	strongly similar to inorganic polyphosphate/ATP-NAD kinase
scal02414	1519	43	0,43	hypothetical protein NE1275
scal01175	1520	110	0,43	conserved hypothetical protein
scal03222	1521	51	0,43	hypothetical protein PM8797T_23681
scal02842	1522	54	0,43	GumN
scal00755	1523	99	0,43	SSS sodium solute transporter superfamily
orf05845	1524	14	0,43	No hits found
scal02559	1525	101	0,43	short-chain dehydrogenase
scal02919	1526	256	0,43	AcrB/AcrD/AcrF family protein
scal02737	1527	59	0,43	similar to dTDP-glucose 4,6-dehydratase
scal02585	1528	39	0,43	conserved hypothetical protein
scal02131	1529	136	0,43	putative tpr repeat protein
scal03688	1530	7	0,43	putative homoserine O-succinyltransferase
scal04059	1531	31	0,43	putative sterol carrier protein
scal00295	1532	48	0,43	unknown protein
scal02141	1533	74	0,43	phosphoserine phosphatase SerB
scal03881	1534	22	0,43	hypothetical protein MXAN_6597
scal01117	1535	44	0,43	No hits found
orf04359	1536	18	0,43	conserved hypothetical protein
scal00439	1537	69	0,43	hypothetical abortive infection protein
scal01673	1538	71	0,43	strongly similar to putative secretion protein PilU
scal02225	1539	110	0,43	amino acid carrier protein
scal01844	1540	42	0,43	pseudouridine synthase
scal02541	1541	59	0,43	malate dehydrogenase
scal01551	1542	64	0,43	predicted signal transduction protein containing a membrane domain, an EAL and a GGDEF domain
scal03791	1543	12	0,43	No hits found
scal01272	1544	111	0,43	TPR Domain containing protein
scal02697	1545	55	0,43	similar to site-specific tyrosine recombinase
scal00356	1546	20	0,43	hypothetical protein glr4040
scal01649	1547	98	0,43	similar to NADH:ubiquinone oxidoreductase subunit M
scal00258	1548	48	0,43	Xylose isomerase domain protein TIM barrel
scal02860	1549	87	0,43	strongly similar to dihydrolipoamide dehydrogenase of 2-oxoacid dehydrogenase (lipoamide) complex
scal01454	1550	29	0,43	hypothetical protein Dace_3035
scal03476	1551	107	0,43	trkA domain protein
scal02717	1552	138	0,43	ATP-dependent helicase, DinG family protein
scal01992	1553	42	0,43	hypothetical protein BT9727_2186
scal02921	1554	88	0,43	putative response regulator
scal01938	1555	67	0,43	strongly similar to chemotaxis response regulator protein (glutamate methylesterase)
scal00554	1556	98	0,43	similar to NAD synthase
scal01220	1557	57	0,43	kusta0014 tfdD;similar to chloromuconate cycloisomerase
scal00789	1558	51	0,43	ABC-type transport protein (ATPase component)
scal01698	1559	139	0,43	hypothetical protein
scal00827	1560	44	0,43	hypothetical protein GbemDRAFT_2772
scal02162	1561	94	0,43	putative PmbA/TldD modulator of DNA gyrase
scal02046	1562	65	0,43	undecaprenyldiphospho-muramoylpentapeptide beta-N-acetylglucosaminyl transferase
scal00513	1563	90	0,43	similar to Na(+)-translocating NADH-quinone reductasesubunit E

scal02400	1564	32	0,43	strongly similar to acetolactate synthase regulatory subunit
scal02599	1565	61	0,43	similar to tRNA isopentenylpyrophosphate transferase
scal00201	1566	52	0,43	A subunit of FO ATP synthase
scal02060	1567	36	0,43	siroheme synthetase CysG
scal04069	1568	50	0,41	Glycosyltransferase involved in cell wall biogenesis-like
scal02087	1569	48	0,41	similar to cyclohexadienyl dehydrogenase/5-enolpyruvylshikmate 3-P synthase
scal03214	1570	133	0,41	unknown protein
scal00562	1571	76	0,41	expressed putative glycine cleavage system P-protein
scal00310	1572	68	0,41	6-phosphofructokinase
scal03534	1573	37	0,41	fructose-6-phosphate aldolase
scal02435	1574	52	0,41	hypothetical protein
scal00413	1575	25	0,41	protein of unknown function DUF101
scal03076	1576	28	0,41	putative rubrerythrin
scal00433	1577	169	0,41	strongly similar to excinuclease ABC subunit A
scal00455	1578	33	0,41	putative glutathione peroxidase
scal04126	1579	25	0,41	conserved hypothetical protein
scal01856	1580	38	0,41	putative RNA-directed DNA polymerase
scal00731	1581	58	0,41	hypothetical protein
scal01244	1582	76	0,41	putative 4Fe-4S ferredoxin
scal03981	1583	97	0,41	similar to membrane protein component of ABC transporter for proline or glycine betaine
scal01170	1584	88	0,41	histidine ammonia-lyase
scal03026	1585	89	0,41	putative NADP-dependent glyceraldehyde-3-phosphate dehydrogenase, aldehyde dehydrogenase family
scal03342	1586	79	0,41	hypothetical protein PH1879
scal00627	1587	166	0,41	isoleucine tRNA synthetase
scal01840	1588	38	0,41	similar to phosphoglycolate phosphatase (EC 3.1.3.18)
scal01101	1589	24	0,41	putative transferase/hydrolase
scal00299	1590	43	0,41	Peptidase S1C, Do
scal00637	1591	25	0,41	putative response regulator protein
scal01609	1592	33	0,41	similar to ABC-transporter component
scal00733	1593	51	0,41	strongly similar to dihydropteroate synthase
scal01618	1594	30	0,41	Conserved hypothetical protein 52
orf05054	1595	14	0,41	two component, sigma54 specific, transcriptional regulator, Fis family
scal01838	1596	46	0,41	hypothetical protein Moth_1420
scal03975	1597	21	0,41	No hits found
scal03643	1598	60	0,41	sensory box protein/sigma-54 dependent DNA-binding response regulator
scal04234	1599	26	0,41	conserved hypothetical protein
scal03120	1600	21	0,41	response regulator protein
scal00549	1601	43	0,41	similar to dethiobiotin synthetase
scal03386	1602	120	0,41	No hits found
scal00073	1603	46	0,41	unknown protein
scal00211	1604	32	0,41	hypothetical protein
scal02102	1605	119	0,41	similar to multidrug resistance-like ATP-binding protein MdlB
orf05347	1606	100	0,41	thiamine pyrophosphate enzyme
scal01669	1607	94	0,41	type II and III secretion system protein
scal03179	1608	111	0,41	TPR Domain containing protein
scal02226	1609	60	0,41	hypothetical protein
scal04099	1610	62	0,41	putative cobalamin biosynthesis protein CbiD
scal03611	1611	24	0,41	hypothetical protein Ppro_3386
scal02469	1612	44	0,41	Phenazine biosynthesis PhzC/PhzF protein

scal00922	1613	72	0,41	similar to 3-dehydroquinate synthase
scal00213	1614	85	0,41	similar to heat shock protease DegP/HtrA
scal02908	1615	34	0,41	putative SAM radical protein
scal01922	1616	71	0,41	predicted pyridoxal phosphate-dependent enzyme
scal01563	1617	57	0,41	strongly similar to fructose-1,6-bisphosphatase
scal02648	1618	36	0,41	V-type ATPase subunit E
scal00540	1619	87	0,41	UDP-N-acetylmuramate-L-alanine ligase
scal00851	1620	35	0,41	hypothetical protein MM_1983
scal03036	1621	87	0,41	putative fixG like iron sulfur protein
scal01961	1622	89	0,39	similar to flagellar MS ring protein
scal01679	1623	22	0,39	No hits found
scal01524	1624	181	0,39	cation efflux protein CzcA
scal03419	1625	58	0,39	hypothetical protein MM_2979
scal00888	1626	19	0,39	reticulocyte binding protein
scal01512	1627	74	0,39	putative flavocytochrome C fumarate reductase
scal01670	1628	47	0,39	TPR Domain containing protein
scal00048	1629	123	0,39	putative molybdopterin oxidoreductase
scal04296	1630	22	0,39	conserved hypothetical protein
scal03038	1631	18	0,39	conserved hypothetical protein
scal03543	1632	54	0,39	strongly similar to chromosome partitioning protein
scal01308	1633	96	0,39	similar to arginyl-tRNA synthetase
scal00478	1634	125	0,39	D-alanine--D-alanine ligase domain protein
scal03205	1635	78	0,39	strongly similar to histidinol dehydrogenase
scal00975	1636	70	0,39	focA formate/nitrite transport protein
scal02354	1637	98	0,39	glucose-6-phosphate isomerase
scal03916	1638	49	0,39	hypothetical protein Gura_1746
scal03924	1639	29	0,39	elongation factor P protein
orf04963	1640	17	0,39	No hits found
scal02011	1641	43	0,39	orotidine 5'-phosphate decarboxylase
scal01298	1642	26	0,39	conserved hypothetical protein
scal03632	1643	38	0,39	phosphoenolpyruvate phosphomutase
scal00613	1644	53	0,39	conserved hypothetical protein
scal03515	1645	197	0,39	similar to DNA polymerase III, alpha subunit
scal02601	1646	45	0,39	hypothetical proetein
scal00171	1647	38	0,39	expressed conserved hypothetical protein
scal04021	1648	67	0,39	PEGA
scal01497	1649	20	0,39	No hits found
scal02008	1650	22	0,39	response regulator receiver
scal01273	1651	76	0,39	metal dependent phosphohydrolase
scal03167	1652	54	0,39	Succinylglutamate desuccinylase/aspartoacylase
scal00090	1653	30	0,39	hypothetical protein LNTAR_09149
scal02313	1654	16	0,39	hypothetical protein HCH_04178
scal04216	1655	112	0,39	DNA polymerase B region
scal00532	1656	58	0,39	hypothetical protein MB2181_02330
scal01957	1657	74	0,39	strongly similar to sigma 54 response regulator
scal03985	1658	97	0,39	oligopeptidase PepF
scal02424	1659	73	0,39	glutamyl-tRNA(Gln) amidotransferase subunit A
scal03152	1660	30	0,39	hypothetical protein
scal04110	1661	68	0,39	seryl-tRNA synthetase

scal01734	1662	89	0,39	putative MSHA biogenesis protein MshL
scal02933	1663	17	0,39	No hits found
scal02583	1664	76	0,39	conserved hypothetical protein
scal00791	1665	65	0,39	putative DNA-directed DNA polymerase
orf03644	1666	16	0,39	plasmid maintenance system killer
scal03660	1667	79	0,39	NAD/NADP transhydrogenase beta subunit
scal01890	1668	24	0,39	putative plasmid maintenance protein HigB with helix turn helix motiv
orf00618	1669	12	0,39	No hits found
scal03380	1670	175	0,39	COG2303: Choline dehydrogenase and related flavoproteins
scal02645	1671	75	0,39	V-type ATPase subunit B
orf02597	1672	11	0,39	cytosine deaminase
scal01035	1673	24	0,39	No hits found
scal01991	1674	90	0,39	hypothetical protein SYNW0989
scal00633	1675	38	0,39	thiamin phosphate synthase
scal04292	1676	62	0,39	putative N6-adenine-specific DNA methylase
scal01301	1677	74	0,39	catalase
orf00160	1678	15	0,39	No hits found
scal02123	1679	23	0,39	ferric uptake regulator protein
scal02853	1680	57	0,39	glutamate 5-kinase proB
scal00599	1681	52	0,39	expressed putative phosphoserine phosphatase
scal02273	1682	67	0,39	putative sodium proton, Na+/H+ antiporter NhaP
scal00879	1683	24	0,39	putative transcriptional regulator, CopG family
scal01083	1684	34	0,39	hypothetical protein
scal00402	1685	33	0,39	putative phosphoesterase
scal00399	1686	80	0,39	similar to sodium dependent potassium uptake system TrkH
scal00410	1687	34	0,39	conserved hypothetical protein
scal00047	1688	49	0,39	cysteine biosynthesis protein CysQ
scal02885	1689	37	0,39	stongly similair to DNA polymerase I
scal01236	1690	94	0,39	strongly similar to oxaloacetate (OadA) or methylmalonyl-CoA decarboxylase (MmdA)
scal03459	1691	57	0,37	NAD-dependent epimerase/dehydratase
scal04316	1692	13	0,37	ribosomal protein L28
scal04100	1693	33	0,37	similar to precorrin-6 methyltransferase CbiE
scal04030	1694	22	0,37	cyclic nucleotide-binding domain protein
scal00317	1695	57	0,37	similat to tetraacyldisaccharide 4'-kinase
scal01969	1696	36	0,37	flagellar motor protein motB
scal02416	1697	38	0,37	Biotin--acetyl-CoA-carboxylase ligase
scal00889	1698	68	0,37	putative lipopolysaccharide biosynthesis protein
orf02254	1699	9	0,37	unknown protein
scal04000	1700	66	0,37	oligopeptide ABC transport protein
scal04098	1701	35	0,37	conserved hypothetical protein
scal00892	1702	60	0,37	similar to succinyl-diaminopimelate desuccinylase
scal03530	1703	148	0,37	strongly similar to exinuclease ABC, protein A
orf05346	1704	73	0,37	NADP-dependent glyceraldehyde-3-phosphate dehydrogenase
scal00785	1705	21	0,37	putative iojap-like protein
scal01393	1706	49	0,37	tolB protein
scal00720	1707	74	0,37	conserved hypothetical protein
scal04047	1708	17	0,37	putative transposase
scal01478	1709	59	0,37	peptidase M20
scal00899	1710	52	0,37	putative signal peptide peptidase SppA

orf04284	1711	20	0,37	No hits found
scal01208	1712	138	0,37	4Fe-4S binding protein
scal04226	1713	73	0,37	hypothetical FAD oxidoreductase protein
scal00849	1714	63	0,37	hypothetical protein
scal02624	1715	78	0,37	unknown protein
scal01189	1716	32	0,37	unknown protein
scal03868	1717	59	0,37	similar to KDO kinase (2-keto-3-deoxy-D-manno-octulosonic acid)
scal03003	1718	53	0,37	hypothetical protein LNTAR_04981
scal02615	1719	60	0,37	similar to glucose-1-phosphate thymidylyltransferase
scal00458	1720	118	0,37	putative phosphoenolpyruvate synthase
scal01041	1721	166	0,37	Acriflavin resistance protein
scal00301	1722	31	0,37	unknown protein
scal01658	1723	21	0,37	response regulator receiver protein
scal03435	1724	118	0,37	TonB-dependent copper receptor
orf06899	1725	13	0,37	regulatory protein, FmdB family
scal01436	1726	22	0,37	DoxD-like family protein
scal00126	1727	20	0,37	hypothetical protein
scal04172	1728	27	0,37	expressed conserved hypothetical protein mostly found in microaerophilic metal-metabolizing and/or nitrogen-fixing microbes
scal02115	1729	49	0,37	hypothetical protein Sfum_1584
scal00119	1730	92	0,37	conserved hypothetical protein with diguanylate cyclase/phosphodiesterase sensory box/GGDEF domains
scal02581	1731	75	0,37	similar to p-aminobenzoate synthetase, component I
scal01477	1732	70	0,37	protein of unknown function DUF201
scal02312	1733	68	0,37	Chloride channel, core
orf04554	1734	16	0,37	transposase, IS4 family protein
scal01481	1735	101	0,37	COG0642: Signal transduction histidine kinase
scal02643	1736	38	0,37	V-type ATPase subunit I
scal00105	1737	181	0,37	hypothetical protein PM8797T_28259
scal01920	1738	48	0,37	GHMP kinase
scal00962	1739	52	0,37	hypothetical protein VvadDRAFT_0226
scal01984	1740	174	0,37	putative flagellar hook associated protein
scal02579	1741	88	0,37	strongly similar to GMP synthase (glutamine-hydrolyzing)
scal04321	1742	12	0,37	30S ribosomal protein S21
scal00854	1743	26	0,37	unknown protein
scal01178	1744	74	0,37	hypothetical protein
scal01567	1745	49	0,37	similar to histone deacetylase
scal00422	1746	149	0,37	putative TPR repeat protein
scal02429	1747	32	0,37	fumarate reductase, cytochrome b subunit, putative
scal01055	1748	58	0,37	putative norVW like flavoprotein
scal01403	1749	21	0,37	conserved hypothetical protein
scal02716	1750	16	0,37	putative Ferredoxin-thioredoxin reductase
scal01376	1751	23	0,37	unknown protein
scal00325	1752	110	0,37	similar to DNA helicase
scal01712	1753	109	0,37	predicted orf
scal02128	1754	40	0,37	putative histidinol phosphatase
scal01307	1755	61	0,37	hypothetical protein DSY4502
scal00522	1756	43	0,37	endonuclease IV
scal00151	1757	62	0,37	putative multiheme protein with xx heme / cxxch and 1 cxxxch motive part of putative nrf cluster
scal00232	1758	40	0,37	conserved hypothetical protein
scal03033	1759	129	0,37	similar to multimodular CopA (P-type ATPase)

scal03851	1760	30	0,37	zinc metalloprotease
scal02922	1761	65	0,37	putative outermembrane efflux protein
scal01407	1762	21	0,37	conserved hypothetical protein
scal00664	1763	42	0,37	putative Uracil-DNA glycosylase
scal00277	1764	53	0,37	unknown protein
scal00821	1765	53	0,37	putative cycloartenol synthase-like protein
scal01382	1766	28	0,34	No hits found
scal03784	1767	31	0,34	putative CDP-diacylglycerol--glycerol-3-phosphate 3-phosphatidyltransferase
scal03936	1768	81	0,34	putative GlcNAc transferase
scal01212	1769	22	0,34	conserved hypothetical protein
scal01671	1770	87	0,34	type II secretion system protein E
scal03616	1771	15	0,34	putative tetraheme cytochrome c protein ; 3 and 4th cxxch motive missing
scal00489	1772	17	0,34	strongly similar to 50S ribosomal protein L31
scal02241	1773	16	0,34	unknown protein
scal04130	1774	17	0,34	helix-turn-helix domain protein
scal03741	1775	74	0,34	glycogen synthase
scal00965	1776	11	0,34	prevent-host-death family protein
scal01672	1777	50	0,34	strongly similar to twiching motility protein PilT
scal03867	1778	31	0,34	conserved hypothetical protein
scal01949	1779	58	0,34	hypothetical protein
scal01088	1780	64	0,34	membrane spanning protein ribonuclease BN-like family
orf01202	1781	81	0,34	hypothetical protein
scal01480	1782	70	0,34	hypothetical FAD oxidoreductase protein
scal01994	1783	74	0,34	glycosyl transferase, family 2
scal00492	1784	37	0,34	beta lactamase like protein
scal02248	1785	44	0,34	beta-lactamase-like protein
scal00404	1786	52	0,34	putative iron sulfur protein
scal02307	1787	56	0,34	TrkA-N domain protein
scal01293	1788	72	0,34	putative tetraheme c554 cytochrome protein
scal04184	1789	18	0,34	hypothetical protein RPA2819
orf00195	1790	12	0,34	unknown protein
scal03834	1791	50	0,34	ornithine cyclodeaminase
scal03777	1792	23	0,34	No hits found
scal02266	1793	33	0,34	similar to endonuclease III
scal02573	1794	18	0,34	strongly similar to RNAse P protein
scal01493	1795	16	0,34	No hits found
scal00229	1796	30	0,34	putative solvent tolerance protein
scal00542	1797	50	0,34	PAS/PAC sensor signal transduction histidine kinase
scal03813	1798	34	0,34	TPR repeat
scal00366	1799	141	0,34	unknown protein
scal00669	1800	21	0,34	similar to small heat shock protein
scal00877	1801	68	0,34	putative serine/theonine protein kinase
scal01114	1802	26	0,34	No hits found
scal03470	1803	73	0,34	Phosphate-selective porin O and P
scal00284	1804	49	0,34	putative ABC membrane protein
scal00165	1805	50	0,34	SAM radical iron sulfur protein; putative heme d1 biosynthesis protein nirJ
scal01250	1806	20	0,34	response regulator protein
scal01354	1807	100	0,34	strongly similar to DNA ligase
scal02357	1808	21	0,34	onserved hypothetical protein

orf03979	1809	11	0,34	hypothetical protein N47_N26560
scal01062	1810	17	0,34	putative two component sensor kinase
scal01299	1811	57	0,34	putative aspartate aminotransferase
scal00283	1812	59	0,34	putative ABC membrane protein
scal01549	1813	30	0,34	conserved hypothetical protein; probable fumarate hydratase, beta subunit
scal02858	1814	33	0,34	apsK adenylylsulfate (adenosine 5'-phosphosulfate) kinase
scal00735	1815	40	0,34	hypothetical protein
scal04108	1816	34	0,34	conserved hypothetical lolA like membrane protein,
scal02848	1817	17	0,34	Protein of unknown function DUF159
scal02979	1818	66	0,34	GTPase-like protein
orf05850	1819	10	0,34	putative periplasmic protein
scal03178	1820	64	0,34	response regulatory protein (atoC)
orf03595	1821	9	0,34	unknown protein
scal01989	1822	94	0,34	TPR repeat
scal00349	1823	103	0,34	acetyl-coa synthetase ADP forming
scal00371	1824	23	0,34	similar to ATP-dependent protease Lon
scal02980	1825	47	0,34	No hits found
scal02171	1826	25	0,34	50S ribosomal protein L9
scal02927	1827	47	0,34	Strongly similar to O-sialoglycoprotein endopeptidase
scal00961	1828	99	0,34	putative ABC-type transport protein involved in gliding motility
scal04106	1829	28	0,34	putative SAM radical protein
scal02294	1830	62	0,34	ntrC like sigma 54 response regulator
scal03318	1831	24	0,32	putative cytochrome c protein with 1 cxxch motive
scal00405	1832	70	0,32	putative cytochrome p450 hydroxylase MmcK
scal03547	1833	23	0,32	hypothetical protein SAV5188
scal03836	1834	17	0,32	glycoside hydrolase, family 13-like
scal00307	1835	173	0,32	hypothetical protein
scal03537	1836	73	0,32	hypothetical protein
scal00146	1837	63	0,32	aminotransferase class III gabT
scal03778	1838	76	0,32	similar to DNA repair protein RecN
scal02991	1839	58	0,32	strongly similar to 8-amino-7-oxononanoate synthase
scal03651	1840	49	0,32	phenylalanyl-tRNA synthetase alpha chain
scal00251	1841	52	0,32	hypothetical protein ObacDRAFT_3297
scal02001	1842	43	0,32	glycosyl transferase, family 2
scal02954	1843	54	0,32	glucose-1-phosphate thymidylyltransferase
scal00380	1844	33	0,32	conserved hypothetical protein; possible dTDP-6-deoxy-L-hexose 3-O-methyltransferase
scal03025	1845	29	0,32	hypothetical planctomyces protein with 1 cxxch motive
scal00504	1846	16	0,32	putative plasmid maintenance protein HigB with helix turn helix motiv
scal00338	1847	40	0,32	resC type II cytochrome c biogenesis protein
scal01158	1848	79	0,32	putative YidC inner membrane protein
orf07466	1849	25	0,32	No hits found
scal02791	1850	19	0,32	unknown protein
scal01976	1851	47	0,32	putative flagellar biosynthetic protein FlhB
scal00726	1852	37	0,32	putative molybdopterin synthesis protein MoeB and thiamin biosynthesis protein ThiF
scal01741	1853	29	0,32	General secretion pathway protein G
scal00732	1854	39	0,32	conserved hypothetical protein
scal02590	1855	41	0,32	hypothetical protein
scal00038	1856	50	0,32	protein of unknown function DUF1568
scal01184	1857	65	0,32	general secretion pathway protein A

orf05376	1858	11	0,32	hypothetical protein Rcas_0956
scal04103	1859	32	0,32	putative methyltransferase
scal00179	1860	23	0,32	unknown protein
scal02393	1861	41	0,32	similar to phosphatidylglycerol-prolipoprotein diacylglyceryl transferase
scal02271	1862	74	0,32	conserved hypothetical protein
scal00240	1863	41	0,32	strongly similar to 3-deoxy-manno-octulosonate cytidylyltransferase
scal00714	1864	19	0,32	conserved hypothetical protein
scal02425	1865	57	0,32	putative tpr repeat protein
scal02666	1866	33	0,32	putative aquaporin
scal01284	1867	19	0,32	protein tyrosine phosphatase
scal01738	1868	56	0,32	putative general secretion pathway protein G
scal01126	1869	64	0,32	strongly similar to DNA repair protein RadA
scal01136	1870	52	0,32	hypothetical protein
scal02497	1871	24	0,32	unknown protein
scal03504	1872	36	0,32	8-oxoguanine DNA glycosylase
scal03613	1873	67	0,32	N-6 DNA methylase
scal00102	1874	59	0,32	putative nickel hydrogenase, large subunit
scal03678	1875	127	0,32	type I DNA restriction-modification system
scal00895	1876	55	0,32	competence/damage-inducible protein CinA
scal01053	1877	31	0,32	putative beta lactamase / hydroxyacylglutathione hydrolase
scal01359	1878	7	0,32	glutamyl-tRNA(Gln) amidotransferase subunit A
orf03678	1879	14	0,32	strongly similar to
scal01089	1880	19	0,32	unknown protein
scal02832	1881	25	0,32	similar to 2'-5' RNA ligase
scal03028	1882	22	0,32	secreted protein
scal04324	1883	14	0,32	conserved protein
scal02390	1884	111	0,32	conserved hypothetical protein
orf05093	1885	9	0,32	No hits found
scal02259	1886	32	0,32	hypothetical protein FP0987
scal01358	1887	51	0,32	adenylate/guanylate cyclase
scal00120	1888	68	0,32	thiamine biosynthesis protein ThiH
scal03417	1888	19	0,32	strongly similar to 2-amino-4-hydroxy-6-hydroxymethyldihydropteridine
scal01111	1890	54	0,32	similar to general secretory system type II protein, membrane component
scal03864	1891	25	0,32	hydrolase
scal03843	1892	28	0,32	conserved hypothetical protein
orf07565	1893	131	0,32	putative phosphoketolase
scal00173	1894	50	0,32	efflux transporter, RND family, MFP subunit
scal03232	1895	58	0,32	similar to 3-oxoacyl-[acyl-carrier-protein] synthase I
orf00853	1896	10	0,32	HicB family protein
scal01956	1897	61	0,32	putative sensor signal histdine kinase
scal03799	1898	107	0,32	similar to ferrous iron transport protein B (GTP binding)
scal03011	1899	29	0,32	putative transcriptional regulator, TetR family
scal01107	1900	98	0,32	metal dependent phosphohydrolase
scal02434	1901	45	0,32	strongly similar to D-alanine:D-alanine ligase
scal04072	1902	45	0,32	conserved hypothetical protein
scal02515	1903	76	0,32	Carbamoyltransferase
scal02668	1904	175	0,32	putative ATP-dependent nuclease subunit A
scal03721	1905	47	0,32	capsular polysaccharide biosynthesis protein
scal03430	1906	56	0,32	conserved hypothetical protein

scal00012	1907	30	0,32	putative resA type II cytochrome c biogenesis protein
scal02803	1908	62	0,32	similar to GTP-binding protein
scal01266	1909	61	0,32	No hits found
scal01216	1910	67	0,32	strongly similar to methionyl-tRNA synthetase
scal02864	1911	116	0,32	similar to ATP-dependent RNA helicase (RNA polymerase associated protein)
scal02937	1912	41	0,32	similar to thiamine monophosphate kinase
scal02345	1913	51	0,32	putative flavoprotein norVW
scal01674	1914	43	0,32	putative pmbA, tldD modulator protein of DNA gyrase
scal04101	1915	30	0,32	strongly similar to precorrin-2 C20-methyltransferase
scal01438	1916	18	0,32	protein of unknown function DUF1018
scal00604	1917	63	0,32	integral membrane sensor signal transduction histidine kinase
scal03538	1918	15	0,32	similar to site-specific tyrosine recombinase
scal02387	1919	48	0,32	filamentation induced by cAMP protein Fic
scal02204	1920	29	0,32	hypothetical protein Cag_0554
scal00423	1921	75	0,32	putative iron sulfur SAM radical protein
scal03226	1922	34	0,32	conserved hypothetical protein
scal03862	1923	63	0,32	similar to Mg-protoporphyrin monomethylester cyclase
scal02572	1924	46	0,30	strongly similar to glutamate formimidoyltransferase
scal00815	1925	74	0,30	putative lipoprotein
scal03747	1926	24	0,30	No hits found
scal01962	1927	44	0,30	similar to Flagellar Rotor Protein FliG
scal00980	1928	458	0,30	cyclic beta 1-2 glucan synthetase
scal00139	1929	51	0,30	Homoserine dehydrogenase
scal00817	1930	42	0,30	MoxR like ATPase
scal00142	1931	97	0,30	hypothetical protein
scal00341	1932	24	0,30	No hits found
scal04203	1933	71	0,30	putative histidine sensor kinase
scal01907	1934	36	0,30	flagellar hook-associated protein FlgL
scal02004	1935	47	0,30	Oxidoreductase, N-terminal:Oxidoreductase, C-terminal:Homoserine dehydrogenase, NAD-binding
scal00976	1936	122	0,30	unknown protein n terminus
scal01619	1937	61	0,30	similar to poly(A) polymerase
scal00933	1938	102	0,30	Spermine synthase
scal03494	1939	22	0,30	putativecytochrome c CII protein
scal01289	1940	36	0,30	hypothetical protein
orf04049	1941	12	0,30	hypothetical protein Palpr_1403
scal01002	1942	59	0,30	sec independent translocase tatC
scal03514	1943	54	0,30	strongly similar to sigma 54 response regulator
scal00145	1944	32	0,30	expressed putative TPR repeat protein
scal01185	1945	41	0,30	similar to O-linked GlcNAc transferase
scal03634	1946	68	0,30	hypothetical protein aq_922
scal04029	1947	35	0,30	HPr kinase
scal01270	1948	118	0,30	Integrins alpha chain
scal01661	1949	25	0,30	general secretion pathway protein H
scal01583	1950	59	0,30	citrate synthase
scal04033	1951	30	0,30	putative ABC type transport protein, permease component
scal00386	1952	56	0,30	hypothetical protein alr5239
scal01226	1953	34	0,30	hypothetical protein
scal02224	1954	46	0,30	conserved hypothetical protein
orf02096	1955	9	0,30	carbon storage regulator

scal01345	1956	189	0,30	putative histidine kinase
scal00403	1957	35	0,30	hypothetical COG1355 protein
orf04639	1958	18	0,30	GreA/GreB family elongation factor
scal03376	1959	74	0,30	Carbon starvation regulatory protein
scal00941	1960	29	0,30	conserved hypothetical protein
orf06414	1961	8	0,30	KpsF/GutQ family protein
scal01946	1962	27	0,30	hypothetical protein
scal01843	1963	54	0,30	hypothetical protein PM8797T_19465
scal02491	1964	17	0,30	putative thioesterase
scal02044	1965	46	0,30	phospho-N-acetylmuramoyl-pentapeptide transferase
scal01943	1966	20	0,30	conserved hypothetical protein
scal01908	1967	17	0,30	hypothetical protein
scal03734	1968	210	0,30	PEGA
scal00429	1969	65	0,30	similar to apolipoprotein N-acyltransferase
scal01124	1970	36	0,30	unknown protein
scal00450	1971	48	0,30	ABC transport protein
scal00598	1972	39	0,30	putative phosphoserine phosphatase
scal00376	1973	41	0,30	polysaccharide deacetylase
scal02903	1974	30	0,30	putative zip zinc/iron permease
scal02900	1975	25	0,30	RuvC crossover junction endodeoxyribonuclease
scal00959	1976	50	0,30	ABC transporter related
scal02293	1977	95	0,30	putative histidine kinase
scal00035	1978	23	0,30	hypothetical protein
scal00722	1979	28	0,30	putative thymidylate synthase ThyX
scal02067	1980	47	0,30	conserved hypothetical protein
scal00228	1981	104	0,30	transport protein, putative
scal00656	1982	46	0,30	putative phosphatidate cytidylyltransferase; phophoserine phosphatase
scal02918	1983	105	0,30	Secretion protein HlyD like protein
scal00896	1984	40	0,30	response regulator receiver modulated diguanylate cyclase
scal02373	1985	26	0,30	similar to protein relase factor 2 methylase (HemK)
scal02759	1986	34	0,30	putative enzyme (3.4.-)
scal02976	1987	49	0,30	M16 like peptidase
scal03221	1988	30	0,30	hypothetical protein
scal01758	1989	20	0,30	No hits found
scal01154	1990	83	0,30	unknown protein
scal04187	1991	106	0,30	putative tpr repeat protein
scal01552	1992	37	0,30	putative molybdopterin containing oxidoreductase N terminus
scal03190	1993	26	0,30	unknown protein
scal00049	1994	34	0,30	hypothetical protein
scal00311	1995	67	0,30	probable spermidine synthase
scal00901	1996	27	0,30	hypothetical protein
scal00704	1997	24	0,30	putative COG1238 membrane protein
scal00164	1998	58	0,30	Fe-S oxidoreductase
scal01582	1999	18	0,30	No hits found
scal03466	2000	66	0,30	conserved hypothetical protein
scal03661	2001	45	0,30	NAD/NADP transhydrogenase alpha subunit
scal03869	2002	46	0,30	similar to heptosyl transferase I
scal02895	2003	24	0,30	ham1 like purine NTP pyrophosphatase
scal02377	2004	30	0,30	hypothetical DUF558 protein

orf04371	2005	11	0,30	hypothetical protein CferDRAFT_0756
scal02302	2006	33	0,30	putative serine threonine protein kinase
scal01848	2007	33	0,30	cysteine biosynthesis protein CysQ
scal00793	2008	26	0,30	transcriptional repressor protein
scal01186	2009	29	0,30	hypothetical protein SUN_2188
scal02003	2010	51	0,30	Predicted Fe-S oxidoreductase
scal03439	2011	71	0,30	similar to pyridine nucleotide-disulphide oxidoreductase
scal02192	2012	91	0,30	putative adenylate cyclase protein
scal00476	2013	27	0,30	N-formylglutamate amidohydrolase
scal03803	2014	32	0,30	hypothetical protein DP0730
scal01917	2015	15	0,30	hypothetical protein Adeh_3052
scal03299	2016	49	0,30	conserved hypothetical protein with CBS and DUF21 domains
scal00425	2017	74	0,27	penicillin-binding protein 2;cell division protein ftsI
scal02124	2018	20	0,27	putative resA type II cytochrome c biogenesis protein
scal02066	2019	36	0,27	hypothetical protein
scal01666	2020	23	0,27	Pilus assembly protein, PilO
scal02419	2021	19	0,27	putative cytochrome c CII protein
scal00493	2022	44	0,27	conserved hypothetical duf185 protein
orf06279	2023	20	0,27	Glyoxalase/bleomycin resistance protein/dioxygenase
scal01562	2024	24	0,27	pantothenate kinase-like protein
scal00715	2025	65	0,27	hypothetical protein
orf06064	2026	11	0,27	No hits found
scal03909	2027	139	0,27	putative type I restriction protein
scal03283	2028	22	0,27	No hits found
scal02740	2029	61	0,27	putative histidine sensor kinase
scal01404	2030	51	0,27	kinesin light chain-like protein
scal03562	2031	18	0,27	putative TonB-dependent receptor protein
scal03861	2032	21	0,27	hypothetical protein NB231_15063
scal04305	2033	9	0,27	undefined product
scal04027	2034	49	0,27	probable hydrolase
scal00424	2035	46	0,27	putative cell shape-determining protein RodA
scal03652	2036	87	0,27	phenylalanyl-tRNA synthetase beta chain
scal01091	2037	15	0,27	hypothetical protein
scal01818	2038	29	0,27	strong similarity to L-threonine dehydrogenase
scal00057	2039	22	0,27	hypothetical protein
scal04240	2040	97	0,27	transketolase
scal00095	2041	15	0,27	No hits found
orf04090	2042	8	0,27	hypothetical protein N47_E43310
orf04962	2043	8	0,27	conserved hypothetical protein
scal04196	2044	56	0,27	putative RNA modification enzyme miaB
scal01522	2045	125	0,27	cation efflux protein CzcA
scal01575	2046	31	0,27	No hits found
scal02691	2047	38	0,27	conserved hypothetical protein
scal01294	2048	47	0,27	hypothetical protein
scal00262	2049	18	0,27	No hits found
scal01630	2050	37	0,27	Formyl transferase-like
orf00768	2051	11	0,27	conserved domain protein
scal03733	2052	35	0,27	conserved hypothetical protein
scal02466	2053	67	0,27	sucrose phosphorylase

scal02905	2054	31	0,27	dihydroorotate oxidase electron transfer subunit
scal00928	2055	15	0,27	No hits found
scal04090	2056	39	0,27	conserved hypothetical protein
scal00350	2057	52	0,27	acetate kinase
scal00766	2058	26	0,27	similar to multidrug efflux transporter AcrB
scal03925	2059	47	0,27	coproporphyrinogen III oxidase
scal02367	2060	24	0,27	putative nitroreductase
scal02030	2061	21	0,27	conserved hypothetical protein containing DUF204
scal00597	2062	30	0,27	serine/threonine protein kinase
orf05043	2063	10	0,27	hypothetical protein HRM2_11790
scal03642	2064	29	0,27	sigma-54 dependent DNA-binding response regulator
scal01837	2065	40	0,27	conserved hypothetical signal transduction HD GYP protein
scal00037	2066	12	0,27	hypothetical protein BGP_2009
scal03604	2067	51	0,27	similar to UDP-N-acetylmuramate:L-alanine ligase MurC
scal00130	2068	13	0,27	putative sensor protein
scal03209	2069	23	0,27	hypothetical protein DP1940
scal00728	2070	21	0,27	putative GTP cyclohydrolase I
scal03405	2071	15	0,27	expressed hypothetical protein
scal03010	2072	51	0,27	adenylate/guanylate cyclase
scal02999	2073	11	0,27	hypothetical YcfA family protein
scal02085	2074	22	0,27	No hits found
scal04070	2075	45	0,27	similar to lipopolysaccharide core biosynthesis protein
scal01206	2076	28	0,27	carbonic anhydrase
scal01519	2077	61	0,27	cation efflux protein CzcB
scal01936	2078	43	0,27	unknown protein
scal03316	2079	52	0,27	FAD-dependent pyridine nucleotide-disulphide oxidoreductase
scal03580	2080	27	0,27	hypothetical protein
scal03841	2081	24	0,27	strongly similar to DNA helicase RuvA
scal04144	2082	39	0,27	similar to pyruvate ferredoxin oxidoreductase alpha subunit
scal02841	2083	15	0,27	hypothetical protein PM8797T_11776
scal02685	2084	32	0,27	conserved hypothetical protein
scal00531	2085	22	0,27	conserved hypothetical cog2928 protein
scal02670	2086	120	0,27	putative ATP-dependent nuclease subunit B
scal01945	2087	41	0,27	conserved hypothetical protein
scal01916	2088	29	0,27	putative glycosyltransferase
scal00760	2089	28	0,27	carboxylate-amine ligase
scal00870	2090	63	0,27	putative sensor signal histdine kinase
scal00622	2091	36	0,27	binding-protein-dependent transport systems inner membrane component
scal01990	2092	82	0,27	TPR repeat
scal04330	2093	11	0,27	conserved protein
scal01381	2094	35	0,27	Aspartate--ammonia ligase
scal00466	2095	14	0,27	No hits found
scal03779	2095	28	0,27	hypothetical protein pc0098
scal00830	2097	37	0,27	Possible nucleotide sugar epimerase
scal00451	2098	50	0,27	ABC transport protein
scal01935	2099	71	0,27	similar to two component sensor histidine kinase
scal02593	2100	34	0,27	similar to TDP-rhamnose synthetase, NAD(P)-binding
scal01093	2101	124	0,27	AcrB/AcrD/AcrF family protein
scal02412	2102	12	0,27	recombinase

scal03067	2103	42	0,27	putative efflux protein
scal00858	2104	20	0,27	Strongly similar to molybdenum cofactor biosynthesis protein C
scal01932	2105	57	0,27	putative SAM radical iron sulfur protein
scal02182	2106	17	0,27	putative thioredoxin/thiol-disulfide isomerase
scal00565	2107	16	0,27	hypothetical protein PputGB1DRAFT_1121
scal02200	2108	27	0,27	conserved hypothetical cog4399 protein
scal04028	2109	43	0,25	hypothetical protein
scal03041	2110	25	0,25	PKD
scal01964	2111	49	0,25	strongly similar to flagellar ATPase fliI
scal01629	2112	39	0,25	hypothetical protein Gmet_0883
scal01902	2113	28	0,25	flagellar L-ring protein
scal00623	2114	53	0,25	ABC-type dipeptide/oligopeptide/nickel transport system, permease components
scal00536	2115	39	0,25	strongly similar to S-adenosylmethionine-tRNA ribosyltransferase-isomerase
scal03349	2116	23	0,25	hypothetical planctomyces protein with 1 cxxch motive
scal03218	2117	12	0,25	intervening sequence, 23S rRNA
scal00150	2118	51	0,25	putative multiheme protein of putative nrf cluster
scal02597	2119	16	0,25	GreA/GreB family elongation factor
scal00275	2120	12	0,25	unknown protein
scal00437	2121	38	0,25	hypothetical anammox protein
scal02079	2122	18	0,25	strongly similar to aspartate transcarbamoylase regulatory chain
scal03633	2123	25	0,25	CDP-alcohol phosphatidyltransferase
scal00628	2124	10	0,25	hypothetical COG1872 protein
scal01595	2125	38	0,25	geranylgeranyl hydrogenase BchP, putative
scal02264	2126	29	0,25	hypothetical protein
scal01667	2127	24	0,25	No hits found
scal00387	2128	42	0,25	hypothetical protein P700755_08164
scal04045	2129	19	0,25	unknown protein
scal03352	2130	75	0,25	hypothetical protein
scal00172	2131	117	0,25	similar to transcription-repair coupling factor
scal00576	2132	72	0,25	putative type I restriction-modification system, M subunit; N-6 Adenine-specific DNA methylase
scal01951	2133	17	0,25	ATP-binding region, ATPase-like:Histidine kinase A-like
scal00235	2134	40	0,25	Alanine racemase
scal02303	2135	22	0,25	hypothetical cog1943 protein
scal02227	2136	17	0,25	conserved hypothetical protein
orf07431	2137	7	0,25	No hits found
scal03512	2138	39	0,25	similar to cofactor modifying protein
scal03331	2139	32	0,25	Methyltransferase type 11
scal02000	2140	40	0,25	predicted pyridoxal phosphate-dependent enzyme apparently involved in regulation of cell wall biogenesis
scal04204	2141	34	0,25	hypothetical DUF534 protein
scal00724	2142	76	0,25	conserved hypothetical protein containing Cbb3-type cytochrome oxidase subunit
scal04239	2143	62	0,25	alpha-amylase family protein
scal02582	2144	15	0,25	FxsA protein
scal02274	2145	28	0,25	maf protein
scal03683	2146	23	0,25	putative outermembrane protein
scal01974	2147	30	0,25	polar flagellar assembly protein FliP
scal04058	2148	42	0,25	hypothetical protein
scal00100	2149	14	0,25	putative response regulator
scal03477	2150	54	0,25	citrate synthase I
scal04249	2151	24	0,25	putative sensor protein

scal01744	2152	16	0,25	similar to purine binding chemotaxis protein
scal01657	2153	23	0,25	ATP-binding region, ATPase-like
scal01087	2154	19	0,25	glucose-methanol-choline oxidoreductase
scal02523	2155	49	0,25	No hits found
scal00564	2156	30	0,25	lipoic acid synthetase
scal01651	2157	53	0,25	hypothetical protein DET1510
scal00436	2158	27	0,25	putative cytochrome biogenesis protein ccsA
scal02884	2159	36	0,25	unknown protein
scal01531	2160	62	0,25	signal transduction histidine kinase
scal01050	2161	13	0,25	response regulator receiver protein
scal02295	2162	88	0,25	similar to sensor histidine kinase/response regulator protein NtrY
scal04220	2163	56	0,25	hypothetical protein
scal02663	2164	40	0,25	carbamoyl-phosphate synthase small subunit
scal01732	2165	34	0,25	TPR repeat
scal01109	2166	62	0,25	strongly similar to general secretory system type II protein, ATPase component
scal03383	2167	30	0,25	No hits found
orf02805	2168	8	0,25	No hits found
scal02830	2169	26	0,25	conserved hypothetical protein
scal03298	2170	35	0,25	conserved hypothetical protein with CBS and DUF21 domains
scal03189	2171	38	0,25	nucleotide-diphosphate-sugar epimerase
scal02540	2172	47	0,25	DNA-damage-inducible protein F
scal01110	2173	65	0,25	strongly similar to general secretory system type II protein, ATPase component
scal04127	2174	23	0,25	similar to nicotinic acid mononucleotide adenylyltransferase, NAD(P) requiring
scal00713	2175	31	0,25	putative ion transport protein
scal02953	2176	26	0,25	hypothetical protein
scal03229	2177	52	0,25	hypothetical protein
scal01981	2178	44	0,25	putative COG2206 signal transduction protein
scal01954	2179	63	0,25	hypothetical protein
scal01905	2180	16	0,25	flagellar biosynthesis protein FlgN
scal01923	2181	35	0,25	NAD-dependent epimerase/dehydratase
scal00118	2182	37	0,25	hypothetical protein, putative glucokinase
scal03587	2183	11	0,25	similar to type I restriction-modification system, R subunit
scal02947	2184	22	0,25	putative glycosyltransferase
scal01998	2185	77	0,25	TPR repeat
scal00966	2186	14	0,25	PilT protein, N-terminal
scal00593	2187	28	0,25	expressed putative serine/threonine protein kinase
scal01176	2188	23	0,25	unknown protein
orf04828	2189	8	0,25	hypothetical protein TTC1365
scal04093	2190	38	0,25	cobalamin biosynthesis protein cbiB
scal00675	2191	53	0,25	hypothetical protein
scal01739	2192	39	0,25	putative type II secretion system protein
scal00348	2193	12	0,25	conserved hypothetical protein
scal03034	2194	73	0,25	strongly similar to cbb3-type cytochrome c oxidase subunit 1 CcoN
orf05349	2195	17	0,25	No hits found
scal02934	2196	25	0,25	similar to thymidylate kinase
scal02257	2197	10	0,25	putative pemK / YdcE protein
scal00331	2198	69	0,25	oligopeptide transporter, OPT family
scal00514	2199	62	0,25	similar to Na(+)-translocating NADH-quinone reductase subunit F
scal01095	2200	52	0,25	cation efflux system protein

scal00279	2201	41	0,25	ABC transport protein
scal00920	2202	25	0,25	hypothetical protein MJ0912
scal02544	2203	24	0,25	tRNA (guanine-N(7))-methyltransferase
scal00842	2204	29	0,25	putative TonB like protein
orf06996	2205	12	0,25	PilT domain-containing protein
orf06794	2206	8	0,25	unknown protein
scal02742	2207	36	0,25	putative sensor histidine kinase
scal00591	2208	68	0,25	amtB ammonium transport protein
scal03078	2209	47	0,25	K+ transporter Trk
scal00292	2210	17	0,25	putative nitric-oxide reductase subunit B
scal01868	2211	24	0,25	conserved hypothetical protein
scal03785	2212	72	0,25	strongly similar to DNA translocase cell division ATPase ftsK
scal01146	2213	48	0,23	tRNA modification GTPase TrmE
scal00621	2214	52	0,23	oligopeptide ABC transporter, periplasmic oligopeptide-binding protein
scal02923	2215	56	0,23	putative response regulator
scal03151	2216	31	0,23	hypothetical protein
scal00379	2217	32	0,23	similar to cofactor modifying protein
scal03796	2218	18	0,23	conserved hypothetical protein
scal00008	2219	51	0,23	Glycosyltransferase plus another conserved domain
scal00529	2220	49	0,23	hypothetical protein
scal00166	2221	33	0,23	putative YdjC family protein
scal02644	2222	21	0,23	V-type ATPase subunit D
scal03877	2223	32	0,23	Conserved hypothetical protein 374
scal01265	2224	38	0,23	glycosyl transferase, group 1
orf01337	2225	9	0,23	hypothetical protein Gura_1141
scal02592	2226	22	0,23	putative lipoprotein
scal03599	2227	28	0,23	similar to diaminopimelate epimerase
scal00050	2228	31	0,23	putative histidine kinase
scal01325	2229	10	0,23	conserved hypothetical protein
scal00027	2230	20	0,23	conserved hypothetical protein
scal00971	2231	14	0,23	Hemerythrin HHE cation binding domain protein
scal02914	2232	42	0,23	K+-dependent Na+/Ca+ exchanger related-protein
scal01963	2233	21	0,23	hypothetical protein
scal03173	2234	30	0,23	universal stress protein uspA
scal01523	2235	53	0,23	cation efflux protein
scal02913	2236	54	0,23	putative cytochrome c protein with 1 cxxch motive
scal00970	2237	25	0,23	Similar to ribosomal-protein-serine acetyltransferase
scal02002	2238	14	0,23	putatiave oxidoreductase, Gfo/Idh/MocA family/transferase
scal01931	2239	39	0,23	putative heme d1 biosynthesis protein NirJ
scal02353	2240	57	0,23	604 aa unknown protein
scal02338	2241	16	0,23	putative molybdopterin-guanine dinucleotide biosynthesis protein B
scal00245	2242	51	0,23	ribulose-bisphosphate carboxylase-like protein; rubisco-like protein
scal03536	2243	62	0,23	similar to ABC transporter MsbA
scal03533	2244	16	0,23	protein containing DUF1456
scal02600	2245	54	0,23	hypothetical protein
scal00826	2246	34	0,23	O-methyltransferase, family 2:Generic methyltransferase
scal02363	2247	13	0,23	protein of unknown function DUF86
scal01254	2248	22	0,23	methyltransferase domain protein
scal03797	2249	31	0,23	hypothetical protein

scal04091	2250	57	0,23	Cobyric acid synthase CobQ
orf03932	2251	11	0,23	conserved hypothetical protein
scal02706	2252	20	0,23	hypothetical protein ObacDRAFT_3297
scal02920	2253	34	0,23	sensor histidine kinase
scal01906	2254	70	0,23	flagellar hook-associated protein FlgK
scal01845	2255	26	0,23	putative tpr repeat pilF like protein
scal03382	2256	44	0,23	No hits found
orf04544	2257	6	0,23	conserved hypothetical protein
scal01123	2258	34	0,23	unknown protein
scal02532	2259	13	0,23	unknown protein
scal02738	2260	13	0,23	hypothetical protein L8106_24275
scal01218	2261	21	0,23	conserved hypothetical protein
scal00385	2262	26	0,23	No hits found
scal03304	2263	33	0,23	DNA polymerase III beta subunit
scal02608	2264	22	0,23	hypothetical protein Msp_0602
scal01526	2265	42	0,23	tolc like outer membrane efflux protein
scal03578	2266	23	0,23	similar to AcrA family multidrug efflux protein
scal00818	2267	37	0,23	protein potential transcriptional repressor Not4hp
scal00276	2268	12	0,23	unknown protein
scal01036	2269	36	0,23	Response regulator receiver:Metal-dependent phosphohydrolase, HD subdomain
scal02350	2270	39	0,23	conserved hypothetical protein
scal01256	2271	22	0,23	No hits found
scal03070	2272	14	0,23	universal stress protein uspA
scal02602	2273	31	0,23	unknown protein
scal00167	2274	32	0,23	hypothetical DUF799 lipoprotein
scal03577	2275	22	0,23	putative TonB-dependent receptor protein
orf07078	2276	8	0,23	conserved hypothetical protein
scal00973	2277	56	0,23	hypothetical protein
scal01904	2278	9	0,23	putative flagellar biosynthesis protein
scal03433	2279	11	0,23	Biopolymer transport protein ExbD/TolR
scal00883	2280	28	0,23	putative metallophosphoesterase
scal02943	2281	15	0,23	hypothetical protein PH0057
scal02228	2282	20	0,23	probable transferase Cj1035c
scal01631	2283	31	0,23	undecaprenyl phosphate 4-deoxy-4-formamido-L-arabinose transferase
scal00601	2284	15	0,23	putative cytochrome c protein with 1 cxxch motive
scal03336	2285	33	0,23	Transketolase-like
orf03629	2286	12	0,23	hypothetical protein ROSEINA2194_01298
scal02498	2287	21	0,23	hypothetical protein Dde_2147
scal02586	2288	27	0,23	putative protein-L-isoaspartate O-methyltransferase
scal00795	2289	29	0,23	hypothetical COG3375 protein
scal00300	2290	28	0,23	No hits found
scal01080	2291	22	0,23	hypothetical protein
scal03416	2292	36	0,21	probable transcriptional regulator
scal01910	2293	17	0,21	putative tpr repeat protein
scal00260	2294	27	0,21	Xylose isomerase domain protein TIM barrel
scal03303	2295	38	0,21	similar to chromosomal replication initiator protein DnaA
scal00749	2296	12	0,21	No hits found
scal03236	2297	10	0,21	muconolactone delta-isomerase
scal01928	2298	42	0,21	anaerobic magnesium-protoporphyrin IX monomethyl ester cyclase, Elongator protein 3/MiaB/NifB family

scal03535	2299	30	0,21	similar to dTDP-6-deoxy-L-lyxo-4-hexulose reductase (Rmld)
scal01911	2300	50	0,21	oxidoreductase, Gfo/Idh/MocA family/transferase hexapeptide repeat protein
scal03999	2301	29	0,21	oligopeptide ABC transport protein
scal04081	2302	12	0,21	No hits found
scal01230	2303	11	0,21	No hits found
scal03758	2304	11	0,21	partial beta subunit of CO dehydrogenase / acetyl-CoA synthase (AcsA)
scal03927	2305	22	0,21	dolichyl-phosphate mannosyltransferase
scal01277	2306	75	0,21	hypothetical transcriptional regulator
scal01137	2307	40	0,21	ref|ZP_00056585.2| COG1032: Fe-S oxidoreductase
scal01636	2308	50	0,21	undecaprenyl phosphate-alpha-4-amino-4-deoxy-L-arabinose arabinosyl transferase
orf01936	2309	6	0,21	conserved hypothetical protein
scal03495	2310	11	0,21	putative methyltransferase
scal01740	2311	51	0,21	type II secretion system protein E
scal01514	2312	18	0,21	hypothetical protein Noc_2984
scal02368	2313	44	0,21	similar to 3-dehydroquinate dehydratase / shikimate 5-dehydrogenase
scal02890	2314	26	0,21	similar to p-hydroxybenzoate: octaprenyltransferase
scal03004	2315	29	0,21	hypothetical protein RB9424
scal02437	2316	12	0,21	flagellar hook assembly protein
scal01589	2317	22	0,21	hypothetical protein Mbur_2165
scal03500	2318	9	0,21	hypothetical protein
scal03697	2319	55	0,21	putative sensory box protein/sigma-54 dependent DNA-binding response regulator
scal02005	2320	45	0,21	glycosyl transferase, family 2
orf03947	2321	7	0,21	KilA-N, DNA-binding domain protein
scal03489	2322	8	0,21	hypothetical protein GZ23H7_26
orf07594	2323	8	0,21	No hits found
scal01983	2324	12	0,21	flagellar protein FliS
scal00042	2325	14	0,21	putative response regulator protein
scal02647	2326	16	0,21	conserved hypothetical protein
scal02202	2327	30	0,21	conserved hypothetical protein
scal01269	2328	41	0,21	hypothetical protein PTH_2774
scal03080	2329	25	0,21	putative chromosome partitioning protein
scal01261	2330	36	0,21	glycosyl transferase, group 1
scal00822	2331	32	0,21	putative cycloartenol synthase-like protein
scal00353	2332	37	0,21	diphosphate--fructose-6-phosphate 1-phosphotransferase
scal00028	2333	16	0,21	hypothetical protein
scal01977	2334	60	0,21	flagellar biosynthesis protein FlhA
scal00911	2335	12	0,21	universal stress protein uspA
scal01972	2336	11	0,21	flagellar motor switch protein FliN
scal01548	2337	27	0,21	No hits found
scal04314	2338	11	0,21	ribosome-binding factor A
scal03531	2339	9	0,21	hypothetical protein BamMC406DRAFT_3746
scal04054	2340	41	0,21	putative transport protein
scal03677	2341	28	0,21	type I DNA restriction-modification system
scal01628	2342	24	0,21	Polysaccharide deacetylase
scal01483	2343	20	0,21	Response regulator receiver:Transcriptional regulatory protein-like
scal00875	2344	18	0,21	hypothetical protein
orf01249	2345	4	0,21	ref|ZP_06347520.2| rubredoxin
scal00383	2346	22	0,21	hypothetical protein SAV5733
scal02372	2347	19	0,21	Nucleotidyl transferase

orf01272	2348	20	0,21	carboxylate-amine ligase
scal03345	2349	31	0,21	Radical SAM domain protein
scal00972	2350	11	0,21	unknown protein with 1 cxxch motive
orf06935	2351	13	0,21	No hits found
scal01085	2352	86	0,21	similar to nitrogen assimilation regulatory protein
scal01046	2353	53	0,21	multi-sensor hybrid histidine kinase
orf04191	2354	6	0,21	hypothetical protein DaAHT2_0088
scal01140	2355	38	0,21	Undecaprenyl-phosphate galactose phosphotransferase
scal00816	2356	25	0,21	puttaive cog1721 protein
scal03880	2357	27	0,21	Maf-like protein
scal03434	2358	13	0,21	tolQ protein
scal02642	2359	26	0,21	V-type ATPase subunit I
scal01496	2360	11	0,21	conserved hypothetical protein
scal00871	2361	30	0,21	putative gamma subunit of DNA polymerase III
scal01940	2362	21	0,21	similar to chemotaxis protein methyltransferase
scal01081	2363	18	0,21	HAD-superfamily hydrolase subfamily IA, variant 3
scal03914	2364	28	0,21	putative C4-dicarboxylate transporter/malic acid transport protein
scal00089	2365	17	0,21	DNA polymerase III, epsilon subunit and related 3'-5' exonucleases
orf05413	2366	6	0,18	CopG family protein
scal00294	2367	16	0,18	unknown protein
scal03513	2368	59	0,18	similar to histidine kinase protein
scal00332	2369	19	0,18	123aa long hypothetical protein
scal01986	2370	51	0,18	putative glycosyl transferase
scal03805	2371	14	0,18	putative TonB-dependent receptor protein
scal00520	2372	88	0,18	hypothetical duf748 protein
scal00303	2373	10	0,18	unknown anammox protein
scal00302	2374	12	0,18	protein of unknown function DUF583
scal02609	2375	13	0,18	similar to sensor histidine kinase/response regulator protein NtrY
scal04016	2376	14	0,18	hypothetical protein MTH112
scal03381	2377	21	0,18	No hits found
orf06208	2378	8	0,18	hypothetical protein
scal03488	2379	11	0,18	No hits found
scal01736	2380	17	0,18	No hits found
scal00964	2381	25	0,18	No hits found
scal00752	2382	13	0,18	hypothetical protein PM8797T_05430
scal02113	2383	34	0,18	conserved hypothetical protein; putative fortimicin production protein
scal01238	2384	9	0,18	hypothetical protein; putative gamma subunit (oadG) of oxaloacetate decarboxylase
scal04136	2385	16	0,18	Glycosyl transferase, family 39
scal00161	2386	25	0,18	similar to glycosyltransferase family 2
scal04074	2387	18	0,18	strongly similar to two-component response regulator OmpR/PhoB family
scal01864	2388	52	0,18	helicase
scal02298	2389	9	0,18	hypothetical protein
scal01924	2390	37	0,18	Magnesium-protoporphyrin IX monomethyl ester (oxidative) cyclase
scal01253	2391	25	0,18	Glycosyl transferase, family 2
scal02580	2392	22	0,18	similar to branched-chain amino-acid aminotransferase
scal03436	2393	12	0,18	unknown protein
orf01341	2394	7	0,18	lysine 2,3-aminomutase YodO family protein
scal02508	2395	30	0,18	glycosyl transferase, group 1
scal01056	2396	12	0,18	putative rubrerythrin

scal03464	2397	17	0,18	similar to phoU
scal00043	2398	9	0,18	unkown protein
scal01191	2399	16	0,18	hypothetical protein
scal03681	2400	44	0,18	similar to multidrug efflux transporter AcrB
scal04327	2401	8	0,18	undefined product
scal02068	2402	20	0,18	strongly similar to tRNA pseudouridine synthase I
scal00828	2403	39	0,18	Coenzyme B12-binding:Radical SAM
orf07145	2404	8	0,18	No hits found
scal01357	2405	7	0,18	105aa long hypothetical protein
scal01882	2406	12	0,18	conserved hypothetical protein
scal03465	2407	18	0,18	Phosphate import ATP-binding protein pstB
scal00220	2408	21	0,18	hypothetical protein
scal03215	2409	8	0,18	unknown protein
scal00411	2410	28	0,18	pleiotropic regulatory protein DegT
scal00443	2411	74	0,18	hypothetical protein
scal01815	2412	28	0,18	hypothetical protein WD0259
scal03335	2413	24	0,18	Pyruvate dehydrogenase (lipoamide)
scal03158	2414	9	0,18	No hits found
scal00323	2415	46	0,18	Radical SAM iron sulfur protein
scal04065	2416	19	0,18	similar to glucosyl-transferase
scal01942	2417	15	0,18	hypothetical protein
scal01735	2418	13	0,18	No hits found
scal04202	2419	20	0,18	hypothetical two-component response regulator protein
scal01869	2420	26	0,18	putative SAM radical protein
scal00372	2421	12	0,18	No hits found
scal01953	2422	24	0,18	unknown protein
scal01980	2423	42	0,18	putative response regulator protein
scal03612	2424	10	0,18	Hypothetical Protein
scal01571	2425	16	0,18	No hits found
scal02027	2426	10	0,18	protein of unknown function DUF369
scal02510	2427	29	0,18	Lipopolysaccharide heptosyltransferase II
scal02352	2428	11	0,18	putative DNA uptake competence protein comEA
orf00356	2429	4	0,18	transposase IS200-family protein
scal04084	2430	7	0,18	putative plasmid maintenance protein HigB with helix turn helix motiv
scal02121	2431	17	0,18	probable transcriptional regulator protein
scal02916	2432	12	0,18	159aa long hypothetical protein
scal04325	2433	5	0,18	conserved protein
scal04179	2434	26	0,18	similar to ATPase involved in chromosome partitioning
scal02894	2435	7	0,18	putative abundant 10 kDa cytochrome c-552
scal02203	2436	25	0,18	conserved hypothetical protein
scal01817	2437	20	0,18	strong similarity to L-threonine dehydrogenase
scal02119	2438	12	0,18	shikimate kinase
scal00566	2439	18	0,18	lipoate protein ligase
scal01521	2440	11	0,18	unknown protein
scal04086	2441	24	0,18	hypothetical protein pc1890
scal00298	2442	61	0,18	hypothetical protein BSG1_03760
orf04021	2443	7	0,18	hypothetical protein N47_G36670
scal04313	2444	8	0,18	partial transcription elongation protein NusA
scal01461	2445	28	0,18	putative cytochrome c3 hydrogenase subunit

scal02695	2446	49	0,18	TPR Domain containing protein
scal04148	2447	19	0,18	conserved hypothetical protein
scal01199	2448	16	0,18	hypothetical protein with DTXW domain
orf03354	2449	6	0,18	transcriptional regulator, AbrB family
scal02238	2450	17	0,18	putative phosphopantothenoylcysteine synthetase
scal03895	2451	6	0,18	unknown protein
scal03463	2452	12	0,18	conserved hypothetical protein
scal03668	2453	21	0,18	conserved hypothetical protein
scal02262	2454	30	0,18	similar to lipoprotein releasing system transmembrane protein LolC
scal02542	2455	54	0,16	similar to multidrug resistance-like ATP-binding protein MdlB
scal03387	2456	8	0,16	3-isopropylmalate dehydratase small subunit
scal02850	2457	8	0,16	hypothetical protein
scal03772	2457	22	0,16	strongly similar to phosphoribosylaminoimidazole-succinocarboxamide (SAICAR) synthase
scal01237	2459	29	0,16	strongly similar to methylmalonyl-CoA or oxaloacetate decarboxylase (beta subunit)
scal03223	2460	18	0,16	unknown protein
orf02527	2461	5	0,16	unknown protein
scal03496	2462	15	0,16	hypothetical protein
scal04019	2463	42	0,16	Sodium:dicarboxylate symporter
scal00487	2464	21	0,16	similar to protein methyltransferase
scal01214	2465	30	0,16	similar to sigma-54 dependent transcriptional response regulator
scal04004	2466	5	0,16	putative transposase IS4 family
scal04199	2467	8	0,16	No hits found
orf03793	2468	35	0,16	similar to magnesium transporter MgtE
scal01038	2469	28	0,16	response regulator receiver protein
scal02915	2470	11	0,16	159aa long hypothetical protein
scal00619	2471	7	0,16	Radical SAM domain protein
orf06671	2472	5	0,16	No hits found
scal00464	2473	12	0,16	RNA-directed DNA polymerase (reverse transcriptase
orf03594	2474	10	0,16	flagellar protein FliS
scal01988	2475	43	0,16	putative tpr repeat protein
scal01132	2476	14	0,16	No hits found
scal03491	2477	7	0,16	No hits found
scal03548	2478	10	0,16	No hits found
scal02924	2479	11	0,16	unknown protein
scal01394	2480	8	0,16	type I DNA restriction-modification system
scal00691	2481	46	0,16	putative diheme protein
scal01517	2482	68	0,16	cation efflux protein of CzcA family
scal00261	2483	60	0,16	conserved putative chloride channel
scal00580	2484	24	0,16	hydrogenase expression/formation protein
scal03589	2485	7	0,16	No hits found
scal00182	2486	9	0,16	putative cobalamin (vitamin B12) biosynthesis CbiX
scal01257	2487	28	0,16	glycosyltransferase
orf04707	2488	5	0,16	No hits found
scal00502	2489	10	0,16	hypothetical protein
scal04017	2490	11	0,16	hypothetical protein
scal01268	2491	7	0,16	No hits found
scal00257	2492	26	0,16	hypothetical protein all0419
scal02475	2493	41	0,16	putative type I restriction modification enzyme
scal00632	2494	9	0,16	bifunctional phosphoribosyl-AMP cyclohydrolase/ phosphoribosyl-ATP pyrophosphatase hisI

scal03782	2495	7	0,16	No hits found
scal00015	2496	20	0,16	hypothetical protein ALPR1_15964
scal01520	2497	27	0,16	cation efflux protein CzcC
scal00923	2498	29	0,16	similar to multifunctional folylpolyglutamate synthase/dihydrofolate synthase
scal04105	2499	32	0,16	expressed conserved hypothetical COG5316 protein
scal03344	2500	36	0,16	DNA polymerase IV (family X)
scal01934	2501	29	0,16	putative glycosyl transferase
orf05573	2502	5	0,16	conserved hypothetical protein
scal01925	2503	24	0,16	similar to molybdopterin cofactor synthesis protein MoaA
orf01112	2504	4	0,16	ref|YP_427519.2| bifunctional sulfur carrier protein/thiazole synthase protein
scal00384	2505	32	0,16	anaerobic magnesium-protoporphyrin IX monomethyl ester cyclase, Elongator protein 3/MiaB/NifB family
scal03943	2506	8	0,16	PilT protein domain protein
scal02261	2507	6	0,16	hypothetical protein CE1742
orf07469	2508	4	0,16	No hits found
scal00149	2509	25	0,16	putative multiheme protein of putative nrf cluster
scal01592	2510	8	0,16	No hits found
scal03238	2511	16	0,16	putative tpr repeat protein
scal01967	2512	18	0,16	flagellar motor protein motA
scal02272	2513	11	0,16	hypothetical protein
scal03735	2514	8	0,16	unknown protein
scal02514	2515	16	0,16	Predicted xylanase/chitin deacetilase
scal00465	2516	21	0,16	RNA-directed DNA polymerase (reverse transcriptase
scal01231	2517	26	0,16	strongly similar to glucose-1-phosphate adenylyltransferase
scal01559	2518	14	0,16	strongly similar to N-(5'-phosphoribosyl)anthranilate isomerase
scal01305	2519	11	0,16	No hits found
scal03831	2520	7	0,16	putative Excinuclease ABC, C subunit domain protein
scal02946	2521	20	0,16	Glycosyl transferase, family 2
scal01568	2522	13	0,16	similar to ribonuclease H
scal02206	2523	20	0,16	unknown protein*
scal00935	2524	7	0,16	RNA polymerase, sigma-24 subunit, ECF subfamily
orf05832	2525	3	0,14	No hits found
scal03473	2526	37	0,14	putative citrate transporter
scal01978	2527	35	0,14	putative flagellar biosynthetic protein FlhF
scal01933	2528	39	0,14	putative SAM radical iron sulfur protein
scal02892	2529	31	0,14	hypothetical protein
scal01841	2530	15	0,14	hypothetical protein MED92_16775
scal03588	2531	20	0,14	UvrD/REP helicase
scal04113	2532	8	0,14	sp|O58584|Y854_PYRHO UPF0076 protein PH0854
scal00486	2533	16	0,14	hypothetical protein
scal03774	2534	10	0,14	phosphoribosylcarboxyaminoimidazole carboxylase
scal00104	2535	17	0,14	putative hydrogenase gamma subunit
scal03833	2536	19	0,14	putative SAM radical protein
scal00477	2537	38	0,14	gamma-glutamyltransferase
scal03722	2538	7	0,14	No hits found
scal02704	2539	6	0,14	putative DNA topoisomerase VI subunit A
scal01373	2540	25	0,14	similar to two component sensor histidine kinase
scal01331	2541	9	0,14	hypothetical protein DoleDRAFT_3087
scal00180	2542	14	0,14	unknown protein
scal04020	2543	38	0,14	similar to uvrABC system protein C (UvrC exinuclease)

scal00729	2544	21	0,14	dihydropicolinate synthase
scal01213	2545	14	0,14	putative GAF sensor signal transduction histidine kinase
scal01462	2546	31	0,14	hypothetical anammox protein c terminus
scal01982	2547	9	0,14	hypothetical protein
scal00075	2548	6	0,14	unknown protein
scal00219	2549	14	0,14	conserved hypothetical COG3222 protein
scal01937	2550	13	0,14	Response regulator (CheY,wHTH domains)
scal04161	2551	9	0,14	hypothetical protein GSU2315
scal02901	2552	23	0,14	putative permease
orf01461	2553	6	0,14	conserved hypothetical protein
scal01777	2554	15	0,14	AP endonuclease, family 2
scal01395	2555	41	0,14	similar to Uncharacterized conserved protein
scal00612	2556	6	0,14	conserved hypothetical protein
scal03071	2557	7	0,14	hypothetical protein
scal03992	2558	5	0,14	Maturase; integron/retron-type RNA-directed DNA polymerase (Reverse transcriptase); part of type II intron
scal03441	2559	8	0,14	unknown anammox protein
scal01915	2560	21	0,14	Radical SAM domain protein
scal01192	2561	20	0,14	conserved hypothetical protein of aldo-/ketoreductase family
scal01441	2562	12	0,14	sulfate transport protein
scal00824	2563	23	0,14	Inositol-3-phosphate synthase
scal01930	2564	11	0,14	putative membrane protein
scal02496	2565	8	0,14	No hits found
orf00855	2566	3	0,14	No hits found
scal02090	2567	9	0,14	strongly similar to to proton-translocating NADH dehydrogenase I, 24 kDa subunit (NuoE)
scal01979	2568	15	0,14	RNA polymerase sigma factor for flagellar gene cluster
scal02112	2569	29	0,14	similar to magnesium transporter MgtE
scal02253	2570	6	0,14	No hits found
scal01743	2571	44	0,14	methyl-accepting chemotaxis sensory transducer
scal01187	2572	14	0,14	putative sulfate transport protein CysZ
scal00061	2573	14	0,14	glucose-6-phosphate 1-dehydrogenase
scal01258	2574	43	0,14	similar to membrane protein
scal01971	2575	19	0,14	putative flagellar motor switch protein FliM
scal01875	2576	22	0,14	No hits found
scal03228	2577	8	0,14	similar to holo-[acyl-carrier protein] synthase
scal03564	2578	5	0,14	glutamyl-tRNA(Gln) amidotransferase subunit A
scal00653	2579	9	0,14	hypothetical protein
scal02366	2580	34	0,14	Glycosyl transferase, family 39
scal03906	2581	8	0,14	putative DNA methylase
scal02263	2582	6	0,14	No hits found
scal04186	2583	16	0,14	hypothetical duf81 protein
orf02238	2584	4	0,14	prevent-host-death family protein
orf06195	2585	6	0,14	protein containing DUF1016
scal03254	2586	45	0,14	putatiave oxidoreductase, Gfo/Idh/MocA family/transferase
scal00746	2587	8	0,14	putative type I restriction modification enzyme
scal04158	2588	20	0,14	hypothetical protein
scal02465	2589	16	0,14	DNA ligase
scal03428	2590	24	0,14	transcriptional regulator, putative
scal01112	2591	13	0,14	No hits found
scal03920	2592	44	0,14	hypothetical membrane protein

scal02710	2593	6	0,14	No hits found
orf00350	2594	7	0,14	hypothetical protein
scal01896	2595	20	0,14	helicase
scal00278	2596	12	0,14	putative ABC transporter protein
scal01255	2597	16	0,14	hypothetical protein CGSSp18BS74_08005
scal03429	2598	9	0,14	unknown protein with 1 cxxch motive
scal01037	2599	22	0,14	Predicted signal transduction protein
scal03200	2600	6	0,14	No hits found
scal02813	2601	7	0,14	No hits found
scal03508	2602	6	0,14	DEAD/DEAH box helicase-like protein
scal03872	2603	25	0,14	hypothetical protein Ecol5_01001706
scal00981	2604	8	0,11	No hits found
scal01965	2605	8	0,11	unknown protein
scal03460	2606	18	0,11	hypothetical protein ObacDRAFT_3297
scal01092	2607	11	0,11	2-polyprenyl-3-methyl-5-hydroxy-6-metoxy-1,4-benzoquinol methylase
scal01125	2608	10	0,11	hypothetical protein WwSim0417
scal01901	2609	16	0,11	putative flagellar basal body P-ring biosynthesis protein FlgA
scal03113	2610	9	0,11	unknown protein
scal00202	2611	14	0,11	unknown protein
scal00155	2612	16	0,11	hypothetical protein MCA1931
scal04165	2613	14	0,11	hypothetical protein
orf04827	2614	5	0,11	No hits found
scal00485	2615	17	0,11	hypothetical protein L8106_24275
scal02829	2616	7	0,11	hypothetical protein BURPS1710b_3413
scal00085	2617	7	0,11	unknown protein
scal01249	2618	22	0,11	glucose-1-phosphate adenylyltransferase
scal01861	2619	20	0,11	similar to lipid-A-disaccharide synthase
orf01075	2620	4	0,11	hypothetical protein
scal02881	2621	22	0,11	Ste24 endopeptidase
scal01491	2622	2	0,11	No hits found
scal01528	2623	13	0,11	putative capsular polysaccharide biosynthesis protein
scal00077	2624	8	0,11	TRm17a putative transposase
scal03149	2625	8	0,11	hypothetical protein RB11399
scal00330	2626	12	0,11	No hits found
scal02300	2627	8	0,11	No hits found
orf06741	2628	17	0,11	similar to TonB-dependent receptor protein
scal04219	2629	8	0,11	hypothetical membrane protein
scal00230	2630	10	0,11	No hits found
scal01292	2631	8	0,11	No hits found
scal03121	2632	21	0,11	Smc22-1 (Probable osmotically inducible sensory protein)
scal01819	2633	7	0,11	peptide chain release factor
scal04215	2634	11	0,11	GpmB
scal00734	2635	12	0,11	hypothetical protein
scal01078	2636	19	0,11	similar to DNA polymerase III, delta subunit
scal02709	2637	7	0,11	hypothetical protein PphaDRAFT_0884
scal03384	2638	3	0,11	No hits found
scal03062	2639	33	0,11	Endonuclease
scal03798	2640	11	0,11	conserved hypothetical protein
scal01074	2641	8	0,11	No hits found

scal04048	2642	19	0,11	conserved hypothetical protein
scal03641	2643	40	0,11	two component sensor histidine kinase
scal00592	2644	7	0,11	unknown protein
orf06199	2645	8	0,11	No hits found
scal01865	2646	5	0,11	hypothetical protein ALPR1_11475
scal00006	2647	25	0,11	conserved hypothetical protein
scal01999	2648	12	0,11	Methyltransferase type 12
scal00750	2649	8	0,11	No hits found
scal03557	2650	4	0,11	No hits found
scal01918	2651	33	0,11	ref|XP_001029949.2| SLEI family protein
scal01877	2652	10	0,11	hemolytic protein HlpA-like
scal01929	2653	18	0,11	radical SAM domain protein, putative
scal03549	2654	10	0,11	hypothetical protein PphaDRAFT_0774
scal01662	2655	8	0,11	general secretion pathway protein G
scal00482	2656	5	0,11	No hits found
scal03124	2657	35	0,11	hypothetical membrane protein
scal01527	2658	6	0,11	unknown protein
orf06349	2659	4	0,11	prevent-host-death family protein
scal02356	2660	4	0,11	unknown protein
scal00561	2661	8	0,11	glycine cleavage system protein H
scal02650	2662	5	0,11	putative copper-transporting P-type ATPase
scal01108	2663	45	0,11	BNR repeat domain protein
scal00186	2664	11	0,11	hypothetical protein
scal01776	2665	7	0,11	GCN5-related N-acetyltransferase
scal00112	2666	12	0,11	hypothetical protein FB2170_01831
scal02535	2667	13	0,11	type IV pilus biogenesis protein PilC
scal03363	2668	5	0,11	No hits found
scal03539	2669	15	0,11	membrane protein, putative
scal00456	2670	11	0,11	cell division inhibitor SulA
scal01927	2671	16	0,11	Radical SAM domain protein
scal01921	2672	10	0,11	phosphoheptose isomerase
scal01118	2673	36	0,11	phospholipase D/competence protein ComEA helix-hairpin-helix domain protein
scal01914	2674	22	0,11	Methyltransferase type 11
scal04129	2675	5	0,11	hypothetical protein MED121_09870
scal02640	2676	7	0,11	unknown protein
scal01968	2677	14	0,11	flagellar motor protein motB
scal01437	2678	5	0,11	No hits found
scal00942	2679	19	0,11	hypothetical protein
scal03997	2680	7	0,11	PilT like protein
scal00131	2681	12	0,11	putative two-component histidine kinase
scal02436	2682	24	0,11	similar to flagellar hook-length control protein
scal03900	2683	12	0,11	strongly similar to N-acetylmuramoyl-L-alanine amidase (T7 lysozyme)
scal01966	2684	14	0,11	hypothetical protein
scal03077	2685	9	0,11	similar to hypoxanthine-guanine-xanthine phosphoribosyltransferase
scal03838	2686	5	0,11	unknown protein
orf06317	2687	4	0,11	No hits found
scal04137	2688	7	0,11	putative TonB-dependent receptor protein
scal01684	2689	10	0,11	unknown protein
scal01171	2690	13	0,11	similar to precorrin-6x reductase

scal01634	2691	27	0,11	hypothetical protein
scal00982	2692	4	0,11	phospholipid/glycerol acyltransferase
scal00296	2693	5	0,09	unknown protein
scal02506	2694	18	0,09	hypothetical protein
scal00256	2695	19	0,09	hypothetical protein DSM3645_20462
scal03014	2696	10	0,09	FMN binding Pyridoxamine 5'-phosphate oxidase
scal00736	2697	7	0,09	hypothetical upf0054 protein
scal00412	2698	19	0,09	Glycosyl transferase, family 4
scal01262	2699	15	0,09	possible methyltransferase
scal04205	2700	5	0,09	putative tonB receptor protein
scal00572	2701	8	0,09	No hits found
scal00569	2702	4	0,09	putative ATPase subunit of phosphoribosylaminoimidazole carboxylase
scal00242	2703	4	0,09	139aa long hypothetical protein
scal04121	2704	13	0,09	similar to UDP-glucose 4-epimerase
orf03576	2705	4	0,09	similar to flagellar biosynthetic protein FliQ
scal00696	2706	8	0,09	hypothetical vanZ protein
scal00226	2707	18	0,09	hypothetical protein
scal03391	2708	4	0,09	hypothetical protein CY0110_08411
scal01899	2709	10	0,09	flagellar hook protein FlgF
scal03468	2710	15	0,09	strongly similar to phosphate binding protein of ABC-type phosphate transport system
scal01774	2711	6	0,09	COG2089: Sialic acid synthase
scal04073	2712	16	0,09	similar to ADP-heptose:LPS heptosyltransferase II
scal02513	2713	17	0,09	DegT/DnrJ/EryC1/StrS aminotransferase
scal00176	2714	3	0,09	hypothetical protein CaO19_1550
orf05643	2715	2	0,09	conserved hypothetical protein
scal00723	2716	7	0,09	putative cob(I)alamin adenosyltransferase
scal00960	2717	10	0,09	ABC transporter, permease protein
scal04272	2718	8	0,09	hypothetical planctomyces protein with 1 cxxch motive
scal00929	2719	11	0,09	permease of the major facilitator superfamily
scal00426	2720	7	0,09	similar to rod shape-determining protein
scal00168	2721	15	0,09	hypothetical protein GeobDRAFT_0074
scal04212	2722	15	0,09	hypothetical protein ObacDRAFT_0943
scal00188	2723	8	0,09	conserved hypothetical protein
scal01584	2724	9	0,09	protein containing StAR-related lipid-transfer (START) domain
scal03163	2725	22	0,09	No hits found
scal01975	2726	11	0,09	flagellar biosynthetic protein FliR
scal01611	2727	13	0,09	hypothetical protein ALPR1_04288
scal02019	2728	4	0,09	putative plasmid maintenance protein HigB with helix turn helix motiv
scal02520	2729	6	0,09	putative rubrerythrin/rubredoxin protein
scal01396	2730	17	0,09	putative DNA repair photolyase,
scal00624	2731	27	0,09	ABC transporter, nucleotide binding/ATPase protein
scal00948	2732	4	0,09	No hits found
scal00556	2733	4	0,09	hypothetical protein Gura_1425
scal02042	2734	14	0,09	unknown anammox protein
scal00243	2735	4	0,09	conserved hypothetical cytosolic protein
scal04241	2736	9	0,09	putative ribose-5-phosphate isomerase B
orf05893	2737	3	0,09	hypothetical protein Cyan8802_4363
orf02833	2738	3	0,09	4Fe-4S ferredoxin iron-sulfur binding domain protein
scal01612	2739	5	0,09	159aa long hypothetical protein

scal03199	2740	3	0,09	unknown protein
orf00265	2741	12	0,09	hypothetical protein MCA1931
scal01247	2742	10	0,09	conserved hypothetical cog2928 protein
scal03240	2743	3	0,09	Conserved Hypothetical Protein
scal03280	2744	13	0,09	unknown protein
scal01484	2745	21	0,09	PAS
scal01451	2746	10	0,09	putative duf99 ABC transport protein
scal00355	2747	6	0,09	No hits found
scal01495	2748	9	0,09	unknown protein
scal03040	2749	8	0,09	conserved hypothetical protein
scal01958	2750	4	0,09	similar to flagellar basal-body rod protein FlgB
orf06275	2751	4	0,09	hypothetical protein Saut_2091
scal03686	2752	9	0,09	No hits found
scal00368	2753	39	0,09	similar to competence protein ComA
orf07625	2754	9	0,09	ceramide glucosyltransferase
scal02977	2755	4	0,09	No hits found
scal01086	2756	12	0,09	conserved hypothetical signal transduction HD GYP protein
scal01973	2757	6	0,09	flagellar protein FliO/FliZ
scal00977	2758	28	0,09	gb|AAF15293.3| erythrocyte membrane-associated giant protein antigen 332
scal02502	2759	19	0,09	similar to adenylylsulfate reductase chain A
scal03490	2760	5	0,09	hypothetical protein Rcas_0629
scal03037	2761	9	0,09	conserved hypothetical protein
scal02857	2762	6	0,09	putative sensory box protein/sigma-54 dependent DNA-binding response regulator
scal04221	2763	3	0,09	No hits found
scal02459	2764	5	0,09	conserved hypothetical protein
scal02423	2765	3	0,09	glutamyl-tRNA(Gln) amidotransferase subunit C
scal02549	2766	2	0,09	unknown protein
scal00055	2767	10	0,09	No hits found
scal03705	2768	6	0,09	conserved hypothetical protein
scal02651	2769	6	0,09	putative tpr repeat protein
scal01987	2770	21	0,09	putative TPR repeat protein
scal00985	2771	8	0,09	hypothetical protein Sfri_2035
scal02560	2772	3	0,09	No hits found
scal01826	2773	11	0,09	No hits found
scal02365	2774	6	0,07	hypothetical protein GSU0625
scal02511	2775	14	0,07	lipopolysaccharide heptosyltransferase II
scal03046	2776	7	0,07	ribulose-bisphosphate carboxylase-like protein; rubisco-like protein
scal03032	2777	8	0,07	putative cytochrome c protein with 1 cxxch motive
scal00692	2778	27	0,07	putative tetraheme protein
scal01941	2779	4	0,07	similar to chemotaxis protein CheY
scal00099	2780	16	0,07	putative sensor kinase
scal03684	2781	6	0,07	AcrA family efflux protein
scal01183	2782	8	0,07	No hits found
scal03170	2783	15	0,07	No hits found
scal04167	2784	12	0,07	hypothetical protein
scal01465	2785	11	0,07	conserved hypothetical cog1611 protein
scal03663	2786	4	0,07	No hits found
scal01862	2787	13	0,07	Transposase, IS4
orf05001	2788	3	0,07	hypothetical protein N47_H24130

scal02652	2789	4	0,07	No hits found
scal00884	2790	6	0,07	hypothetical protein
scal02341	2791	6	0,07	No hits found
scal04243	2792	15	0,07	putative glucokinase
scal03907	2793	18	0,07	putative restriction endonuclease
scal01259	2794	7	0,07	conserved hypothtical protein
scal01742	2795	13	0,07	hypothetical protein
scal02512	2796	7	0,07	Methyltransferase type 11
scal01733	2797	14	0,07	ATPase
scal00018	2798	14	0,07	hypothetical protein
scal02361	2799	19	0,07	Hypothetical protein
scal00183	2800	5	0,07	putative lipoprotein
scal02605	2801	3	0,07	No hits found
scal01532	2802	3	0,07	No hits found
scal03431	2802	5	0,07	No hits found
orf04886	2804	2	0,07	No hits found
scal02998	2805	17	0,07	similar to ubiquinone biosynthesis protein UbiB
scal01588	2806	13	0,07	putative carbohydrate-selective porin protein, OprB family
scal00719	2807	14	0,07	putative L-threonine-O-3-phosphate decarboxylase
scal01939	2808	16	0,07	diguanylate cyclase
scal03579	2809	18	0,07	similar to multidrug efflux transporter AcrB
scal04156	2810	10	0,07	similar to competence protein F
scal03682	2811	9	0,07	glycosyl transferase, family 39
scal00058	2812	5	0,07	strongly similar to SAM-dependent methyltransferase YecO
scal03146	2813	6	0,07	hypothetical protein
scal03739	2814	4	0,07	conserved hypothetical protein
scal03945	2815	3	0,07	transcriptional regulator, AbrB family
orf06194	2816	2	0,07	hypothetical protein N47_E50670
scal00703	2817	19	0,07	conserved hypothetical COG1355 protein
scal01970	2818	6	0,07	putative flagellar basal body-associated protein FliL
scal01049	2819	4	0,07	putative SAM radical protein
scal03971	2820	4	0,07	No hits found
scal03680	2821	8	0,07	Hypothetical Protein
scal03801	2822	20	0,07	Carbohydrate-selective porin
scal04120	2823	4	0,07	strongly similar to nucleoside diphosphate kinase
scal01827	2824	21	0,07	conserved hypothetical CheR like methyltransferase protein
scal04075	2825	16	0,07	hypothetical histidine kinase protein
orf04992	2826	3	0,07	hypothetical protein N47_E50670
scal02812	2827	6	0,07	rhodanese-like domain protein
scal02154	2828	6	0,07	putative outermembrane protein
scal01341	2829	8	0,07	hypothetical kinase protein
orf07275	2830	3	0,07	No hits found
scal01737	2831	13	0,07	No hits found
scal00790	2832	5	0,07	putative sugar epimerase
scal04188	2833	5	0,07	unknown protein
scal04301	2834	7	0,07	ceramide glucosyltransferase
scal00568	2835	5	0,07	putative glyoxalase protein
scal03966	2836	3	0,07	No hits found
scal01812	2837	4	0,07	No hits found

scal01912	2838	3	0,07	hypothetical protein TM1773
scal00337	2839	8	0,07	No hits found
scal02596	2840	6	0,07	hypothetical protein Faci_03000644
scal01435	2841	5	0,07	hypothetical protein Ping_2112
scal00093	2842	18	0,07	hypothetical protein Sbal_3630
scal02409	2843	8	0,07	putative transmembrane protein
scal04185	2844	7	0,07	hypothetical protein Fjoh_3013
scal00076	2845	6	0,07	putative transposon-related protein
scal02630	2846	5	0,07	conserved hypothetical nucleotide binding protein
scal04068	2847	14	0,07	Dolichyl-phosphate-mannose-protein mannosyltransferase
scal00162	2848	17	0,07	conserved hypothetical lipopolysaccharide biosynthesis protein
scal02359	2849	6	0,07	putative membrane protein
scal02543	2850	7	0,07	No hits found
scal04225	2851	3	0,07	cAMP-binding protein
scal00505	2852	3	0,07	hypothetical protein
scal02684	2853	2	0,07	Prevent-host-death protein
scal00823	2854	6	0,05	No hits found
scal00924	2855	3	0,05	ACT domain protein
scal00820	2856	16	0,05	conserved hypothetical protein
scal03857	2857	3	0,05	similar to ribonuclease R
orf05652	2858	2	0,05	excinuclease ABC C subunit domain-containing protein
scal01749	2859	4	0,05	No hits found
scal00206	2860	11	0,05	conserved hypothetical protein
scal01264	2861	16	0,05	Asparagine synthase, glutamine-hydrolyzing
scal02193	2862	32	0,05	TPR repeat protein
scal02570	2863	4	0,05	strongly similar to methylated-DNA-protein-cysteine S-methyltransferase
scal02997	2864	7	0,05	No hits found
scal03644	2865	9	0,05	unknown protein
scal02638	2866	3	0,05	No hits found
scal01139	2867	20	0,05	FAD-dependent pyridine nucleotide-disulphide oxidoreductase 4Fe-4S iron sulfur ferredoxin
scal03047	2868	9	0,05	No hits found
scal02617	2869	4	0,05	putative transcriptional regulator, AsnC family protein
scal01233	2870	14	0,05	NADH subunit 5
scal02317	2871	8	0,05	cellulose biosynthesis protein
scal01204	2872	14	0,05	NADH dehydrogenase (quinone)
orf05500	2873	5	0,05	No hits found
scal04299	2874	4	0,05	putative type I restriction modification enzyme
scal03636	2875	5	0,05	endonuclease V
scal00589	2876	3	0,05	unknown protein
orf01280	2877	2	0,05	protein of unknown function UPF0150
orf04638	2878	6	0,05	putative methylated-DNA--protein-cysteine methyltransferase
scal00495	2879	4	0,05	rhomboid like proten
orf01489	2880	2	0,05	similar to biotin-[acetyl-CoA-carboxylase] ligase
scal01061	2881	13	0,05	similar to two-component sensor histidine kinase
scal01829	2882	3	0,05	beta-alanine synthetase
scal03614	2883	5	0,05	Transposase, IS4
scal01926	2884	6	0,05	similar to molybdenum cofactor biosynthesis protein A
scal00697	2885	7	0,05	putative ubiE methyltransferase
scal01490	2886	1	0,05	No hits found

orf06188	2886	1	0,05	No hits found
scal02983	2888	3	0,05	No hits found
scal02620	2889	5	0,05	conserved hypothetical chemotaxis protein
scal03343	2890	4	0,05	Hypothetical protein RBTH_01524
scal01452	2891	5	0,05	putative ABC transport protein
scal01952	2892	8	0,05	hypothetical histidine kinase protein
scal00833	2893	11	0,05	putative Fe-S iron sulfur SAM radical protein
scal03544	2894	5	0,05	hypothetical protein
scal01919	2895	5	0,05	D-glycero-D-manno-heptose 1-phosphate guanosyltransferase
scal04097	2896	3	0,05	No hits found
scal03227	2897	9	0,05	similar to Na+-driven multidrug efflux protein NorM
scal00671	2898	8	0,05	No hits found
scal03274	2899	7	0,05	nucleotidyltransferase
scal03635	2900	5	0,05	aldo/keto reductase
scal01850	2901	2	0,05	conserved hypothetical protein
scal02047	2902	6	0,05	NHL repeat containing protein
scal01116	2903	4	0,05	No hits found
scal01492	2904	4	0,05	No hits found
scal04233	2905	8	0,05	conserved hypothetical protein
scal01846	2906	12	0,05	Peptidase M48, Ste24p
scal04275	2907	6	0,05	putative transposase
scal02880	2908	3	0,05	transposase, IS4
orf04788	2909	1	0,05	conserved hypothetical protein
scal02538	2910	5	0,05	Type I restriction enzyme EcoprrI specificity protein
scal02595	2911	10	0,05	hypothetical protein
scal03253	2912	9	0,05	Glutamine-scyllo-inositol transaminase
scal03389	2913	4	0,05	Carbonate dehydratase
scal01747	2914	4	0,05	No hits found
scal01664	2915	6	0,05	No hits found
scal01375	2916	9	0,05	conserved hypothetical CheR like methyltransferase protein
scal00234	2917	2	0,05	No hits found
scal00101	2918	3	0,05	[Ni, Fe] hydrogenase maturation protein
scal04178	2918	3	0,05	unknown protein
scal01834	2920	6	0,05	No hits found
scal02965	2921	6	0,05	site-specific recombinase, phage integrase family
scal01625	2922	4	0,05	No hits found
scal00768	2923	8	0,05	No hits found
scal03565	2924	3	0,05	protein containing DUF1016
scal02637	2925	3	0,02	No hits found
scal01445	2926	5	0,02	putative parB like chromosome partitioning protein
scal00304	2927	2	0,02	magnesium transporter
scal04087	2928	2	0,02	hypothetical protein PTD2_12459
orf00359	2929	1	0,02	hypothetical protein CferDRAFT_0718
orf05650	2929	1	0,02	hypothetical protein
orf06075	2931	3	0,02	TonB family protein
scal02481	2932	4	0,02	No hits found
scal03309	2933	3	0,02	conserved hypothetical protein
scal01851	2934	15	0,02	helicase, putative
scal03937	2935	2	0,02	hypothetical protein GP2143_02439

orf01047	2936	3	0,02	unnamed protein product
orf05838	2937	1	0,02	No hits found
scal04231	2938	3	0,02	unknown protein
scal04119	2939	2	0,02	conserved hypothetical protein
scal00169	2940	5	0,02	hypothetical protein
scal03970	2941	2	0,02	similar to multidrug efflux transporter AcrB
scal04250	2942	6	0,02	No hits found
orf06570	2943	1	0,02	No hits found
orf02962	2944	1	0,02	conserved hypothetical protein
scal00148	2945	1	0,02	putative cytochrome b protein of putative nrf cluster
scal04046	2946	2	0,02	similar to biotin-[acetyl-CoA-carboxylase] ligase
scal00741	2947	1	0,02	unknown protein
orf00836	2948	1	0,02	hypothetical protein N47_D31740
scal04050	2949	2	0,02	putative signal-transduction protein with CBS domains
scal00509	2950	2	0,02	Protein of unknown function DUF132
scal02342	2951	13	0,02	conserved hypothetical protein
scal04014	2952	4	0,02	Transposase, IS4 family
scal00885	2953	2	0,02	hypothetical protein
scal00699	2954	1	0,02	unknown protein
scal01149	2954	1	0,02	hypothetical cytosolic protein
scal04085	2956	1	0,02	putativen plasmid maintenance protein HigA
scal01663	2957	2	0,02	MSHA biogenesis protein MshO
scal01105	2958	2	0,02	MCP methyltransferase, CheR-type
scal02305	2959	2	0,02	hypothetical protein Tbd_1847
scal02482	2960	2	0,02	strongly similar to isoleucine tRNA synthetase
scal00255	2961	3	0,02	hypothetical protein GbemDRAFT_2771
scal02442	2962	4	0,02	unknown protein
scal02443	2963	2	0,02	unknown protein
scal03368	2964	2	0,02	conserved hypothetical protein
scal04061	2965	1	0,02	No hits found
scal02136	2966	1	0,02	conserved hypothetical protein
scal04173	2967	5	0,02	Glu/Leu/Phe/Val dehydrogenase, C terminal
scal01494	2968	1	0,02	No hits found
scal01263	2969	4	0,02	glycosyl transferase, group 1 family protein
scal00712	2970	1	0,02	conserved hypothetical protein
orf03325	2971	2	0,02	conserved hypothetical signal transduction HD GYP protein
scal00254	2972	15	0,02	Thrombospondin-like
scal01535	2973	1	0,02	No hits found
scal03858	2974	1	0,02	similar to ribonuclease R
orf07381	2975	3	0,02	Na+/H+ antiporter
scal01094	2976	2	0,02	hypothetical protein MED222_06455
scal03337	2977	9	0,02	polysaccharide biosynthesis protein CapD
scal00679	2978	3	0,02	fibronectin type III domain containing protein
scal02199	2979	2	0,02	No hits found
scal03775	2979	1	0,02	hypothetical protein STIAU_4606
scal03330	2981	4	0,02	PUTATIVE GALACTOSYLTRANSFERASE
scal02452	2982	10	0,02	hypothetical protein Cphamn1DRAFT_2606
scal02232	2983	3	0,02	hypothetical protein Fjoh_4830
scal00543	2984	1	0,02	No hits found

scal01808	2985	2	0,02	No hits found
scal02639	2986	1	0,02	No hits found
scal01453	2987	2	0,02	putative peptidyl-prolyl cis-trans isomerase
orf07443	2988	3	0,02	No hits found
scal02473	2989	4	0,02	conserved hypothetical protein
scal02251	2990	2	0,02	No hits found
scal03902	2990	3	0,02	No hits found
scal00570	2992	1	0,02	unknown protein
scal02137	2993	1	0,02	unknown protein
scal00461	2994	10	0,02	putative multiheme protein with 4 cxxch and 1 cxxxxch and trp repeat motives
orf04704	2995	1	0,02	No hits found
scal03596	2996	1	0,02	No hits found
scal03467	2997	6	0,02	conserved hypothetical protein
scal03875	2998	4	0,02	nucleotidyltransferase substrate binding protein, HI0074 family
scal03282	2999	1	0,02	Response regulator receiver:Transcriptional regulatory protein, C-terminal
scal02441	3000	2	0,02	hypothetical protein PTH_1903
scal03941	3001	3	0,02	virulence associated protein
scal02292	3002	2	0,02	putative TetR cog1309 transcriptional regulator
scal01143	3003	4	0,02	No hits found
scal01746	3004	1	0,02	No hits found
scal03988	3005	4	0,02	conserved hypothetical protein
scal01795	3006	8	0,02	glutamate-1-semialdehyde aminomutase
scal01104	3007	2	0,02	response regulator receiver modulated CheB methylesterase
scal02474	3008	2	0,02	No hits found
scal00802	3009	2	0,02	hypothetical protein CCC13826_1210
scal02618	3010	2	0,02	unknown protein containing 1 cxxch motive
scal03447	3011	1	0,02	hypothetical protein SYN_01583
scal01042	3012	1	0,02	No hits found
scal02230	3013	6	0,02	hypothetical protein PM8797T_16308
scal01621	3014	2	0,02	hypothetical protein
scal03550	3015	5	0,02	NA(+)/H(+) antiporter 1
scal03432	3016	2	0,02	TonB family protein
scal02165	3017	1	0,02	N-acylglucosamine 2-epimerase
scal02503	3018	1	0,02	similar to adenylylsulfate reductase chain B
orf05611	3019	1	0,02	No hits found
scal02700	3020	2	0,02	hypothetical protein MTH1105
scal00054	3021	1	0,02	pilin assembly protein
scal00832	3022	2	0,02	No hits found
scal00484	3023	5	0,02	predicted orf
scal00044	3024	2	0,02	putative sodium dependent transporter
scal00407	3025	1	0,02	conserved hypothetical protein
orf04595	3026	1	0,02	strongly similar to methylated-DNA-protein-cysteine S-methyltransferase
scal03944	3027	1	0,02	No hits found
scal03956	3028	3	0,02	hypothetical protein JJD26997_0348
scal01996	3029	3	0,02	hypothetical protein DoleDRAFT_2806
scal01593	3030	2	0,02	hypothetical protein Acid_2941
orf04641	3031	1	0,02	conserved hypothetical protein
scal01796	3032	2	0,02	DegT/DnrJ/EryC1/StrS aminotransferase
scal01782	3033	2	0,02	No hits found

scal02195	3033	2	0,02	No hits found
orf07086	3035	1	0,02	Conserved hypothetical protein
scal00247	3036	4	0,02	RlpA-like lipoprotein
scal03196	3037	2	0,02	No hits found
scal03117	3038	1	0,02	putative cytochrome c protein with 1 cxxch motive
orf06780	3039	1	0,02	No hits found
orf06920	3039	1	0,02	No hits found
scal03876	3041	2	0,02	DNA polymerase, beta-like region
scal01415	3042	4	0,00	conserved hypothetical protein
scal02126	3043	4	0,00	unknown protein
orf07515	3043	1	0,00	resolvase-like protein
scal01842	3045	2	0,00	putative hydrolase, alpha/beta fold family
scal03334	3046	1	0,00	Sugar transferase involved in lipopolysaccharide synthesis-like
scal03698	3047	1	0,00	cAMP-dependent transcriptional regulator
scal03753	3048	5	0,00	NADH dehydrogenase-like
scal03561	3049	1	0,00	No hits found
scal02120	3050	2	0,00	unknown protein
scal00806	3051	2	0,00	No hits found
scal04238	3052	2	0,00	response regulator protein
scal03658	3053	1	0,00	unknown protein
scal03628	3054	2	0,00	conserved hypothetical protein (NAPRTase)
scal00129	3055	1	0,00	response regulator protein
scal03197	3056	3	0,00	hypothetical protein STIAU_8011
scal03624	3057	3	0,00	type I DNA restriction-modification system
scal00189	3058	2	0,00	conserved hypothetical protein
scal03351	3059	2	0,00	putative potassium channel protein
scal02789	3060	1	0,00	unknown protein
scal01505	3061	2	0,00	No hits found
scal02189	3062	2	0,00	conserved hypothetical sensor protein with pas domain
orf07226	3063	1	0,00	No hits found
scal02213	3064	2	0,00	CRISPR-associated protein, Csm2 family
scal03874	3065	14	0,00	protein of unknown function DUF450
scal00526	3066	2	0,00	hypothetical DUF1568 and COG1943 Transposase like protein
scal04266	3067	1	0,00	No hits found
orf06406	3068	2	0,00	No hits found
scal01052	3069	2	0,00	6-phosphofructokinase
orf02926	3070	1	0,00	putative 6-glucosyltransferase, TobM2
scal01366	3071	5	0,00	lipolytic enzyme, G-D-S-L family
scal02396	3072	2	0,00	Radical SAM
orf01601	3073	1	0,00	heat shock protein Hsp20
scal00470	3074	1	0,00	Hypothetical protein
scal00813	3075	4	0,00	hypothetical protein with 1 cxxch motive
orf05359	3076	1	0,00	No hits found
scal01655	3077	1	0,00	hypothetical DUF1568 and COG1943 Transposase like protein
scal03855	3078	3	0,00	putative carbamoyl transferase, NodU family protein
scal03969	3079	1	0,00	hypothetical protein
scal00469	3080	3	0,00	glycosyl transferase, family 2
scal03475	3081	2	0,00	Os07g0172900
orf06034	3082	1	0,00	No hits found

scal02555	3083	7	0,00	conserved hypothetical protein, membrane
scal01443	3084	1	0,00	No hits found
scal01534	3085	1	0,00	Protein of unknown function DUF1016
orf03420	3086	1	0,00	hypothetical protein HRM2_19750
scal00767	3087	12	0,00	BNR repeat domain protein
orf06771	3088	1	0,00	Excinuclease ABC C subunit domain-containing protein
scal03462	3089	3	0,00	conserved hypothetical dynamin like protein
scal03939	3090	1	0,00	Type II secretory pathway, component PulF
orf07001	3091	1	0,00	conserved hypothetical protein
scal00646	3092	1	0,00	hypothetical protein MA2996
scal01880	3093	1	0,00	hypothetical protein Cag_1197
scal01323	3094	2	0,00	UDP-N-acetylglucosamine 4-epimerase
scal00725	3095	1	0,00	putative phospholipid-binding protein
scal01421	3096	10	0,00	hypothetical protein
scal00204	3097	1	0,00	putative cyanate hydratase
scal01894	3098	1	0,00	No hits found
scal00905	3099	1	0,00	conserved hypothetical protein
scal03524	3100	6	0,00	metallophosphoesterase
scal01242	3101	5	0,00	sulfate transport protein
scal00500	3102	1	0,00	protein of unknown function DUF497
scal03828	3103	3	0,00	putative DNA methylase
scal03242	3104	9	0,00	similar to Succinoglycan biosynthesis transport protein
scal03258	3105	1	0,00	No hits found
scal03896	3106	2	0,00	hypothetical protein
scal00264	3107	1	0,00	conserved hypothetical protein
scal00088	3108	1	0,00	conserved hypothetical protein
scal02868	3109	1	0,00	hypothetical protein Adeh_1035
scal01753	3110	1	0,00	hypothetical protein N9414_24063
scal01709	3111	1	0,00	Response regulator receiver:Metal-dependent phosphohydrolase, HD subdomain
scal02669	3112	1	0,00	putative DNA methylase
scal00340	3113	1	0,00	No hits found
scal00819	3114	1	0,00	No hits found
scal01617	3115	5	0,00	putative glycosyl transferase
scal01855	3116	3	0,00	hypothetical protein HCH_03629
scal01688	3117	2	0,00	hypothetical protein Ppro_3557
scal03954	3118	2	0,00	No hits found
scal00417	3119	1	0,00	conserved hypothetical membrane protein with signal transduction CBS domain
scal03671	3120	1	0,00	No hits found
scal00098	3121	1	0,00	capsular polysaccharide export protein, putative
scal03606	3122	2	0,00	viral A-type inclusion protein, putative
scal01694	3123	1	0,00	type II secretion system protein, GspI
scal03718	3124	1	0,00	polysaccharide export protein
scal02957	3125	2	0,00	unkown protein
scal02874	3126	2	0,00	3-oxoacyl acyl-carrier protein reductase
scal03510	3126	1	0,00	No hits found
scal01468	3128	1	0,00	putative potassium channel protein
scal01432	3129	1	0,00	nucleotide-binding protein
scal03894	3130	1	0,00	unknown protein
scal04211	3131	1	0,00	hypothetical protein CPS_2373

scal01690	3132	1	0,00	No hits found
scal03292	3133	2	0,00	putative iron sulfur cysteine desulfhydrase protein
scal03445	3134	2	0,00	putative methyltransferase
scal02522	3135	1	0,00	response regulator receiver protein
scal02507	3136	1	0,00	hypothetical protein Mboo_1602
scal03659	3137	1	0,00	unknown protein
scal01717	3138	3	0,00	Helicase, C-terminal:DEAD/DEAH box helicase, N-terminal
scal01683	3139	1	0,00	conserved hypothetical protein
scal02385	3140	2	0,00	putative SAM radical protein
scal02724	3141	2	0,00	No hits found
scal02836	3141	1	0,00	No hits found
scal03125	3143	2	0,00	Signal transduction histidine kinase
scal03176	3144	1	0,00	hypothetical protein lin1966
orf07107	3145	1	0,00	No hits found
scal03290	3146	2	0,00	hypothetical protein Cag_0309
scal02451	3147	1	0,00	No hits found
scal01874	3148	1	0,00	polysaccharide deacetylase
scal00831	3149	1	0,00	putative transport protein
scal00071	3150	1	0,00	hemerythrin-like metal-binding protein
scal01223	3151	1	0,00	hypothetical protein LPC_3225
scal01677	3152	1	0,00	hypothetical protein Csal_1381
scal03141	3153	1	0,00	putative polysaccharide transporter protein
scal02944	3154	6	0,00	hypothetical protein Cag_0856
scal00351	3155	1	0,00	Pyruvate kinase
scal00445	3155	1	0,00	putative histidine kinase
scal03264	3157	1	0,00	hypothetical protein GSU1969
scal02776	3158	1	0,00	hypothetical protein ML2346
scal03126	3158	1	0,00	putative DNA primase
scal03286	3160	2	0,00	No hits found
scal02188	3161	1	0,00	unknown protein
scal02351	3162	1	0,00	conserved hypothetical protein
scal04267	3163	1	0,00	hypothetical protein Gura_2669
scal00652	3164	3	0,00	conserved hypothetical protein
scal01239	3165	1	0,00	protein of unknown function DUF323
scal00575	3166	1	0,00	No hits found
scal00969	3167	1	0,00	DNA mismatch endonuclease vsr
scal00956	3168	1	0,00	similar to Sigma-54 dependent transcriptional regulator
scal01168	3169	1	0,00	putative integral membrane protein
scal03115	3170	2	0,00	Sodium/hydrogen exchanger
scal03327	3171	2	0,00	glycosyltransferase
scal01430	3172	2	0,00	hypothetical protein Tcr_0176
scal01447	3173	4	0,00	putative N-methylhydantoinase A/acetone carboxylase, beta subunit
scal03320	3174	1	0,00	hypothetical protein
scal03050	3175	1	0,00	sodium/hydrogen exchanger
scal03231	3176	1	0,00	hypothetical cog1943 protein
scal00678	3177	1	0,00	conserved hypothetical protein; putative conserved domain
scal02654	3178	5	0,00	unknown anammox protein
scal03457	3179	1	0,00	hypothetical protein
scal00544	3180	1	0,00	ntrC like sigma 54 response regulator

scal04208	3181	4	0,00	TPR Domain containing protein
scal03281	3182	2	0,00	expressed hypothetical protein
scal01414	3183	2	0,00	conserved hypothetical protein
scal01068	3184	2	0,00	DegT/DnrJ/EryC1/StrS aminotransferase
scal02689	3185	4	0,00	dead/deah box helicase, fused to N-terminal hd domain
scal00003	3186	3	0,00	putative response regulator protein
scal03106	3187	2	0,00	No hits found
scal01459	3188	1	0,00	similar to ribonucleoside-diphosphate reductase 1 alpha chain
scal03607	3189	3	0,00	putative type IV pilin biosynthesis protein pilQ
scal01424	3190	9	0,00	peptidyl arginine deiminase, type I
scal03521	3191	6	0,00	hypothetical large protein
scal01365	3192	1	0,00	Methyltransferase type 11
scal00702	3193	1	0,00	conserved hypothetical COG2078 protein
orf05870	3194	2	0,00	No hits found
scal02073	3195	1	0,00	putative cob(I)alamin adenosyltransferase
scal03933	3196	3	0,00	hypothetical tpr repeat protein C terminal
scal02421	3197	1	0,00	putative resA type II cytochrome c biogenesis protein
scal03338	3198	3	0,00	exopolysaccharide biosynthesis family protein
scal04122	3199	1	0,00	No hits found
scal01726	3200	1	0,00	hypothetical protein MldDRAFT_0962
scal03388	3201	3	0,00	NADH dehydrogenase (quinone)
scal02517	3202	1	0,00	similar to serine/threonine protein kinase (catalytic domain)
scal03955	3203	1	0,00	3-oxoacyl acyl-carrier protein reductase
scal02383	3204	1	0,00	glycosyl transferase, group 2 family protein
scal00939	3205	1	0,00	No hits found
scal03662	3206	1	0,00	hypothetical protein ISM_00180
scal02477	3207	1	0,00	unknown protein
scal02696	3208	1	0,00	No hits found
scal01225	3209	1	0,00	No hits found
scal01203	3210	2	0,00	No hits found
scal03001	3211	1	0,00	unkown protein
scal00801	3212	2	0,00	similar to general secretion pathway protein D
scal03996	3213	2	0,00	putative iron sulfur SAM radical heme d biosynthesis protein nirJ
scal00004	3214	2	0,00	putative Tfp pilus assembly protein PilF
scal01615	3215	2	0,00	putative SAM radical protein
scal03823	3216	2	0,00	succinoglycan biosynthesis transport protein
orf05784	3217	1	0,00	dTDP-4-dehydrorhamnose reductase
scal00698	3218	2	0,00	putative transcriptional regulator
scal03378	3219	2	0,00	hypothetical protein PFL_2316
scal02212	3220	1	0,00	CRISPR-associated RAMP protein, Csm3 family
scal00029	3221	1	0,00	hypothetical protein plu1934
scal03096	3222	5	0,00	hypothetical protein Fjoh_3158
scal03162	3223	1	0,00	ABC-2 type transporter
scal00249	3224	1	0,00	hypothetical lipoprotein
scal03249	3224	1	0,00	hypothetical protein mlr5887
scal02655	3226	1	0,00	putative molecular chaperone protein grpE
scal01821	3227	4	0,00	hypothetical protein Noc_1846
scal01044	3228	2	0,00	Signal transduction histidine kinase-like protein
scal02985	3229	1	0,00	hypothetical protein

scal01895	3230	1	0,00	diguanylate cyclase
scal01065	3231	1	0,00	SAM radical protein
scal04263	3232	2	0,00	putative permease
scal01418	3233	2	0,00	hypothetical phage like protein
scal03089	3234	2	0,00	COG1674: DNA segregation ATPase FtsK/SpoIIIE and related proteins
scal01718	3235	1	0,00	hypothetical protein CaulDRAFT_1646
scal00463	3236	1	0,00	RNA-directed DNA polymerase (Reverse transcriptase
scal03130	3237	1	0,00	hypothetical protein Noc_0059
scal03128	3238	1	0,00	No hits found
scal03272	3239	1	0,00	N-acetylneuraminate synthase
scal00122	3240	1	0,00	hypothetical protein SC2700
scal01898	3241	1	0,00	ATP-binding region, ATPase-like
scal00524	3242	2	0,00	putative clp protease ATP binding subunit
scal00471	3243	1	0,00	putative YdjC family protein
scal02557	3244	1	0,00	similar to cofactor modifying protein
scal02619	3245	1	0,00	unknown protein
scal04264	3246	1	0,00	putative SAM radical protein
scal02730	3247	1	0,00	ATP-binding region, ATPase-like
scal03685	3247	1	0,00	No hits found
scal00807	3249	1	0,00	No hits found
scal01756	3250	1	0,00	hypothetical protein Tcr_1744
scal02984	3251	1	0,00	putative histidine kinase
scal00154	3252	1	0,00	hypothetical protein
scal02149	3253	1	0,00	putative transcriptional regulator
scal02995	3254	1	0,00	type II secretion system protein E
scal03400	3255	2	0,00	type II secretion system protein E
scal00987	3256	1	0,00	NAD-dependent dehydrogenase, hydrogenase, component C-formate hydrogenlyase
scal01164	3257	1	0,00	hypothetical protein
scal02766	3258	1	0,00	glycosyltransferase (family 2)
scal03574	3259	1	0,00	No hits found
scal02767	3260	1	0,00	dolichol-phosphate mannosyltransferase
scal00872	3261	1	0,00	conserved hypothetical protein
scal03730	3262	2	0,00	similar to asparagine synthetase
scal03048	3263	2	0,00	virulence associated protein
scal01590	3264	2	0,00	Cation transport ATPase
scal01867	3265	2	0,00	putative ribonucleoside reductase
scal01823	3266	1	0,00	No hits found
scal03251	3267	1	0,00	glycosyltransferase group 2 family protein
scal03940	3268	1	0,00	putative histidine kinase
scal02063	3269	1	0,00	putative glycosyltransferase protein
scal02208	3270	1	0,00	No hits found
scal02713	3271	1	0,00	putative Sel1-like repeat protein
scal00918	3272	1	0,00	hypothetical protein RD1_3848
scal02214	3273	2	0,00	CRISPR-associated protein, Csm1 family
scal02679	3274	1	0,00	hypothetical protein RSP_3752
scal01363	3275	1	0,00	hypothetical protein PPSIR1_26258
scal03888	3276	4	0,00	cell division protein FtsK
scal00354	3277	1	0,00	putative sucrose phosphorylase
scal03404	3278	1	0,00	similar to transposase istA

scal03367	3279	1	0,00	No hits found
scal03329	3280	1	0,00	hypothetical cytosolic protein
scal03638	3281	1	0,00	Secretion protein HlyD
scal01785	3282	1	0,00	hypothetical protein HCH_06140
scal04076	3283	1	0,00	putative flavoprotein norVW
scal01397	3284	1	0,00	putative N-methylhydantoinase / 5 oxoprolinase
scal03276	3285	1	0,00	DegT/DnrJ/EryC1/StrS family protein
scal02973	3286	1	0,00	hypothetical protein Plav_0314
scal01391	3287	3	0,00	putative two component sensor kinase
scal02726	3288	1	0,00	virulence associated protein
scal00369	3289	2	0,00	hypothetical protein RPC_3546
scal01198	3290	1	0,00	O-antigen polymerase
scal02384	3291	2	0,00	Radical SAM domain protein
scal01704	3292	1	0,00	Type IV pilus assembly protein PilM
scal03364	3293	1	0,00	ABC transporter-like
scal01336	3294	1	0,00	outer membrane efflux protein
scal01616	3295	1	0,00	putative 4 amino butyrate aminotransferase
scal00110	3296	1	0,00	Metallophosphoesterase
scal04008	3297	1	0,00	conserved hypothetical COG4188 protein; putative dienelactone hydrolase
scal00805	3298	2	0,00	adenine specific DNA methyltransferase
scal01863	3299	1	0,00	two component, sigma54 specific, transcriptional regulator, Fis family
scal04168	3300	1	0,00	response regulator receiver modulated metal dependent phosphohydrolase
scal04289	3301	1	0,00	strongly similar to sigma 54 response regulatory protein
scal00834	3302	1	0,00	unknown protein
scal00084	3303	1	0,00	major facilitator superfamily MFS_1
scal03020	3304	2	0,00	putative helicase subunit of dna repair complex
scal02210	3305	1	0,00	CRISPR-associated RAMP protein, Csm5 family
scal01800	3306	1	0,00	hypothetical protein Mbur_1491
scal03710	3307	1	0,00	hypothetical protein NE0256
scal04290	3308	1	0,00	similar to two component sensor histidine kinase
scal04015	3309	1	0,00	hypothetical protein MA4278
scal02552	3310	1	0,00	Predicted Fe-S oxidoreductase
scal01778	3311	1	0,00	No hits found
scal01635	3312	1	0,00	hypothetical protein
scal04291	3313	1	0,00	Unknown protein
scal02462	3314	1	0,00	unknown duf87 protein
scal03093	3315	1	0,00	hypothetical protein all3313
scal02693	3316	1	0,00	conserved hypothetical protein, membrane
scal03091	3317	1	0,00	hypothetical protein
scal00039	3318	1	0,00	hypothetical protein Patl_3452
scal00113	3319	1	0,00	putative type-2 Multicopper oxidase
scal04300	3320	1	0,00	hypothetical protein
scal02446	3321	1	0,00	helicase subunit of Type III like restriction-modification enzyme
scal03248	3322	1	0,00	No hits found
scal01897	3323	1	0,00	hypothetical protein bll8177
scal00030	3324	1	0,00	hypothetical protein Noc_1367
scal00021	3325	1	0,00	similar to glucoamylase
scal03918	3326	1	0,00	hypothetical protein
scal00917	3327	1	0,00	similar to asparagine synthetase

scal03950	3328	1	0,00	predicted ATPase invovled in DNA repair
scal03103	3329	1	0,00	putative phage tail sheath protein FI
scal02097	3330	1	0,00	translation elongation factor G
scal01372	3331	1	0,00	hypothetical protein Ppro_2465
scal02296	3332	4	0,00	YD repeat protein
scal03492	3333	1	0,00	hypothetical protein
scal03255	3334	1	0,00	putative glycosyl transferase
scal00777	3335	1	0,00	hypothetical protein
scal00527	3336	1	0,00	putative WD-40 repeat protein
scal02051	3337	1	0,00	hypothetical protein PM8797T_02194
scal00107	3338	1	0,00	DNA methylase N-4/N-6 domain protein
scal01413	3339	1	0,00	hypothetical protein
scal00462	3340	1	0,00	ASPIC/UnbV domain protein
scal00835	3341	4	0,00	hypothetical large PKD domain protein
scal03829	3342	1	0,00	putative helicase
scal02728	3343	1	0,00	hypothetical protein Noc_0059
scal03094	3344	1	0,00	hypothetical protein GbemDRAFT_0310
scal03366	3345	1	0,00	phosphoenolpyruvate synthetase-related protein
scal01691	3346	1	0,00	unknown protein
scal03408	3347	1	0,00	beta-ketoacyl synthase
