# Supplementary material for: The metagenome of the marine anammox bacterium ‘Candidatus Scalindua profunda’ illustrates the versatility of this globally important nitrogen cycle bacterium
Source: Environ Microbiol. 2013 May;15(5):1275–89. doi: 10.1111/j.1462-2920.2012.02774.x (PMC3655542; doi:10.1111/j.1462-2920.2012.02774.x)
Supplement: Supplementary file 12 [file emi0015-1275-SD12.pdf]

Name	Observable peptides	Peptides	Rank Peptides	emPAI	Annotation
scal00863	106	54	1	5,1	nitrate reductase subunit NarG
scal00880	344	44	2	0,4	large tpr repeat protein
scal02278	117	37	3	1,1	DNA-directed RNA polymerase, beta prime subunit
scal01318	66	33	4	5,4	putative diheme protein of hydrazine synthase cluster
scal00025	60	29	5	3,5	expressed hypothetical (diheme) protein of hydrazine synthase cluster
scal02798	49	28	6	3,9	chaperonin GroEL
scal04035	76	28	6	1,5	unknown protein
scal02098	54	27	8	2,8	nirS cd1 nitrite reductase
scal02279	108	27	8	0,8	DNA-directed RNA polymerase, beta subunit
scal00880	179	26	10	0,4	large tpr repeat protein
scal00944	56	25	11	1,9	chaperone Hsp70
scal03295	49	23	12	5,6	CxxxxCH containing hydrazine oxidase (hzo)
scal00670	42	23	12	3,9	60 kDa chaperonin (groEL protein)
scal00635	49	20	14	1,7	expressed chaperonin GroEL
scal00659	94	20	14	0,6	expressed FAD-dependent pyridine nucleotide-disulphide oxidoreductase
scal00867	45	19	16	3,4	nitrate reductase subunit NarH
scal03847	80	19	16	0,9	putative protein-export membrane protein SecD
scal02275	68	19	16	0,9	translation elongation factor EF-G
scal00215	112	18	19	0,4	expressed pyruvate:ferredoxin oxidoreductase
scal04193	47	18	19	1,4	30S ribosomal protein S1
scal02521	54	17	21	1,4	formyltetrahydrofolate synthetase
scal01210	62	17	21	0,9	polynucleotide phosphorylase (PNPase)
scal00419	22	16	23	10,1	expressed putative PAS/PAC sensor protein
scal02752	38	16	23	2,2	heat shock protease DegP/HtrA
scal02116	47	16	23	1,5	hydroxylamine oxidoreductase hao
scal00198	40	16	23	1,5	expressed alpha subunit of F0F1 ATP synthase
scal02795	40	15	27	2,2	chaperonin GroEL
scal02427	40	15	27	1,7	expressed conserved hypothetical protein
scal03380	100	15	27	0,4	COG2303: Choline dehydrogenase and related flavoproteins
scal02758	96	15	27	0,5	putative protease
scal02468	70	15	27	0,6	expressed hypothetical protein
scal03516	39	15	27	1,4	nucleoside diphosphate kinase 4
scal00421	57	14	33	1,2	expressed hydroxylamine oxidoreductase hao cluster 4
scal02425	43	14	33	1,4	putative tpr repeat protein
scal03840	43	14	33	1,2	putative DNA directed RNA polymerase
scal01487	46	13	36	1,1	putative glutamate synthase
scal00196	46	13	36	1,0	expressed F1F0 ATP synthase beta subunit
scal00072	45	13	36	1,0	expressed S-adenosyl-L-homocysteine hydrolase
scal02133	56	13	36	0,7	bifunctional purine biosynthesis protein phosphoribosylaminoimidazolecarboxamide formyltransferase (AICAR transformylase); IMP cyclohydrolase
scal01317	28	12	40	6,2	hydroxylamine oxidoreductase hao
scal00418	32	12	40	2,7	expressed putative PAS/PAC sensor protein
scal00943	80	12	40	0,4	ATP-dependent protease

scal04034	47	12	40	0,8	putative ABC type transport protein, auxiliary component
scal02935	29	11	44	1,6	unknown protein COG3945
scal02930	49	11	44	0,7	expressed hypothetical protein
scal02417	60	11	44	0,5	pyruvate carboxylase, beta chain
scal00961	57	11	44	0,6	putative ABC-type transport protein involved in gliding motility
scal02020	54	11	44	0,6	partial acetyl-coa synthetase, acetate coa ligase AMP forming
scal03169	14	10	49	7,5	heat shock protein (Hsp17)
scal04164	41	10	49	1,0	hydroxylamine oxidoreductase hao
scal00315	32	10	49	1,2	expressed 3-isopropylmalate dehydrogenase leuB
scal01642	58	10	49	0,5	similar to proton-translocating NADH dehydrogenase I, 51 kDa subunit (NuoF)
scal02426	45	10	49	0,8	putative tpr repeat protein
scal03545	58	10	49	0,5	heat shock protein Hsp90
scal00582	38	10	49	0,8	expressed putative membrane protease
scal02524	20	9	56	4,0	unknown protein of narGH cluster
scal01402	31	9	56	1,3	strongly similar to hydroxymethylbilane synthase (porphobilinogen deaminase)
scal02486	39	9	56	0,8	large subunit corrinoid FeS protein of CODH/ACS complex(ascC)
scal01099	18	9	56	2,6	putative outer membrane chaperone OmpH
scal01299	38	9	56	0,7	putative aspartate aminotransferase
scal02398	32	9	56	0,9	ketol-acid reductoisomerase
scal02659	28	8	62	1,5	S-adenosylmethionine synthetase
scal02490	28	8	62	1,3	5-methyltetrahydrofolate/corrinoid Fe-S protein methyltransferase (acsE) of the CODH/ACS complex
scal03518	73	8	62	0,3	DNA gyrase subunit A, type II topoisomerase
scal02134	27	8	62	1,2	putative membrane-bound lytic murein transglycosylase A
scal02814	23	8	62	1,5	unknown conserved protein
scal02110	54	8	62	0,4	putative hydroxylamine oxidoreductase hao with 8 heme / cxxch motives
scal04039	42	8	62	0,6	SAM radical protein; putaive cobalamin b12 binding
scal02055	60	8	62	0,4	GltD like FAD containing NAD(P) oxidoreductase
scal02494	58	8	62	0,4	Phosphoenolpyruvate carboxykinase (GTP)
scal00581	35	8	62	0,7	expressed putative membrane protease
scal03187	38	8	62	0,6	putative membrane protease
scal00435	39	8	62	0,6	expressed putative PpiC-type peptidyl-prolyl cis-trans isomerase protein
scal00657	27	8	62	1,0	expressed putative nickel insertase (cooC) of CODH/ACS complex
scal01127	82	8	62	0,3	strongly similar to negative regulator of genetic competence ClpC/MecB
scal00686	96	8	62	0,2	putative multiheme protein containing 8 heme/ cxxch motives
scal00864	35	8	62	0,7	unknown protein
scal04256	44	8	62	0,6	expressed hypothetical protein
scal04115	7	7	79	18,3	putative monoheme protein cytochrome c553
scal03191	33	7	79	0,7	glyceraldehyde-3-phosphate dehydrogenase
scal03352	62	7	79	0,3	hypothetical protein
scal01005	35	7	79	0,7	DNA-directed RNA polymerase, alpha chain
scal02822	23	7	79	1,2	unknown protein
scal03300	38	7	79	0,6	aspartate aminotransferase AspC
scal04175	20	7	79	1,5	thioredoxin peroxidase
scal00409	24	7	79	1,0	expressed fructose-6-phosphate aldolase
scal02611	35	7	79	0,6	surA peptidyl-prolyl cis-trans isomerase
scal02132	84	7	79	0,2	phosphoenolpyruvate synthase/ pyruvate phosphate dikinase
scal03745	83	7	79	0,2	Leucyl-tRNA synthetase bacterial/mitochondrial, class Ia
scal03186	33	7	79	0,6	expressed conserved hypothetical protein
scal00434	47	7	79	0,4	expressed aminopeptidase A
scal01643	74	7	79	0,2	similar to molybdopterin oxidoreductase, molybdopterin-containing subunit/ NuoG subunit of NADH dehydrogenase I

scal00326	58	7	79	0,3	expressed aspartyl-tRNA ligase aspS
scal03386	70	7	79	0,3	No hits found
scal04326	55	7	79	0,3	alpha-glucan phosphorylase
scal01286	68	7	79	0,3	NAD(P) oxidoreductase, FAD-containing subunit, 4Fe-4S ferredoxin, iron-sulfur binding
scal02091	49	7	79	0,4	putative iron sulfur NuoF subunit of NADH:ubiquinone oxidoreductase
scal02325	61	7	79	0,3	expressed conserved hypothetical protein BatD
scal04146	35	7	79	0,6	succinyl-CoA synthetase, beta subunit
scal02174	18	7	79	1,4	putative outer membrane chaperone OmpH
scal04116	11	6	101	9,0	expressed hypotheticial monoheme protein
scal00692	25	6	101	1,6	putative tetraheme protein
scal03379	75	6	101	0,2	putative COG2303 Choline dehydrogenase and related flavoproteins
scal03179	70	6	101	0,3	TPR Domain containing protein
scal04218	35	6	101	0,6	conserved hypothetical protein of unknown function (DUF1432)
scal02111	48	6	101	0,4	putative cell wall associated protein
scal02282	22	6	101	1,1	50S ribosomal protein L1
scal02065	26	6	101	0,9	fructose-1,6-biphosphate aldolase Class I
scal00687	18	6	101	1,4	conserved hypothetical protein of nar cluster
scal02280	12	6	101	2,8	50S ribosomal protein L7/L12
scal04102	45	6	101	0,4	ATP-dependent RNA helicase
scal01014	14	6	101	1,7	30S ribosomal protein S5
scal01152	50	6	101	0,3	uroporphyrinogen III synthase/methyltransferase
scal01820	79	6	101	0,2	SNF2-related:Helicase-like:SWIM Zn-finger:DEAD/DEAH box helicase-like
scal03306	75	6	101	0,2	strongly similar to DNA gyrase subunit B
scal02371	40	6	101	0,4	similar to trigger factor (TF)
scal02661	90	6	101	0,2	SecA subunit of the preprotein translocase
scal00786	50	6	101	0,3	V-type H(+)-translocating pyrophosphatase
scal03502	125	6	101	0,1	N terminal part of putative coenzyme a (CoA) enzyme activase
scal02797	10	6	101	3,0	chaperonin GroES
scal01013	12	6	101	2,2	50S ribosomal protein L15
scal01024	16	6	101	1,4	30S ribosomal protein S3
scal02628	38	6	101	0,4	aldehyde dehydrogenase
scal01133	58	6	101	0,3	D-3-phosphoglycerate dehydrogenase
scal02333	69	6	101	0,2	4Fe-4S ferredoxin, iron-sulfur binding:FAD dependent oxidoreductase
scal00430	36	6	101	0,5	similar to molybdenum cofactor biosynthesis protein
scal02178	22	6	101	0,9	30S ribosomal protein S2
scal00631	22	6	101	0,9	expressed ompA/motB flagellar motor protein
scal03783	23	6	101	0,8	expressed conserved hypothetical protein
scal01729	18	6	101	1,2	Putative translation initiation inhibitor
scal04112	47	6	101	0,3	conserved hypopethical protein
scal00515	24	6	101	0,8	3-oxoacyl acyl-carrier protein reductase
scal03181	24	6	101	1,0	hypothetical protein
scal03083	18	5	134	1,4	PKD domain protein
orf07451	6	5	134	20,5	hypothetical protein CLOHYLEM_03944
scal00868	22	5	134	1,3	putative narM like gamma subunit nitrate reductase
scal00418	12	5	134	2,7	expressed putative PAS/PAC sensor protein
scal03294	28	5	134	0,8	Methenyltetrahydrofolate cyclohydrolase
scal00866	17	5	134	1,6	unknown protein
scal01018	11	5	134	2,5	50S ribosomal protein L5
scal00595	9	5	134	3,6	expressed nitrogen regulatory protein P-II
scal02489	31	5	134	0,6	small subunit of corrinoid FeS protein of the CODH/ACS complex (acsD)

scal01408	12	5	134	2,2	50S ribosomal protein L19
scal03051	24	5	134	0,8	CO dehydrogenase/acetyl-CoA synthase alpha subunit acsB
scal01386	40	5	134	0,4	argininosuccinate synthetase
scal01006	19	5	134	1,1	30S ribosomal protein S4
scal00237	55	5	134	0,3	strongly similar to transcription initiation factor sigma RpoD
scal03016	27	5	134	0,7	nitrite/sulfite reductase protein, NirB
scal04318	6	5	134	9,0	cold shock protein A
scal01031	10	5	134	2,2	30S ribosomal protein S10
scal01543	15	5	134	1,2	putative flavoprotein norVW
scal02167	19	5	134	0,8	50S ribosomal protein L25
scal02107	19	5	134	0,8	qcrB cytochrome b6
scal02911	44	5	134	0,3	hypothetical protein
scal02910	81	5	134	0,2	strongly similar to valyl-tRNA synthetase
scal00401	66	5	134	0,2	hypothetical protein
scal04036	32	5	134	0,4	expressed conserved hypothetical protein with MoxR-like ATPase domain
scal00094	50	5	134	0,3	expressed protein with unknown function
scal02080	14	5	134	1,3	expressed hypothetical protein
scal03168	60	5	134	0,2	Methyltransferase type 11
scal02179	20	5	134	0,8	elongation factor Ts (EF-Ts)
scal00362	59	5	134	0,2	strongly similar to 1-deoxy-D-xylulose 5-phosphate synthase (DXP synthase)
scal03192	42	5	134	0,3	phosphoglycerate kinase
scal00994	36	5	134	0,4	putative nirJ heme d1 biosynthesis protein
scal04032	30	5	134	0,5	putative ABC type transport protein, ATPase component
scal03075	30	5	134	0,5	cysteine synthase
scal02715	32	5	134	0,4	thioredoxin
scal02173	70	5	134	0,2	putative outer membrane protein Omp85
scal03009	52	5	134	0,2	putative histidine kinase
scal02052	34	5	134	0,4	putative multiheme cytochrome c protein with 5 cxxch motives and 1 cxxxxxch motive
scal02114	34	5	134	0,4	CO dehydrogenase/acetyl-coA synthase beta subunit (acsA)partial
scal02576	31	5	134	0,4	branched-chain amino acid transferase
scal01012	30	5	134	0,5	preprotein translocase secY protein N terminus
scal00272	47	5	134	0,3	expressed D-3-phosphoglycerate dehydrogenase
scal00616	34	5	134	0,4	conserved hypothetical protein
scal01301	47	5	134	0,3	catalase
scal02810	47	5	134	0,3	6-phosphogluconate dehydrogenase
scal00825	48	5	134	0,3	conserved hypothetical protein
scal02400	17	5	134	1,0	strongly similar to acetolactate synthase regulatory subunit
scal02487	51	5	134	0,3	putative iron sulfur / metal binding protein of the CODH/ACS complex
scal01026	23	5	134	0,6	50S ribosomal protein L2
scal00274	20	5	134	0,8	expressed flavoredoxin
scal04293	19	5	134	0,8	putative cytochrome c protein C2
scal00690	18	5	134	0,9	putative monoheme protein
scal02337	30	5	134	0,5	putative iron sulfur heterodisulfide reductase gamma subunit
scal00691	54	5	134	0,3	putative diheme protein
scal00692	27	5	134	1,6	putative tetraheme protein
scal04322	5	4	188	14,8	DNA-binding protein HU-beta
scal04254	14	4	188	1,7	similar to low molecular weight heat shock protein (Hsp17)
scal02632	21	4	188	0,9	hypothetical (triheme) protein of hydrazine synthase cluster with 3 cxxch and 1 cxch mottive
scal01536	7	4	188	6,2	thioredoxin
scal02054	17	4	188	1,3	putative qcrB cytochrome b6 of bc1 complex

scal04154	10	4	188	2,2	PTS system, fructose-specific enzyme II, BC component
scal01640	39	4	188	0,3	putative NADH:ubiquinone oxidoreductase, hydrogenase, component E-formate hydrogenlysase
scal02548	34	4	188	0,4	formate dehydrogenase alpha subunit
scal01287	30	4	188	0,5	5,10-methylenetetrahydrofolate reductase
scal02428	34	4	188	0,4	putative multiheme cytochrome c protein with 11 cxxch motives
scal00995	38	4	188	0,4	aspartate transaminase
scal01297	29	4	188	0,5	3-deoxy-D-arabino-heptulosonate 7-phosphate synthase
scal04230	14	4	188	1,3	unknown protein
scal01488	23	4	188	0,6	TENA/PQQ biosynthesis protein C involved in enhancing extracellular expression
scal00998	13	4	188	1,4	ISxac3 transposase
scal02931	76	4	188	0,1	DNA topoisomerase I
scal02889	35	4	188	0,3	conserved hypothetical HD KH hydrolase protein
scal01009	10	4	188	1,5	30S ribosomal protein S13
scal04110	42	4	188	0,2	seryl-tRNA synthetase
scal01025	11	4	188	1,3	50S ribosomal protein L22
scal02484	17	4	188	0,7	CO dehydrogenase/acetyl-CoA synthase alpha subunit acsB C-terminal
scal04141	46	4	188	0,2	phosphoglyceromutase
scal00170	16	4	188	0,8	expressed PKD domain protein
scal02406	30	4	188	0,4	3-oxoacyl[acyl-carrier protein] synthase III
scal02281	10	4	188	1,5	50S ribosomal protein L10
scal01163	118	4	188	0,1	smc chromosome partition ATPase
scal04153	70	4	188	0,1	strongly similar to phosphoenolpyruvate-protein phosphotransferase (phosphotransferase system, enzyme I)
scal01194	41	4	188	0,3	ATP-dependent Hsl protease
scal02887	45	4	188	0,2	transcription termination factor Rho
scal00634	44	4	188	0,2	adenylosuccinate lyase purB
scal02397	40	4	188	0,3	strongly similar to 2-isopropylmalate synthase
scal02735	26	4	188	0,4	expressed conserved hypothetical protein
scal02099	34	4	188	0,3	SAM radical nirJ like heme d synthesis protein
scal02405	27	4	188	0,4	fatty acid/phospholipid synthesis protein PlsX
scal02276	7	4	188	2,7	30S ribosomal protein S7
scal02240	47	4	188	0,2	putative Zn-dependent protease
scal01489	45	4	188	0,2	hypothetical protein MJ1479
scal02186	21	4	188	0,6	unknown protein
scal03650	6	4	188	3,6	50S ribosomal protein L20
scal02574	45	4	188	0,2	strongly similar to lysyl-tRNA synthetase
scal02171	20	4	188	0,6	50S ribosomal protein L9
scal00193	42	4	188	0,2	similar to diaminopimelate decarboxylase lysa
scal03863	41	4	188	0,3	thiamine biosynthesis protein ThiC
scal02346	39	4	188	0,3	putative glycosyltransferase involved in cell wall biogenesis
scal02833	43	4	188	0,2	glutamyl-tRNA synthetase
scal02376	29	4	188	0,4	acetohydroxy acid synthase IilvB
scal03912	48	4	188	0,2	hypothetical protein
scal00689	20	4	188	0,5	putative diheme protein
scal00415	17	4	188	0,7	expressed putative signal-transduction protein with CBS domains
scal01016	21	4	188	0,6	50S ribosomal protein L6
scal03503	18	4	188	0,7	3-isopropylmalate dehydratase small subunit
scal03756	10	4	188	1,5	small heat shock protein
scal03987	26	4	188	0,4	iron-containing alcohol dehydrogenase
scal04038	16	4	188	0,8	unknown protein
scal03779	19	4	188	0,6	hypothetical protein pc0098

scal01340	31	4	188	0,3	ribokinase protein rbsK
scal00636	6	4	188	3,6	chaperonin GroES
scal00740	30	4	188	0,4	adenylosuccinate synthetase
scal01055	36	4	188	0,3	putative norVW like flavoprotein
scal00691	27	4	188	0,3	putative diheme protein
scal02284	16	3	248	0,8	transcription antitermination protein NusG
scal03897	41	3	248	0,3	strongly similar to inosine-5'-monophosphate dehydrogenase
scal02082	68	3	248	0,1	TPR Domain containing protein
scal01583	24	3	248	0,5	citrate synthase
scal03651	25	3	248	0,4	phenylalanyl-tRNA synthetase alpha chain
scal03254	50	3	248	0,2	putatiave oxidoreductase, Gfo/Idh/MocA family/transferase
scal01400	16	3	248	0,8	peptidyl-prolyl cis-trans isomerase A
scal03910	31	3	248	0,3	4-hydroxy-3-methylbut-2-en-1-yl diphosphate synthase
scal00952	12	3	248	1,2	putative superoxide reductase
scal00197	28	3	248	0,3	expressed gamma subunit of F1F0 ATP synthase
scal03060	19	3	248	0,4	partial CO dehydrogenase/acetyl-CoA synthase alpha subunit acsB
scal01606	23	3	248	0,4	putative phosphoketolase
scal02751	33	3	248	0,2	similar to iron sulfur
scal03505	60	3	248	0,1	squalene hopene cyclase
scal04330	12	3	248	0,8	conserved protein
scal01100	45	3	248	0,2	similar to 2-acylglycerophosphoethanolamine acyltransferase/acyl carrier protein synthetase
scal01027	6	3	248	2,2	strongly similar to 50S ribosomal protein L23
scal03024	12	3	248	0,8	rubredoxin-like superoxide reductase
scal01941	15	3	248	0,6	similar to chemotaxis protein CheY
scal04321	5	3	248	3,0	30S ribosomal protein S21
scal04151	45	3	248	0,2	strongly similar to ribonuclease E (RNase E)
scal01410	43	3	248	0,2	strongly similar to signal recognition particle protein
scal03646	37	3	248	0,2	serine hydroxymethyl transferase SHMT
scal03474	73	3	248	0,1	No hits found
scal03652	60	3	248	0,1	phenylalanyl-tRNA synthetase beta chain
scal00660	48	3	248	0,2	expressed NADH:ubiquinone oxidoreductase subunit nuoF
scal02389	37	3	248	0,2	WD40-like Beta Propeller
scal02861	74	3	248	0,1	formate dehydrogenase, alpha subunit
scal04191	27	3	248	0,3	hypothetical protein
scal01251	67	3	248	0,1	putative GAF sensor signal transduction histidine kinase
scal03759	94	3	248	0,1	fructose-bisphosphatase
scal01561	30	3	248	0,3	fructose-bisphosphate aldolase
scal00705	30	3	248	0,3	putative iron sulfur
scal02308	54	3	248	0,1	aconitate hydratase
scal00729	27	3	248	0,3	dihydropicolinate synthase
scal01017	12	3	248	0,8	30S ribosomal protein S8
scal00514	54	3	248	0,1	similar to Na(+)-translocating NADH-quinone reductase subunit F
scal02485	15	3	248	0,6	ATP-dependent endopeptidase Clp of CO dehydrogenase cluster
scal02808	46	3	248	0,2	transketolase
scal00367	38	3	248	0,2	strongly similar to pleiotrophic regulatory protein DegT
scal01935	55	3	248	0,1	similar to two component sensor histidine kinase
scal00454	50	3	248	0,1	expressed putative multiheme cytochrome c protein with 5 cxxch motives
scal02928	38	3	248	0,2	similar to carboxy-terminal processing protease
scal00194	39	3	248	0,2	argininosuccinate lyase argh
scal03235	24	3	248	0,3	3-oxoacyl acyl-carrier protein reductase

scal03076	15	3	248	0,6	putative rubrerythrin
scal03184	56	3	248	0,1	anthranilate synthase
scal00346	30	3	248	0,3	expressed threonine synthase thrC
scal00241	47	3	248	0,2	strongly similar to CTP synthase (UTP-ammonia ligase)
scal03529	28	3	248	0,3	similar to quinolinate synthetase A
scal02395	14	3	248	0,6	sur A peptidyl-prolyl cis-trans isomerase
scal02101	32	3	248	0,2	SAM radical nirJ like heme d synthesis protein
scal01247	21	3	248	0,4	conserved hypothetical cog2928 protein
scal02331	16	3	248	0,5	putative iron sulfur heterodisulfide reductase subunit
scal04145	29	3	248	0,3	succinyl-CoA synthetase, alpha subunit
scal03188	54	3	248	0,1	hypothetical protein
scal01552	27	3	248	0,3	putative molybdopterin containing oxidoreductase N terminus
scal01557	9	3	248	1,2	nuoG or FDH like molybdopterin containing oxidoreductase
scal03068	28	3	248	0,3	putative stress protein
scal01701	13	3	248	0,7	hydroxylamine oxidoreductase hao
scal03930	24	3	248	0,3	cytochrome c peroxidase
scal02795	10	3	248	2,2	chaperonin GroEL
scal02834	26	3	248	0,3	strongly similar to N-acetyl-gamma-glutamyl-phosphate reductase (NAGSA dehydrogenase)
scal02106	19	3	248	0,4	putative rieske 2Fe-2S protein of bc1 complex
orf07166	9	3	248	1,2	similar to acyl carrier protein
scal02956	13	3	248	0,7	similar to (3R)-hydroxymyristoyl acyl carrier protein dehydrase
scal03319	33	3	248	0,2	putative NAD-dependent epimerase/dehydratase
scal00239	24	3	248	0,3	expressed putative PpiC-type peptidyl-prolyl cis-trans isomerase
scal01385	18	3	248	0,5	protein of unknown function DUF500
scal02056	16	3	248	0,5	Rieske iron sulfur protein
scal00209	23	3	248	0,4	hypothetical protein
scal03921	34	3	248	0,2	putative TPR repeat protein
scal01480	51	3	248	0,1	hypothetical FAD oxidoreductase protein
scal02633	18	3	248	0,5	putative multiheme protein with 6 cxxch motives and 1 cxxxch motive of hydrazine synthase cluster
scal02283	10	3	248	1,0	50S ribosomal protein L11
orf05273	11	3	248	0,9	hypothetical protein
scal01028	17	3	248	0,5	50S ribosomal protein L4
scal00342	16	3	248	0,5	conserved hypothetical protein
scal02597	14	3	248	0,6	GreA/GreB family elongation factor
scal03846	8	3	248	1,4	preprotein translocase, YajC subunit
scal01558	17	3	248	0,5	putative carbon monoxide dehydrogenase, iron sulfur subunit CooF
orf05822	9	3	248	1,2	conserved hypothetical protein
scal02170	15	3	248	0,6	single-stranded DNA-binding protein
scal00931	12	3	248	0,8	similar to nitrogen regulatory protein P-II family proteins
scal02374	31	3	248	0,2	similar to ispE 4-diphosphate cytidyl 2-C methyl D erythritol kinase
scal01542	19	3	248	0,4	putative rubrerythrin/rubredoxin protein
scal02243	13	3	248	0,7	30S ribosomal protein S9
scal00521	77	3	248	0,1	expressed putative Zn dependent protease
scal00993	31	3	248	0,2	RecA protein (recombinase A)
scal01169	13	3	248	0,7	unknown anammox protein
scal03225	12	3	248	0,8	No hits found
scal02245	14	3	248	0,6	unknown protein with 1 cxxch
scal03839	21	3	248	0,4	3-deoxy-D-arabino-heptulosonate 7-phosphate synthase
scal02665	40	2	341	0,2	putative bifunctional GTP cyclohydrolase II / 3,4-dihydroxy-2-butanone 4-phosphate synthase
scal00777	70	2	341	0,1	hypothetical protein

scal00308	42	2	341	0,2	conserved hypothetical protein
scal03069	19	2	341	0,4	universal stress protein uspA
scal00632	11	2	341	0,9	bifunctional phosphoribosyl-AMP cyclohydrolase/ phosphoribosyl-ATP pyrophosphatase hisI
scal00199	17	2	341	0,5	expressed delta subunit of F0F1 ATP synthase
scal01128	32	2	341	0,2	putative ATP:guanido phosphotransferase / arginine kinase
scal02109	21	2	341	0,4	putative multiheme cytochrome c protein with 6 cxxch motives
scal02424	33	2	341	0,2	glutamyl-tRNA(Gln) amidotransferase subunit A
scal00953	9	2	341	1,2	cytochrome c-552 ks_3358
scal00688	36	2	341	0,2	putative cytochrome bd quinol oxidase (cydA) of nar cluster
scal03336	34	2	341	0,2	Transketolase-like
scal02806	26	2	341	0,2	conserved hypothetical protein
scal01234	17	2	341	0,3	CO dehydrogenase/acetyl-CoA synthase alpha subunit (acsB)partial C-terminus
scal03041	8	2	341	0,6	PKD
scal02909	45	2	341	0,1	similar to isopropylmalate synthase
scal02059	28	2	341	0,2	asrC anaerobic sulfite reductase subunit C
scal04044	20	2	341	0,3	chromosome partitioning protein ParA
scal03974	13	2	341	0,4	hydroxylamine oxidoreductase hao
scal04096	15	2	341	0,4	conserved hypothetical protein
orf01625	6	2	341	1,2	unknown protein
orf05337	6	2	341	1,2	conserved hypothetical protein
scal00361	25	2	341	0,2	strongly similar to inorganic polyphosphate/ATP-NAD kinase
scal01131	14	2	341	0,4	conserved hypothetical protein
scal04033	12	2	341	0,5	putative ABC type transport protein, permease component
scal00416	11	2	341	0,5	expressed formate/nitrite transporter family protein focA
scal01001	15	2	341	0,4	strongly similar to riboflavin synthase of Aquifex aeolicus
scal01947	12	2	341	0,5	putative response regulator
scal01315	25	2	341	0,2	tryptophan synthase alpha chain
orf02276	5	2	341	1,5	No hits found
scal01382	20	2	341	0,3	No hits found
scal03601	36	2	341	0,1	strongly similar to aspartokinase
scal04328	5	2	341	1,5	partial cold shock protein CspB
scal02648	25	2	341	0,2	V-type ATPase subunit E
scal02754	38	2	341	0,1	strongly similar to RNA polymerase subunit sigma-54
scal00452	33	2	341	0,1	cytochrome c peroxidase
scal02104	29	2	341	0,2	hypothetical cgg1413 HEAT repeat protein
scal00366	90	2	341	0,1	unknown protein
scal01622	57	2	341	0,1	similar to membrane associated lipoprotein involved in thiamine biosynthesis apbE
scal02631	35	2	341	0,1	biotin carboxylase (A subunit of acetyl-CoA carboxylase)
scal02403	41	2	341	0,1	kusta0023 IlvD;strongly similar to dihydroxyacid dehydratase;amino acid metabolism;4.2.1.9
scal00229	21	2	341	0,2	putative solvent tolerance protein
scal02166	28	2	341	0,2	phosphoribosylpyrophosphate synthetase
scal02674	55	2	341	0,1	putative ABC transporter ATP-binding protein
scal00246	61	2	341	0,1	expressed type III glutamate-ammonia ligase
scal01161	71	2	341	0,1	oxygen sensitive ribonucleoside triphosphate reductase
scal02239	50	2	341	0,1	putative Zn-dependent protease
scal02899	31	2	341	0,2	putative acetylglutamate kinase
scal02287	22	2	341	0,2	conserved hypothetical protein
scal00212	53	2	341	0,1	strongly similar to ATP-dependent zinc-metalloprotease ftsH involved in cell division
scal03793	37	2	341	0,1	strongly similar to chorismate mutase / prephenate dehydratase
scal02252	19	2	341	0,3	putative protein kinase (DUF1566)

scal03031	11	2	341	0,5	putative Rubrerythrin
scal03755	10	2	341	0,6	conserved hypothetical protein
scal00962	22	2	341	0,2	hypothetical protein VvadDRAFT_0226
scal03029	32	2	341	0,2	FAD-dependent pyridine nucleotide-disulphide oxidoreductase
scal02026	68	2	341	0,1	similar to glycine-tRNA ligase (beta-chain)
scal04172	18	2	341	0,3	expressed conserved hypothetical protein mostly found in microaerophilic metal-metabolizing and/or nitrogen-fixing microbes
scal03936	54	2	341	0,1	putative GlcNAc transferase
scal02488	24	2	341	0,2	putative nickel insertase (acsF) of CODH/ACS complex
scal02614	61	2	341	0,1	strongly similar to glucosamine-fructose-6-phosphate aminotransferase
scal00630	51	2	341	0,1	expressed resB-like type II cytochrome maturation protein
scal02399	37	2	341	0,1	similar to dihydroorotase
scal02855	33	2	341	0,1	transcription elongation protein NusA
scal02463	31	2	341	0,2	sulfate adenylyltransferase, small subunit
scal00824	37	2	341	0,1	Inositol-3-phosphate synthase
scal04160	76	2	341	0,1	isocitrate dehydrogenase, NADP-dependent
scal02527	49	2	341	0,1	putative NAD(P) oxidoreductase, FAD-containing subunit
scal02621	27	2	341	0,2	5'-methylthioadenosine phosphorylase
scal02299	10	2	341	0,6	universal stress protein uspA
scal01243	38	2	341	0,1	2-phosphoglycerate dehydratase, enolase
scal01641	15	2	341	0,4	similar to to proton-translocating NADH dehydrogenase I, 24 kDa subunit (NuoE)
scal00574	21	2	341	0,2	phage shock protein A, PspA
scal02800	21	2	341	0,2	putative molecular chaperone protein grpE
scal02840	22	2	341	0,2	expressed conserved hypothetical protein, MarR family
scal00011	22	2	341	0,2	expressed putative dinB protein
scal00822	32	2	341	0,2	putative cycloartenol synthase-like protein
scal02095	21	2	341	0,2	flagellar motor protein motB
scal02237	30	2	341	0,2	conserved hypothetical protein of nirHD gene cluster involved in heme d1 biosynthesis
scal03792	26	2	341	0,2	triose-phosphate isomerase
scal04118	51	2	341	0,1	prolyl-tRNA synthetase
scal02265	33	2	341	0,1	phosphoribosylaminoimidazole-succinocarboxamide (SAICAR) synthase
scal02458	20	2	341	0,3	similar to 1-acyl-sn-glycerol-3-phosphate acyltransferase (1-AGPacyltransferase) (1-AGPAT) (lysophosphatidic acid acyltransferase) (LPAAT).
scal00558	10	2	341	0,6	expressed GlnK nitrogen regulatory protein P-II
scal02849	5	2	341	1,5	conserved hypothetical protein
scal01020	10	2	341	0,6	50S ribosomal protein L14
scal02086	30	2	341	0,2	strongly similar to acetyl-CoA carboxylase carboxyltransferase alpha chain
scal03333	28	2	341	0,2	pyridoxal phosphate-dependent enzyme apparently involved in regulation of cell wall biogenesis
scal00032	26	2	341	0,2	expressed iron-sulfur protein NifS
scal00932	13	2	341	0,4	hypothetical protein MED92_02279
scal02415	40	2	341	0,1	strongly similar to GTP-binding protein TypA
scal01227	25	2	341	0,2	putative TPR repeat containing methyltransferase
scal02583	42	2	341	0,1	conserved hypothetical protein
scal00945	27	2	341	0,2	dihydrodipicolinate reductase
scal00428	29	2	341	0,2	putative cell shape-determining protein MreB
scal03789	29	2	341	0,2	putative stress protein
scal00192	18	2	341	0,3	expressed putative thiJ thiazole monophosphate biosynthesis protein
scal00281	22	2	341	0,2	putative plastocyanine containing petE protein
scal02332	31	2	341	0,2	putative heterodisulfide reductase subunit B
scal00171	23	2	341	0,2	expressed conserved hypothetical protein
scal02541	30	2	341	0,2	malate dehydrogenase
orf00338	8	2	341	0,8	F0F1 ATP synthase subunit epsilon

scal02401	35	2	341	0,1	conserved hypothetical protein
scal03216	50	2	341	0,1	hypothetical protein
scal02989	23	2	341	0,2	conserved hypothetical protein
scal04138	8	2	341	0,8	No hits found
scal02884	27	2	341	0,2	unknown protein
scal00238	23	2	341	0,2	hypothetical protein
scal01644	21	2	341	0,2	stongly similar to NADH dehydrogenase subunit
orf02623	2	2	341	9,0	similar to 30S ribosomal protein RpsT
scal02285	9	2	341	0,7	similar to preprotein translocase SecE subunit
scal01479	22	2	341	0,2	thioredoxin reductase
scal00532	35	2	341	0,1	hypothetical protein MB2181_02330
scal00726	25	2	341	0,2	putative molybdopterin synthesis protein MoeB and thiamin biosynthesis protein ThiF
scal02897	25	2	341	0,2	ornithine carbamoyltransferase
scal00714	11	2	341	0,5	conserved hypothetical protein
scal00146	41	2	341	0,1	aminotransferase class III gabT
scal01395	67	2	341	0,1	similar to Uncharacterized conserved protein
scal01216	38	2	341	0,1	strongly similar to methionyl-tRNA synthetase
scal01003	13	2	341	0,4	conserved hypothetical protein
scal03691	18	2	341	0,3	putative norVW-like flavodoxin protein
scal00211	19	2	341	0,3	hypothetical protein
scal00200	9	2	341	0,7	expressed B subunit of F0F1 ATP synthase
scal02276	8	2	341	2,7	30S ribosomal protein S7
scal04150	9	2	341	0,7	50S ribosomal protein L21
scal04163	7	2	341	0,9	putative periplasmic lipoprotein
scal02334	17	2	341	0,3	putative Methyl-viologen-reducing hydrogenase, delta subunit, heterodisulfide reductase subunit D
scal04192	14	2	341	0,4	adenine phosphoribosyltransferase
scal00891	32	2	341	0,2	sorbitol dehydrogenase
scal01648	30	2	341	0,2	strongly similar to NAD(P)H:quinone oxidoreductase chain 5
scal01697	9	2	341	0,7	conserved hypothetical protein
scal00721	31	2	341	0,2	conserved hypothetical protein
scal02130	13	2	341	0,4	putative iron sulfur cofactor assembly protein
scal03676	9	2	341	0,7	RNA-binding region RNP-1 (RNA recognition motif)
scal02108	20	2	341	0,3	putative cytochrome b protein of bc1 complex
scal00175	16	2	341	0,3	peptidyl-prolyl cis-trans isomerase A
scal04309	6	2	341	1,2	30S ribosomal protein S19
scal04024	32	2	341	0,2	check annotation in kust; putative metal dependent phosphohydrolase
scal03163	36	2	341	0,1	No hits found
scal02854	17	2	341	0,3	anthranilate synthase component II pabA
scal02277	7	2	341	0,9	30S ribosomal protein S12
scal02181	17	2	341	0,3	ribosome recycling factor
scal02802	21	2	341	0,2	unknown protein
scal02328	25	2	341	0,2	conserved hypothetical protein
scal01004	14	2	341	0,4	50S ribosomal protein L17
scal01962	24	2	341	0,2	similar to Flagellar Rotor Protein FliG
scal02368	50	2	341	0,1	similar to 3-dehydroquinate dehydratase / shikimate 5-dehydrogenase
scal01833	66	2	341	0,0	unknown protein
scal04283	55	1	489	0,1	strongly similar to transcription initiation factor sigma RpoD
scal03082	14	1	489	0,4	thioredoxin
scal03542	26	1	489	0,2	strongly similar to glycine tRNA synthetase, alpha subunit
scal03754	7	1	489	0,9	unknown protein

scal01084	4	1	489	2,2	unknown protein
scal04315	3	1	489	3,6	acyl carrier protein AcpP
scal01583	10	1	489	0,5	citrate synthase
scal00787	25	1	489	0,1	hypothetical protein
scal03354	41	1	489	0,1	H(+)-transporting ATP synthase, subunit alpha
scal01041	77	1	489	0,0	Acriflavin resistance protein
scal00422	104	1	489	0,0	putative TPR repeat protein
scal03696	58	1	489	0,0	hypothetical protein with PilF (COG3063) domain
scal02071	32	1	489	0,1	strongly similar to thiamin biosynthesis protein ThiC
scal04259	7	1	489	0,4	hypothetical protein
scal01847	38	1	489	0,1	kusta0010 MltA;similar to membrane-bound lytic murein transglycosylase A
scal02682	5	1	489	0,6	putative cold shock protein CspB
scal00776	31	1	489	0,1	alpha/beta hydrolase fold
scal02714	5	1	489	0,6	unknown protein
scal01999	28	1	489	0,1	Methyltransferase type 12
scal00590	11	1	489	0,2	GlnK nitrogen regulatory protein P-II
scal04237	44	1	489	0,1	conserved hypothetical protein
scal00453	10	1	489	0,3	putative rubrerythrin/rubredoxin protein
scal00757	14	1	489	0,2	transcription elongation factor greA
scal01719	36	1	489	0,1	similar to glycosyl transferase protein cotS
scal00661	9	1	489	0,3	expressed putative nuoE of NADH:ubiquinone oxidoreductase
scal01572	12	1	489	0,2	hypothetical protein Dace_0111
scal04043	11	1	489	0,2	strongly similar to hydroxymyristoyl acyl carrier protein dehydratase
scal02336	34	1	489	0,1	putative 4Fe-4S ferredoxin hydrogenase beta subunit
scal00534	12	1	489	0,2	expressed putative ribose-5-phosphate isomerase rpiB
scal03720	31	1	489	0,1	general secretion pathway protein A
scal01180	16	1	489	0,2	hypothetical morn protein
scal03040	7	1	489	0,4	conserved hypothetical protein
scal02407	31	1	489	0,1	3-oxoacyl-[acyl-carrier-protein] synthase II (KASII)
scal01073	7	1	489	0,4	No hits found
scal00608	49	1	489	0,0	MORN repeat protein
scal02632	2	1	489	0,9	hypothetical (triheme) protein of hydrazine synthase cluster with 3 cxxch and 1 cxch mottive
scal00567	6	1	489	0,5	RNA-binding region RNP-1 (RNA recognition motif)
scal01182	33	1	489	0,1	GTP binding protein
scal02295	74	1	489	0,0	similar to sensor histidine kinase/response regulator protein NtrY
scal04203	48	1	489	0,0	putative histidine sensor kinase
scal01401	33	1	489	0,1	strongly similar to 3-phosphoshikimate 1-carboxyvinyltransferase
scal00375	27	1	489	0,1	pdb|1NA0|A Chain A, Design Of Stable Alpha-Helical Arrays From An Idealized Tpr Motif
scal03536	59	1	489	0,0	similar to ABC transporter MsbA
scal01335	53	1	489	0,0	copper-translocating P-type ATPase
scal00324	27	1	489	0,1	expressed malonyl-CoA-[acyl-carrier-protein] transacylase
scal01595	26	1	489	0,1	geranylgeranyl hydrogenase BchP, putative
scal03182	41	1	489	0,1	similar to 7,8-diaminopelargonic acid synthase
scal04274	36	1	489	0,1	D-xylulose 5-phosphate/D-fructose 6-phosphate phosphoketolase family protein
orf07565	71	1	489	0,0	putative phosphoketolase
scal02288	53	1	489	0,0	putative multiheme cytochrome c protein with 8 heme / cxxch motives
scal02986	31	1	489	0,1	cell surface protein
scal02616	38	1	489	0,1	putative threonine synthase thrC
scal03909	89	1	489	0,0	putative type I restriction protein
scal01875	35	1	489	0,1	No hits found

scal03741	56	1	489	0,0	glycogen synthase
scal00139	36	1	489	0,1	Homoserine dehydrogenase
scal02100	35	1	489	0,1	putative nirJ heme d1 biosynthesis protein
scal01344	75	1	489	0,0	putative multiheme protein with 14 cxxch motives
scal03008	15	1	489	0,2	unknown protein
scal04216	66	1	489	0,0	DNA polymerase B region
scal02936	16	1	489	0,2	putative 4Fe-4S ferredoxin, iron-sulfur binding
scal01232	23	1	489	0,1	similar to kinesin light chain KLC
scal02269	43	1	489	0,1	strongly similar to glutamine phosphoribosylpyrophosphate amidotransferase
scal02634	20	1	489	0,1	putative cytochrome b protein of hydrazine synthase cluster
scal00228	54	1	489	0,0	transport protein, putative
scal03815	75	1	489	0,0	putative TPR+C255 repeat protein
scal01290	46	1	489	0,1	similar to capsular polysaccharide synthesis protein
scal01684	25	1	489	0,1	unknown protein
scal03481	32	1	489	0,1	Glutathione synthase
scal02021	48	1	489	0,0	UDP-glucose 6-dehydrogenase
scal04042	35	1	489	0,1	beta-ketoacyl acyl carrier protein synthase II
scal03678	95	1	489	0,0	type I DNA restriction-modification system
scal03353	30	1	489	0,1	similar to ATPG gene encoding subunit gamma of ATP synthase
scal01102	47	1	489	0,1	cysteinyl-tRNA synthetase
scal01153	57	1	489	0,0	similar to glycerophosphodiester phosphodiesterase
scal02918	59	1	489	0,0	Secretion protein HlyD like protein
scal02626	16	1	489	0,2	hypothetical protein
scal02388	53	1	489	0,0	putative M28 peptidase
scal00557	73	1	489	0,0	putative thioredoxin
scal01019	9	1	489	0,3	50S ribosomal protein L24
scal00203	32	1	489	0,1	expressed glutamate-1-semialdehyde aminomutase
scal03316	45	1	489	0,1	FAD-dependent pyridine nucleotide-disulphide oxidoreductase
scal03737	73	1	489	0,0	glycoside hydrolase, family 57
scal03480	37	1	489	0,1	Glutathione synthase
scal01054	24	1	489	0,1	putative zinc finger protein
scal00925	13	1	489	0,2	No hits found
scal00599	28	1	489	0,1	expressed putative phosphoserine phosphatase
scal03577	17	1	489	0,1	putative TonB-dependent receptor protein
scal02852	54	1	489	0,0	glutamyl-tRNA(Gln) amidotransferase, B subunit
scal01007	7	1	489	0,4	30S ribosomal protein S11
scal01130	39	1	489	0,1	conserved hypothetical protein
scal00537	15	1	489	0,2	expressed conserved hypothetical COG2001 protein
scal00562	25	1	489	0,1	expressed putative glycine cleavage system P-protein
scal02526	20	1	489	0,1	putative cytochrome b6
scal01378	39	1	489	0,1	hypothetical protein
scal02893	52	1	489	0,0	conserved hypothetical GTP-binding protein
scal00722	25	1	489	0,1	putative thymidylate synthase ThyX
scal02627	31	1	489	0,1	hypothetical protein
scal03335	26	1	489	0,1	Pyruvate dehydrogenase (lipoamide)
scal00856	13	1	489	0,2	hypothetical protein PM8797T_31598
scal02942	25	1	489	0,1	peptidase M23B
scal01058	34	1	489	0,1	Alcohol dehydrogenase
scal02418	40	1	489	0,1	Na+-transporting methylmalonyl-CoA/oxaloacetate decarboxylase, beta subunit
scal04267	23	1	489	0,1	hypothetical protein Gura_2669

scal02392	13	1	489	0,2	strongly similar to aspartate 1-decarboxylase precursor (aspartate alpha-decarboxylase).
scal04105	47	1	489	0,1	expressed conserved hypothetical COG5316 protein
scal01833	57	1	489	0,0	unknown protein
scal01649	28	1	489	0,1	similar to NADH:ubiquinone oxidoreductase subunit M
scal00734	17	1	489	0,1	hypothetical protein
scal00005	75	1	489	0,0	Methyltransferase type 11
scal01313	38	1	489	0,1	partial hypothetical bc fusion protein
scal01911	30	1	489	0,1	oxidoreductase, Gfo/Idh/MocA family/transferase hexapeptide repeat protein
scal02762	32	1	489	0,1	Porphyromonas-type peptidyl-arginine deiminase
orf07369	9	1	489	0,3	No hits found
scal03485	9	1	489	0,3	putative molybdopterin-containing NuoG subunit of NADH:ubiquinone oxidoreductase
scal00971	17	1	489	0,1	Hemerythrin HHE cation binding domain protein
scal04329	4	1	489	0,8	putative tatA/E protein
scal00345	9	1	489	0,3	similar to molybdopterin synthase subunit 1
scal01282	19	1	489	0,1	No hits found
scal04018	16	1	489	0,2	conserved hypothetical protein
scal03360	51	1	489	0,0	ATP synthase subunit B
scal02793	10	1	489	0,3	hypothetical protein CAB357
scal00586	26	1	489	0,1	putative MazG like pyrophosphatase with methyltransferase domain
scal02244	24	1	489	0,1	UDP-glucose 4-epimerase
scal02799	30	1	489	0,1	strongly similar to heat shock protein DnaJ
scal03919	18	1	489	0,1	hypothetical duf1318 membrane protein
scal00763	12	1	489	0,2	hypothetical cytosolic protein
scal04197	24	1	489	0,1	hypothetical protein
scal03738	45	1	489	0,1	strongly similar to 4-alpha-glucanotransferase
orf00928	4	1	489	0,8	putative Sec-independent protein translocase component
scal02636	18	1	489	0,1	unknown protein part of the hydrazine synthase cluster
scal00347	34	1	489	0,1	conserved hypothetical protein
scal02078	31	1	489	0,1	strongly similar to aspartate transcarbamoylase catalytic chain
scal03304	18	1	489	0,1	DNA polymerase III beta subunit
scal03334	10	1	489	0,3	Sugar transferase involved in lipopolysaccharide synthesis-like
scal00826	33	1	489	0,1	O-methyltransferase, family 2:Generic methyltransferase
scal02386	9	1	489	0,3	hypothetical protein DoleDRAFT_1523
orf06741	33	1	489	0,1	similar to TonB-dependent receptor protein
scal04137	15	1	489	0,2	putative TonB-dependent receptor protein
scal04117	18	1	489	0,1	putative cytochrome c protein with 1 cxxch motive
scal01314	15	1	489	0,2	rieske iron sulfur (2Fe-2S) protein of the bc1 complex
scal00711	12	1	489	0,2	conserved hypothetical protein containing Rhodanese Homology Domain (RHOD)
scal00653	15	1	489	0,2	hypothetical protein
scal03506	56	1	489	0,0	type III restriction enzyme, res subunit
scal01645	16	1	489	0,2	conserved hypothetical protein of nirHD gene cluster involved in heme d1 biosynthesis
scal01743	34	1	489	0,1	methyl-accepting chemotaxis sensory transducer
scal04107	26	1	489	0,1	ExsB transcriptional regulator
scal02248	22	1	489	0,1	beta-lactamase-like protein
scal03865	37	1	489	0,1	delta-aminolevulinate dehydratase (porphobilinogen synthase)
scal01403	18	1	489	0,1	conserved hypothetical protein
scal01281	19	1	489	0,1	No hits found
scal02370	16	1	489	0,2	ATP-dependent endopeptidase Clp
scal01011	21	1	489	0,1	adenylate kinase
scal01008	26	1	489	0,1	conserved hypothetical protein

scal03205	40	1	489	0,1	strongly similar to histidinol dehydrogenase
scal02414	23	1	489	0,1	hypothetical protein NE1275
scal00031	80	1	489	0,0	strongly similar to ATP-dependent DNA helicase
scal00339	39	1	489	0,1	hemA glutamyl-tRNA reductase
scal04183	22	1	489	0,1	conserved hypothetical protein
scal00104	27	1	489	0,1	putative hydrogenase gamma subunit
scal02929	26	1	489	0,1	similar to riboflavin biosynthesis protein RibF
scal00348	13	1	489	0,2	conserved hypothetical protein
scal00261	87	1	489	0,0	conserved putative chloride channel
scal00706	32	1	489	0,1	acrA cation efflux transport protein
scal02376	19	1	489	0,4	acetohydroxy acid synthase IilvB
scal02058	18	1	489	0,1	conserved hypothetical COG2210 protein
scal00598	28	1	489	0,1	putative phosphoserine phosphatase
scal03217	28	1	489	0,1	unknown protein
scal03632	23	1	489	0,1	phosphoenolpyruvate phosphomutase
scal02121	22	1	489	0,1	probable transcriptional regulator protein
scal03476	30	1	489	0,1	trkA domain protein
scal02160	22	1	489	0,1	putative sensor protein
scal03342	41	1	489	0,1	hypothetical protein PH1879
scal03229	42	1	489	0,1	hypothetical protein
scal02350	25	1	489	0,1	conserved hypothetical protein
scal01466	31	1	489	0,1	Fe-S iron sulfur cluster assembly NifU-like protein
scal00555	28	1	489	0,1	adenylylsulfate reductase, thioredoxin dependent
orf02560	7	1	489	0,4	Uncharacterized conserved protein UCP037465, zinc finger protein, AF1427
orf05842	7	1	489	0,4	Uncharacterized conserved protein UCP037465, zinc finger protein, AF1427
orf07388	7	1	489	0,4	Uncharacterized conserved protein UCP037465, zinc finger protein, AF1427
orf04962	6	1	489	0,5	conserved hypothetical protein
orf07405	16	1	489	0,2	No hits found
scal00629	20	1	489	0,1	resC/ccsA type II cytochrome c biogenesis protein
scal04031	6	1	489	0,5	No hits found
scal03194	21	1	489	0,1	strongly similar to acetyl-CoA carboxylase, beta subunit
scal02422	14	1	489	0,2	ferric uptake regulator protein
orf02370	5	1	489	0,6	strongly similar to 30S ribosomal protein S16
scal04155	5	1	489	0,6	conserved hypothetical protein
scal01563	26	1	489	0,1	strongly similar to fructose-1,6-bisphosphatase
scal03361	13	1	489	0,2	hypothetical protein Ping_2356
scal03571	28	1	489	0,1	nucleic acid binding protein
scal04306	5	1	489	0,6	undefined product
scal01033	20	1	489	0,1	hypothetical protein
scal01140	41	1	489	0,1	Undecaprenyl-phosphate galactose phosphotransferase
orf05272	6	1	489	0,5	predicted protein
scal02090	10	1	489	0,3	strongly similar to to proton-translocating NADH dehydrogenase I, 24 kDa subunit (NuoE)
scal03070	17	1	489	0,1	universal stress protein uspA
scal02111	7	1	489	0,4	putative cell wall associated protein
orf02355	6	1	489	0,5	cold-shock DNA-binding domain protein
scal03028	14	1	489	0,2	secreted protein
scal02712	21	1	489	0,1	unknown protein
scal00639	27	1	489	0,1	expressed 3-hydroxybutyryl-CoA dehydrogenase
scal01281	22	1	489	0,1	No hits found
scal02917	31	1	489	0,1	hypothetical protein

scal04297	29	1	489	0,1	conserved hypothetical protein of aldo-/ketoreductase family
scal03813	24	1	489	0,1	TPR repeat
scal00846	33	1	489	0,1	conserved hypothetical protein
scal01010	25	1	489	0,1	methionine aminopeptidase
scal02255	10	1	489	0,3	diaminohydroxyphosphoribosylaminopyrimidine deaminase / 5-amino-6-(5-phosphoribosylamino)uracil reductase
scal00365	17	1	489	0,1	strongly similar to phosphopantetheine adenylyltransferase (PPAT)
scal02706	16	1	489	0,2	hypothetical protein ObacDRAFT_3297
scal03460	16	1	489	0,2	hypothetical protein ObacDRAFT_3297
scal03765	1	1	489	9,0	No hits found
scal03015	17	1	489	0,1	lactoylglutathione lyase
scal02591	7	1	489	0,4	conserved hypothetical protein
scal01229	42	1	489	0,1	ferredoxin-dependent glutamate synthase
scal03675	8	1	489	0,3	No hits found
scal00703	30	1	489	0,1	conserved hypothetical COG1355 protein
scal00535	16	1	489	0,2	similar to YrdC protein
scal04217	15	1	489	0,2	No hits found
scal00669	4	1	489	0,8	similar to small heat shock protein
scal01293	32	1	489	0,1	putative tetraheme c554 cytochrome protein
