# Supplementary material for: The metagenome of the marine anammox bacterium ‘Candidatus Scalindua profunda’ illustrates the versatility of this globally important nitrogen cycle bacterium
Source: Environ Microbiol. 2013 May;15(5):1275–89. doi: 10.1111/j.1462-2920.2012.02774.x (PMC3655542; doi:10.1111/j.1462-2920.2012.02774.x)
Supplement: Supplementary file 13 [file emi0015-1275-SD13.pdf]

Table supplement 4 Expressed Scalindua genes not found in Kuenenia

**Scal**	**BesthitSpecies**	**possible function**	rel cov transcriptome	empai proteome	**AnnotScal**
scal00980	Marinobacter sp. ELB17	attachment	0,30	0,00	cyclic beta 1-2 glucan synthetase 1155965:1164586 forward MW:323132
scal01666	Desulfuromonas acetoxidans	attachment	0,27	0,00	Pilus assembly protein, PilO [Desulfuromonas acetoxidans DSM 684] gb|EAT16759.1| Pilus assembly protein, PilO [Desulfuromonas acetoxidans DSM 684] 1977071:1977697 forward MW:23494
orf06996	Thioalkalivibrio sp. HL-EbGR7	attachment	0,25	0,00	PilT domain-containing protein [Thioalkalivibrio sp. HL-EbGR7] gb|ACL73514.1| PilT domain-containing protein [Thioalkalivibrio sp. HL-EbGR7] 4737206:4737583 reverse MW:13821
scal03943(	Geobacter uraniumreducens	attachment	0,16	0,00	PilT protein domain protein [Geobacter uraniumreducens Rf4] gb|ABQ25605.1| PilT protein domain protein [Geobacter uraniumreducens Rf4] 4737206:4737586 reverse MW:13935
scal03997	Geobacter sp. FRC-32	attachment	0,11	0,00	PilT like protein 4798785:4799150 forward MW:13284
scal02317	Thermoanaerobacter tengcon	attachment	0,05	0,00	cellulose biosynthesis protein [Thermoanaerobacter tengcongensis MB4] gb|AAM23573.1| cellulose biosynthesis protein [Thermoanaerobacter tengcongensis MB4] 2818774:2819865 forward MW:43406
orf01913	Burkholderia phytofirmans Ps	attachment	0,00	0,00	PilT protein domain protein [Burkholderia phytofirmans PsJN] gb|ACD18756.1| PilT protein domain protein [Burkholderia phytofirmans PsJN] 1349849:1350118 reverse MW:10186
scal00761	Cyanothece sp. CCY0110	attachment	0,00	0,00	PilT protein-like [Cyanothece sp. CCY0110] gb|EAZ90815.1| PilT protein-like [Cyanothece sp. CCY0110] 881757:882152 forward MW:14876
scal01342	Syntrophobacter fumaroxidan	attachment	0,00	0,00	PilT protein domain protein [Syntrophobacter fumaroxidans MPOB] gb|ABK17098.1| PilT protein domain protein [Syntrophobacter fumaroxidans MPOB] 1601849:1602268 forward MW:15796
scal01849	Chlorobium limicola DSM 245	attachment	0,00	0,00	PilT protein, N-terminal [Chlorobium limicola DSM 245] gb|EAM42736.1| PilT protein, N-terminal [Chlorobium limicola DSM 245] 2253652:2254056 forward MW:15460
scal03608(	Pelobacter carbinolicus DSM	attachment	0,00	0,00	pilus assembly protein, PilO-like [Pelobacter carbinolicus DSM 2380] gb|ABA89375.1| pilus assembly protein, PilO-like [Pelobacter carbinolicus DSM 2380] 4352714:4353244 reverse MW:20462
scal01206(	Pelobacter carbinolicus DSM	carbon dioxide use	0,27	0,00	carbonic anhydrase [Pelobacter carbinolicus DSM 2380] gb|ABA90174.1| carbonic anhydrase [Pelobacter carbinolicus DSM 2380] 1428801:1429562 reverse MW:27098
scal00245(	Nitrococcus mobilis Nb-231	carbon dioxide use	0,23	0,00	ribulose-bisphosphate carboxylase-like protein; rubisco-like protein 299637:301238 reverse MW:59712
scal03046	Beggiatoa sp. PS	carbon dioxide use	0,07	0,00	ribulose-bisphosphate carboxylase-like protein; rubisco-like protein 3687973:3688410 forward MW:16525
scal03389(	Chlorobium phaeobacteroides	carbon dioxide use	0,05	0,00	Carbonate dehydratase [Chlorobium phaeobacteroides BS1] gb|EAM63340.1| Carbonate dehydratase [Chlorobium phaeobacteroides BS1] 4093216:4093845 reverse MW:24016
scal00602(	Desulfotalea psychrophila LS	carbon dioxide use	0,00	0,00	putative beta class carbonic anhydrase 702199:702828 reverse MW:23780
scal03005(	Lyngbya sp. PCC 8106	carbon dioxide use	0,00	0,00	carbonic anhydrase precursor [Lyngbya sp. PCC 8106] gb|EAW37375.1| carbonic anhydrase precursor [Lyngbya sp. PCC 8106] 3632594:3632911 reverse MW:12642
scal02421(	Flavobacteria bacterium BBFI	cytochrome c biosynthesis	0,01	0,00	putative resA type II cytochrome c biogenesis protein 2950596:2951363 reverse MW:28681
scal03348(	Planctomyces maris DSM 879	energy	2,42	0,00	hypothetical planctomyces protein with 1 cxxch motive 4039250:4039828 reverse MW:21566
scal01344(	Photobacterium profundum 3	energy	1,96	0,03	putative multiheme protein with 14 cxxch motives 1603206:1605623 reverse MW:89557
scal02335	Geobacter metallireducens G	energy	1,73	0,00	putative iron sulfur heterodisulfide reductase subunit 2842928:2843773 forward MW:32682
scal04293(	Planctomyces maris DSM 879	energy	1,26	0,83	putative cytochrome c protein C2 5127628:5128128 reverse MW:19056
scal02336	Pelodictyon luteolum DSM 27	energy	1,10	0,07	putative 4Fe-4S ferredoxin hydrogenase beta subunit 2843818:2844831 forward MW:38480
scal00185(	Shewanella sp. ANA-3	energy	1,03	0,00	putative catalase/peroxidase 225297:227675 reverse MW:87739
scal02331	Desulfotomaculum reducens	energy	0,75	0,54	putative iron sulfur heterodisulfide reductase subunit 2838202:2838756 forward MW:21177
scal00199(	Planctomyces maris DSM 879	energy	0,68	0,50	expressed delta subunit of F0F1 ATP synthase 245250:245801 reverse MW:20664
scal03511(	Leptospira borgpetersenii ser	energy	0,66	0,00	Carbamoyl transferase [Leptospira borgpetersenii serovar Hardjo-bovis L550] ref|YP_800489.1| Carbamoyl transferase [Leptospira borgpetersenii serovar Hardjo-bovis JB197] gb|ABJ78675.1| Carbamoyl
scal00457(	Alcanivorax borkumensis SK2	energy	0,62	0,00	putative iron sulfur protein 544822:545169 reverse MW:13244
scal00455(	Pseudomonas syringae pv. to	energy	0,41	0,00	putative glutathione peroxidase partial 543547:544104 reverse MW:20959
scal03660(	Thermosynechococcus elong	energy	0,39	0,00	NAD/NADP transhydrogenase beta subunit 4407110:4408477 reverse MW:48367
scal02515	Burkholderia phymatum STM	energy	0,32	0,00	Carbamoyltransferase [Burkholderia phymatum STM815] gb|EAU98770.1| Carbamoyltransferase [Burkholderia phymatum STM815] 3067648:3069342 forward MW:64118
scal03025(	Planctomyces maris DSM 879	energy	0,32	0,00	hypothetical planctomyces protein with 1 cxxch motive 3662301:3662888 reverse MW:22052
scal03661(	Crocosphaera watsonii WH 8	energy	0,30	0,00	NAD/NADP transhydrogenase alpha subunit 4408773:4409915 reverse MW:41440
scal03349(	Planctomyces maris DSM 879	energy	0,25	0,00	hypothetical planctomyces protein with 1 cxxch motive 4039882:4040442 reverse MW:21047
scal02913(	Geobacter bemidjiensis Bem	energy	0,23	0,00	putative cytochrome c protein with 1 cxxch motive 3526396:3527958 reverse MW:56523
scal01461	Syntrophus aciditrophicus SB	energy	0,18	0,00	putative cytochrome c3 hydrogenase subunit 1754312:1755358 forward MW:40324
orf07381	gamma proteobacterium NOF	energy	0,02	0,00	Na+/H+ antiporter [gamma proteobacterium NOR5-3] gb|EED34029.1| Na+/H+ antiporter [gamma proteobacterium NOR5-3] 4989105:4989359 reverse MW:9403
scal00148	Geobacter bemidjiensis Bem	energy	0,02	0,00	putative cytochrome b protein of putative nrf cluster 178241:178534 forward MW:11549
scal03550	Saccharophagus degradans 2	energy	0,02	0,00	NA(+)/H(+) antiporter 1 [Saccharophagus degradans 2-40] gb|ABD81022.1| Na+/H+ antiporter NhaA [Saccharophagus degradans 2-40] 4298101:4299486 forward MW:50462
scal00113	Roseovarius sp. 217	energy	0,01	0,00	putative type-2 Multicopper oxidase 136232:137947 forward MW:65567
scal03855	Crocosphaera watsonii WH 8	energy	0,01	0,00	putative carbamoyl transferase, NodU family protein 4630565:4631584 forward MW:38948
orf05399	Clostridium kluyveri DSM 555	energy	0,00	0,00	superoxide reductase-like protein [Clostridium kluyveri DSM 555] ref|YP_002473807.1| hypothetical protein CKR_3342 [Clostridium kluyveri NBRC 12016] gb|EDK35766.1| Rbo [Clostridium kluyveri DSM
scal00288(	Beggiatoa sp. PS	energy	0,00	0,00	hypothetical multiheme protein 355674:356594 reverse MW:35203
scal02782(	Marinobacter sp. ELB17	energy	0,00	0,00	probable carbamoyl transferase [Marinobacter sp. ELB17] gb|EAZ98675.1| probable carbamoyl transferase [Marinobacter sp. ELB17] 3382029:3383750 reverse MW:64762
scal03174(	Planctomyces maris DSM 879	energy	0,00	0,00	hypothetical planctomyces protein with 1 cxxch motive 3826162:3826548 reverse MW:14539
scal03385	Arthrobacter sp. FB24	energy	0,00	0,00	FAD dependent oxidoreductase [Arthrobacter sp. FB24] gb|ABK02649.1| FAD dependent oxidoreductase [Arthrobacter sp. FB24] 4087656:4087940 forward MW:10612
scal03854	Lyngbya sp. PCC 8106	energy	0,00	0,00	putative carbamoyl transferase, NodU family protein 4629768:4630595 forward MW:31534
scal04272(	Planctomyces maris DSM 879	energy	0,00	0,00	hypothetical planctomyces protein with 1 cxxch motive 5096517:5097098 reverse MW:22021
scal04277(	Syntrophus aciditrophicus SB	energy	0,00	0,00	adenylate cyclase [Syntrophus aciditrophicus SB] gb|ABC76860.1| adenylate cyclase [Syntrophus aciditrophicus SB] 5102893:5103378 reverse MW:18093
scal00821(	Geobacter bemidjiensis Bem	lipid synthesis	0,37	0,00	putative cycloartenol synthase-like protein 956853:957854 reverse MW:37624
scal00822(	Geobacter bemidjiensis Bem	lipid synthesis	0,21	0,15	putative cycloartenol synthase-like protein 957885:958964 reverse MW:40427
scal01730	Clostridium kluyveri DSM 555	more effective use organic acids	2,26	0,00	glutamate synthase 2050454:2055040 forward MW:169961
scal02037(	Mariprofundus ferrooxydans I	more effective use organic acids	1,51	0,00	putative carbohydrate-selective porin protein, OprB family 2482499:2483881 reverse MW:52046
scal00190(	Bacillus weihenstephanensis	more effective use organic acids	1,46	0,00	Phosphoglycerate/bisphosphoglycerate mutase [Bacillus weihenstephanensis KBAB4] gb|EAR73356.1| Phosphoglycerate/bisphosphoglycerate mutase [Bacillus weihenstephanensis KBAB4] 232336:233(
scal03759	Methanosarcina acetivorans (	more effective use organic acids	0,94	0,08	fructose-bisphosphatase [Methanosarcina acetivorans C2A] gb|AAM04573.1| fructose-bisphosphatase [Methanosarcina acetivorans C2A] 4526741:4529407 forward MW:102787
scal04141(	Methanosarcina acetivorans (	more effective use organic acids	0,94	0,22	phosphoglyceromutase [Methanosarcina acetivorans C2A] sp|Q8TMI6|GPMI1_METAC 2,3-bisphosphoglycerate-independent phosphoglycerate mutase 1 (Phosphoglyceromutase 1) (BPG-independent P
scal02807	Nitrococcus mobilis Nb-231	more effective use organic acids	0,91	0,00	putative glucokinase partial 3407480:3408505 forward MW:36868
scal01561	Anabaena variabilis ATCC 29	more effective use organic acids	0,82	0,26	fructose-bisphosphate aldolase 1864902:1865972 forward MW:38641
scal03987	Geobacter lovleyi SZ	more effective use organic acids	0,75	0,00	iron-containing alcohol dehydrogenase 4785497:4786648 forward MW:41581
scal00640(	Methanosarcina mazei Go1	more effective use organic acids	0,64	0,00	putative acetyl-CoA acetyltransferase; thiolase 748103:749254 reverse MW:41257
scal00638(	Hyperthermus butylicus DSM	more effective use organic acids	0,62	0,00	putative 3-hydroxy-3-methylglutaryl-coenzyme A reductase 745616:746977 reverse MW:50473
scal04160	Magnetococcus sp. MC-1	more effective use organic acids	0,59	0,06	isocitrate dehydrogenase, NADP-dependent 4976256:4978610 forward MW:86268
scal01229(	unidentified eubacterium SCE	more effective use organic acids	0,59	0,06	ferredoxin-dependent glutamate synthase [unidentified eubacterium SCB49] gb|EDM44916.1| ferredoxin-dependent glutamate synthase [unidentified eubacterium SCB49] 1453316:1454896 reverse MW:5
scal00639(	Thermoanaerobacter tengcon	more effective use organic acids	0,48	0,09	expressed 3-hydroxybutyryl-CoA dehydrogenase 746992:747861 reverse MW:31855
scal02429	Candidatus Desulfococcus ol	more effective use organic acids	0,37	0,00	fumarate reductase, cytochrome b subunit, putative [Candidatus Desulfococcus oleovorans Hxd3] gb|EAX54501.1| fumarate reductase, cytochrome b subunit, putative [Candidatus Desulfococcus oleovor
scal01583(	Methylococcus capsulatus str	more effective use organic acids	0,30	0,47	citrate synthase [Methylococcus capsulatus str. Bath] gb|AAU93059.1| citrate synthase [Methylococcus capsulatus str. Bath] 1886696:1888000 reverse MW:48952
scal00350(	Thermoanaerobacter ther	more effective use organic acids	0,27	0,00	acetate kinase [Thermoanaerobacterium thermosaccharolyticum] 417347:418564 reverse MW:45033
scal03477(	Parvibaculum lavamentivoran	more effective use organic acids	0,25	0,00	citrate synthase I [Parvibaculum lavamentivorans DS-1] gb|ABS64787.1| citrate synthase I [Parvibaculum lavamentivorans DS-1] 4200402:4201703 reverse MW:48992
scal01087(	Pseudomonas putida W619	more effective use organic acids	0,25	0,00	glucose-methanol-choline oxidoreductase [Pseudomonas putida W619] gb|EAX17365.1| glucose-methanol-choline oxidoreductase [Pseudomonas putida W619] 1271872:1272363 reverse MW:18144
scal00568	Bacillus sp. B14905	more effective use organic acids	0,07	0,00	putative glyoxalase protein 666010:666444 forward MW:16419
scal01588(	Mariprofundus ferrooxydans I	more effective use organic acids	0,07	0,00	putative carbohydrate-selective porin protein, OprB family 1892009:1893385 reverse MW:51871
scal04243	Gloeobacter violaceus PCC 7	more effective use organic acids	0,07	0,00	putative glucokinase partial 5067112:5068010 forward MW:32663

scal03801	Mariprofundus ferrooxydans P	more effective use organic acids	0,07	0,00	Carbohydrate-selective porin [Mariprofundus ferrooxydans PV-1] gb|EAU54090.1| Carbohydrate-selective porin [Mariprofundus ferrooxydans PV-1] 4568604:4569362 reverse MW:28058
scal01816	Lyngbya sp. PCC 8106	more effective use organic acids	0,00	0,00	glycerol-3-phosphate dehydrogenase partial 2147915:2148314 reverse MW:14750
scal02786	Parvibaculum lavamentivoran	more effective use organic acids	0,00	0,00	glycerol-3-phosphate dehydrogenase partial 3386809:3387408 reverse MW:22136
scal03670	Lyngbya sp. PCC 8106	more effective use organic acids	0,00	0,00	glycerol-3-phosphate dehydrogenase partial 4418300:4419376 reverse MW:40132
scal03972	Mariprofundus ferrooxydans P	more effective use organic acids	0,00	0,00	Carbohydrate-selective porin [Mariprofundus ferrooxydans PV-1] gb|EAU54090.1| Carbohydrate-selective porin [Mariprofundus ferrooxydans PV-1] 4766666:4767009 reverse MW:12825
scal00274	#N/A	nitric oxide stress	0,94	0,78	expressed flavoredoxin 342486:343073 forward MW:21323
scal02135	Geobacillus thermodenitrificai	nitric oxide stress	0,68	0,00	putative qnor nitric oxide reductase 2600177:2602555 forward MW:88780
scal00292	#N/A	nitric oxide stress	0,25	0,00	putative nitric-oxide reductase subunit B partial 357959:358498 reverse MW:19131
scal00931	Desulfotalea psychrophila LS	regulation N metabolism	1,37	0,78	similar to nitrogen regulatory protein P-II family proteins [Desulfotalea psychrophila LSv54] emb|CAG35078.1| related to nitrogen regulatory protein P-II family proteins [Desulfotalea psychrophila LSv54] 1(
scal02588	Agrobacterium tumefaciens st	transport	8,42	0,00	putative copper-transporting P-type ATPase 3151996:3152328 forward MW:11744
scal02105	Synechocystis sp. PCC 6803	transport	2,51	0,00	putative copper binding protein of the plastocyanin/azurin family 2561363:2561788 reverse MW:15057
scal03175	Listeria innocua Clip11262	transport	0,52	0,00	putative copper-transporting P-type ATPase 3827611:3828015 reverse MW:14665
scal03435	Magnetococcus sp. MC-1	transport	0,37	0,00	TonB-dependent copper receptor [Magnetococcus sp. MC-1] gb|ABK46045.1| TonB-dependent copper receptor [Magnetococcus sp. MC-1] 4150919:4153024 reverse MW:77825
scal00961	Pseudomonas aeruginosa PA	transport	0,34	0,56	putative ABC-type transport protein involved in gliding motility 1128485:1130485 forward MW:74756
scal00713	Desulfuromonas acetoxidans	transport	0,25	0,00	putative ion transport protein 833204:834058 forward MW:31862
scal01187	Oceanobacter sp. RED65	transport	0,14	0,00	putative sulfate transport protein CysZ [Oceanobacter sp. RED65] gb|EAT13806.1| putative sulfate transport protein CysZ [Oceanobacter sp. RED65] 1401043:1401768 reverse MW:26896
scal02901	Alteromonadales bacterium T	transport	0,14	0,00	putative permease 3511144:3512235 reverse MW:39130
scal02650	Flavobacterium johnsoniae U	transport	0,11	0,00	putative copper-transporting P-type ATPase 3221314:3221643 reverse MW:11877
scal00929	Vibrio harveyi HY01	transport	0,09	0,00	permease of the major facilitator superfamily [Vibrio harveyi HY01] gb|EDL68557.1| permease of the major facilitator superfamily [Vibrio harveyi HY01] 1095581:1096318 forward MW:26368
scal01452	Treponema denticola ATCC 3	transport	0,05	0,00	putative ABC transport protein 1746583:1747383 forward MW:30229
scal03432	Geobacter uraniumreducens	transport	0,02	0,00	TonB family protein [Geobacter uraniumreducens Rf4] gb|ABQ24981.1| TonB family protein [Geobacter uraniumreducens Rf4] 4149111:4149887 reverse MW:29172
orf06075	Geobacter uraniireducens	transport	0,02	0,00	TonB family protein [Geobacter uraniireducens Rf4] gb|ABQ24981.1| TonB family protein [Geobacter uraniireducens Rf4] 4149111:4149788 reverse MW:25324
scal00044	Marinomonas sp. MED121	transport	0,02	0,00	putative sodium dependent transporter 51474:52406 forward MW:33776
scal01468	Thermosynechococcus elong	transport	0,01	0,00	putative potassium channel protein [Thermosynechococcus elongatus BP-1] dbj|BAC10007.1| tll2456 [Thermosynechococcus elongatus BP-1] 1762273:1763283 forward MW:37177
scal03050	Shewanella sediminis HAW-E	transport	0,01	0,00	sodium/hydrogen exchanger [Shewanella sediminis HAW-EB3] gb|EDM91396.1| sodium/hydrogen exchanger [Shewanella sediminis HAW-EB3] 3693418:3693885 reverse MW:17539
scal03115	Clostridium phytofermentans	transport	0,01	0,00	Sodium/hydrogen exchanger [Clostridium phytofermentans ISDg] gb|EAT24493.1| Sodium/hydrogen exchanger [Clostridium phytofermentans ISDg] 3765981:3767150 reverse MW:41522
scal03351	Vibrio shilonii AK1	transport	0,01	0,00	putative potassium channel protein [Vibrio shilonii AK1] gb|EDL52981.1| putative potassium channel protein [Vibrio shilonii AK1] 4041476:4041919 reverse MW:16924
scal04263	Desulfotomaculum reducens	transport	0,01	0,00	putative permease 5086401:5087990 forward MW:58509
scal01784	Psychroflexus torquis ATCC 7	transport	0,00	0,00	outer membrane protein, Haemagluttinin-like [Psychroflexus torquis ATCC 700755] gb|EAS73109.1| outer membrane protein, Haemagluttinin-like [Psychroflexus torquis ATCC 700755] 2111096:2111497
scal03482	Hahella chejuensis KCTC 239	transport	0,00	0,00	Mg/Co/Ni transporter MgtE (contains CBS domain) [Hahella chejuensis KCTC 2396] gb|ABC30456.1| Mg/Co/Ni transporter MgtE (contains CBS domain) [Hahella chejuensis KCTC 2396] 4208630:420885
scal00343	Methanospirillum hungatei JF	unknown	13,94	0,00	expressed hypothetical cysteine rich protein 410397:410762 reverse MW:13388
scal02080	Paramecium tetraurelia	unknown	2,33	1,28	expressed hypothetical protein 2530278:2530907 forward MW:23327
scal02735	Vibrio vulnificus YJ016	unknown	1,62	0,43	expressed conserved hypothetical protein 3328216:3329097 forward MW:32564
scal00218	#N/A	unknown	1,44	0,00	expressed unknown protein 270025:270657 forward MW:24167
scal00094	Saccharopolyspora erythraea	unknown	0,57	0,26	expressed protein with unknown function 105616:107916 forward MW:84726
scal00078	Saccharopolyspora erythraea	unknown	0,52	0,00	expressed protein with unknown function 87431:88330 forward MW:33900
scal00170	Silicibacter pomeroyi DSS-3	unknown	0,39	0,78	expressed partial PKD domain protein 206198:207094 forward MW:30910
scal00171	Geobacter bemidjiensis Bem	unknown	0,25	0,22	expressed conserved hypothetical protein 207329:208054 forward MW:26783
scal00204	Vitis vinifera	unknown	0,01	0,00	putative cyanate hydratase 249870:250244 forward MW:13845
scal03998	Nitrosospira multiformis ATC	use of amino acids/peptides	0,55	0,00	oligopeptide ABC transport protein 4799419:4801638 forward MW:85555
scal03731	Chlorobium phaeobacteroides	use of amino acids/peptides	0,46	0,00	Alanine dehydrogenase/PNT, C-terminal:Alanine dehydrogenase/PNT, N-terminal [Chlorobium phaeobacteroides BS1] gb|EAM62220.1| Alanine dehydrogenase/PNT, C-terminal:Alanine dehydrogenase/F
scal04000	Nitrococcus mobilis Nb-231	use of amino acids/peptides	0,37	0,00	oligopeptide ABC transport protein partial 4802786:4803934 forward MW:42970
scal01381	Solibacter usitatus Ellin6076	use of amino acids/peptides	0,27	0,00	Aspartate--ammonia ligase [Solibacter usitatus Ellin6076] gb|ABJ87488.1| Aspartate--ammonia ligase [Solibacter usitatus Ellin6076] 1655874:1656806 forward MW:35794
scal00331	Clostridium novyi NT	use of amino acids/peptides	0,25	0,00	oligopeptide transporter, OPT family [Clostridium novyi NT] gb|ABK62626.1| oligopeptide transporter, OPT family [Clostridium novyi NT] 400357:402300 forward MW:69507
scal00621	marine gamma proteobacteriu	use of amino acids/peptides	0,23	0,00	oligopeptide ABC transporter, periplasmic oligopeptide-binding protein [marine gamma proteobacterium HTCC2143] gb|EAW31858.1| oligopeptide ABC transporter, periplasmic oligopeptide-binding protei
scal00623	Hahella chejuensis KCTC 239	use of amino acids/peptides	0,21	0,00	ABC-type dipeptide/oligopeptide/nickel transport system, permease components [Hahella chejuensis KCTC 2396] gb|ABC27600.1| ABC-type dipeptide/oligopeptide/nickel transport system, permease com
scal03999	Nitrococcus mobilis Nb-231	use of amino acids/peptides	0,21	0,00	oligopeptide ABC transport protein 4801635:4802621 forward MW:37627
scal03162	Petrotoga mobilis SJ95	use of amino acids/peptides	0,01	0,00	ABC-2 type transporter [Petrotoga mobilis SJ95] gb|EDJ81952.1| ABC-2 type transporter [Petrotoga mobilis SJ95] 3808081:3808893 forward MW:30132
scal01427	Nitrococcus mobilis Nb-231	use of amino acids/peptides	0,00	0,00	putative zinc metalloendopeptidase [Nitrococcus mobilis Nb-231] gb|EAR21986.1| putative zinc metalloendopeptidase [Nitrococcus mobilis Nb-231] 1720124:1721275 forward MW:41353
scal02050	Synechococcus sp. CC9311	use of amino acids/peptides	0,00	0,00	asparagine synthase (glutamine-hydrolyzing) [Synechococcus sp. CC9311] gb|ABI46358.1| asparagine synthase (glutamine-hydrolyzing) [Synechococcus sp. CC9311] 2496285:2497052 forward MW:293
scal03766	Crocosphaera watsonii WH 8	use of amino acids/peptides	0,00	0,00	putative peptidase 4531744:4532322 reverse MW:21993
scal03767	Crocosphaera watsonii WH 8	use of amino acids/peptides	0,00	0,00	putative peptidase 4533052:4534665 reverse MW:56947
