# Supplementary material for: The metagenome of the marine anammox bacterium ‘Candidatus Scalindua profunda’ illustrates the versatility of this globally important nitrogen cycle bacterium
Source: Environ Microbiol. 2013 May;15(5):1275–89. doi: 10.1111/j.1462-2920.2012.02774.x (PMC3655542; doi:10.1111/j.1462-2920.2012.02774.x)
Supplement: Supplementary file 14 [file emi0015-1275-SD14.pdf]

Table Supplement 5

number of predicted CxxCH sites	number of matching ORFs
12	2
11	2
10	1
9	0
8	10
7	1
6	1
5	4
4	5
3	1
2	11
1	47
