# Supplementary material for: The metagenome of the marine anammox bacterium ‘Candidatus Scalindua profunda’ illustrates the versatility of this globally important nitrogen cycle bacterium
Source: Environ Microbiol. 2013 May;15(5):1275–89. doi: 10.1111/j.1462-2920.2012.02774.x (PMC3655542; doi:10.1111/j.1462-2920.2012.02774.x)
Supplement: Supplementary file 15 [file emi0015-1275-SD15.pdf]

Supplement table SNP detection scal03925 hydrazine oxidase

Mapping	Reference Position	Reference	Allele Variations	Frequencies	Counts	Coverage	Amino Acid Change
scal03295c	78	A	A/G	56,4/42,6	53/40	94	not resolved
scal03295c	498	A	G/A	57,0/43,0	61/46	107	not resolved
scal03295c	546	T	C/T	58,9/41,1	103/72	175	not resolved
scal03295c	645	G	A/G	54,0/45,7	177/150	328	not resolved
scal03295c	648	T	C/T	53,8/46,2	178/153	331	not resolved
scal03295c	735	T	T/C	64,1/35,5	139/77	217	not resolved
scal03295c	882	G	A/G	53,8/45,5	84/71	156	not resolved
scal03295c	954	T	T/C	62,1/37,6	246/149	396	not resolved
scal03295c	1086	T	T/G	58,5/41,5	96/68	164	not resolved
scal03295c	1095	T	T/G	57,5/41,8	84/61	146	not resolved
scal03295c	1248	A	A/G	62,1/37,7	316/192	509	not resolved
scal03295c	1257	A	A/G	63,1/36,7	313/182	496	not resolved
scal00421	1406	T	T/G	59,4/37,5	a19/12	32	not resolved
scal01317c	none	none	none	none	none	none	none
scal01701c	none	none	none	none	none	none	none
scal02110	566	C	A/C	61,3/38,7	84/53	137	not resolved
scal02116	977	T	T/G	56,2/43,8	a9/7	16	not resolved
scal03974	none	none	none	none	none	none	none
scal04133c	none	none	none	none	none	none	none
scal04164c	798	T	G/T	51,9/48,1	27/25	52	not resolved
scal04164c	594	C	T/A	56,0/44,0	79/62	141	not resolved
